# Two New Fluorinated Phenol Derivatives Pyridine Schiff Bases: Synthesis, Spectral, Theoretical Characterization, Inclusion in Epichlorohydrin-β-Cyclodextrin Polymer, and Antifungal Effect

**DOI:** 10.3389/fchem.2018.00312

**Published:** 2018-07-30

**Authors:** Alexander Carreño, Leonardo Rodríguez, Dayán Páez-Hernández, Rudy Martin-Trasanco, César Zúñiga, Diego P. Oyarzún, Manuel Gacitúa, Eduardo Schott, Ramiro Arratia-Pérez, Juan A. Fuentes

**Affiliations:** ^1^Center of Applied Nanosciences, Universidad Andres Bello, Santiago, Chile; ^2^Fondo Nacional de Desarrollo Científico y Tecnológico, Santiago, Chile; ^3^Laboratorio de Genética y Patogénesis Bacteriana, Facultad de Ciencias de la Vida, Universidad Andrés Bello, Santiago, Chile; ^4^Facultad de Química y Biología, USACH, Santiago, Chile; ^5^Departamento de Química Inorgánica, Facultad de Química, Pontificia Universidad Católica de Chile, Santiago, Chile

**Keywords:** schiff base, intramolecular hydrogen bonds, DFT calculations, epichlorohydrin-β-cyclodextrin polymer, antifungal agents, *Cryptococcus*

## Abstract

It has been reported that the structure of the Schiff bases is fundamental for their function in biomedical applications. Pyridine Schiff bases are characterized by the presence of a pyridine and a phenolic ring, connected by an azomethine group. In this case, the nitrogen present in the pyridine is responsible for antifungal effects, where the phenolic ring may be also participating in this bioactivity. In this study, we synthesized two new pyridine Schiff Bases: (*E*)-2-[(3-Amino-pyridin-4-ylimino)-methyl]-4,6-difluoro-phenol (**F1**) and (*E*)- 2-[(3-Amino-pyridin-4-ylimino)-methyl]-6-fluoro-phenol (**F2**), which only differ in the fluorine substitutions in the phenolic ring. We fully characterized both **F1** and **F2** by FTIR, UV-vis, ^1^H; ^13^C; ^19^F-NMR, DEPT, HHCOSY, TOCSY, and cyclic voltammetry, as well as by computational studies (DFT), and NBO analysis. In addition, we assessed the antifungal activity of both **F1** (two fluorine substitution at positions 4 and 6 in the phenolic ring) and **F2** (one fluorine substitution at position 6 in the phenolic ring) against yeasts. We found that only **F1** exerted a clear antifungal activity, showing that, for these kind of Schiff bases, the phenolic ring substitutions can modulate biological properties. In addition, we included **F1** and **F2** into in epichlorohydrin-β-cyclodextrin polymer (**βCD**), where the Schiff bases remained inside the **βCD** as determined by the *k*_*i*_, TGA, DSC, and S_BET_. We found that the inclusion in **βCD** improved the solubility in aqueous media and the antifungal activity of both **F1** and **F2**, revealing antimicrobial effects normally hidden by the presence of common solvents (e.g., DMSO) with some cellular inhibitory activity. The study of structural prerequisites for antimicrobial activity, and the inclusion in polymers to improve solubility, is important for the design of new drugs.

## Introduction

Invasive fungal diseases are associated with high mortality and morbidity, especially in immunocompromised patients (Edmond et al., 1999; Minari et al., 2002). Complicated fungal infections are often produced by *Candida albicans* and *Cryptococcus* spp., two unicellular fungi (i.e., yeasts; Boral et al., 2017). At present, the most used antifungal agents include triazoles, which exhibit good antifungal activity and broad spectrum of action (Sheehan et al., 1999). Triazoles works by inhibiting the lanosterol 14α-demethylase, a member of the CYP51 class of cytochrome P450 enzymes involved in ergosterol biosynthesis in fungi (Lepesheva and Waterman, 2011; Sagatova et al., 2015). Inhibition of 14α-demethylase leads to the depletion of ergosterol (affecting membrane fluidity) and accumulation of toxic metabolites (e.g., 14α-methyl-3,6-diol; Watson et al., 1989). Triazoles, and some other nitrogen-containing heteroaromatic compounds (e.g., pyridine), have been reported to inhibit CYP51 enzymes by direct coordination of nitrogen with the heme iron (type II ligands) (Hitchcock et al., 1990; Lepesheva et al., 2008; Carreño et al., 2018). The combined effects of ergosterol depletion and toxic metabolite accumulation are fungistatic for many pathogenic fungi, including *C. albicans* and *Cryptococcus* spp. (Mazu et al., 2016). Unfortunately, the excessive use of azoles has led to development of severe resistance, which significantly reduced their efficacy (Hoffman et al., 2000; Casalinuovo et al., 2004), remarking the need of new, efficient antifungal agents.

Schiff bases with different substituents around the azomethine generates a wide variety of organic compounds exhibiting several interesting properties in diverse areas (Jana et al., 2012; Yu et al., 2016), including applications as antimicrobial compounds (Jarrahpour et al., 2007; Justin Dhanaraj and Sivasankaran Nair, 2009).

Previously, we described pyridine Schiff bases that are constituted by a pyridine ring and a phenolic ring connected by an azomethine group. This kind of Schiff bases harbor an intramolecular hydrogen bond (IHB) that provides stability (Carreño et al., 2015, 2018). As stated above, some nitrogen-containing aromatic compounds, such as pyridine Schiff bases, might present antifungal properties (Lepesheva et al., 2008; Carreño et al., 2018). Accordingly, we found that the pyridine Schiff base (*E*)-2-{[(3-aminopyridin-4-yl)imino]-methyl}-4,6-di-*tert*-butyl-phenol (**L2**) demonstrated antifungal activity against *Cryptococcus* spp. (Carreño et al., 2015). Nevertheless, the mere presence of a pyridine is not sufficient to exert the antifungal activity since other similar pyridine Schiff bases exhibited poor or any effect, suggesting that the phenolic ring is also contributing (Carreño et al., 2015, 2018). In this sense, some non-pyridine Schiff bases harboring a phenyl moiety substituted with halogens showed a promising antifungal activity, although other similar bases presented less pronounced effects (Karthikeyan et al., 2006). This evidence shows that the Schiff base structure is fundamental for the biological function (Guo et al., 2007; Carreño et al., 2018).

To improve drugs and other compounds, it has been observed that fluorine substitution can alter chemical properties, disposition, and biological activity of compounds by affecting lipophilicity (Park et al., 2001). Changes in lipophilicity influence partitioning of compounds into membranes, potentially modulating hydrophobic interactions with either receptors or enzymes (Park et al., 2001; Luzina and Popov, 2013). On the other hand, position of the fluorine atom in the aromatic ring can also determine receptor selectivity (Kirk et al., 1986). Thus, both the presence of fluorine and its position in the aromatic moiety could be relevant in the design of new effective antifungal compounds. In this sense, several triazoles share a 2,4-di-fluorine phenyl substituent (Pore et al., 2015), suggesting that this moiety could contribute to the bioactivity of these antifungal agents.

**Figure 1:**
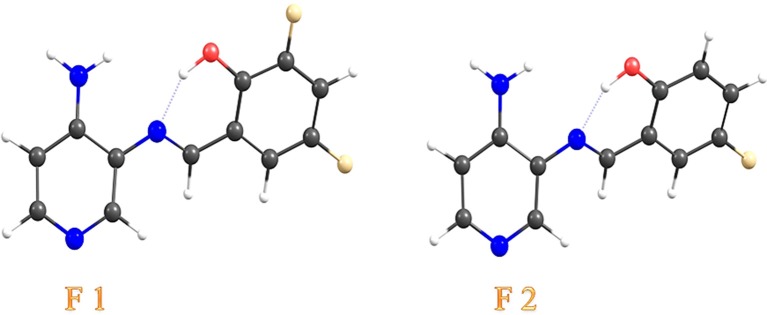
Molecular models of the (*E*)-2-[(3-Amino-pyridin-4-ylimino)-methyl]-4,6-difluoro-phenol (**F1**) and (*E*)- 2-[(3-Amino-pyridin-4-ylimino)-methyl]-6-fluoro-phenol (**F2**), the two pyridine Schiff bases analyzed in this study.

In this study, we focused our attention on the structure-bioactivity relationship of two new pyridine Schiff bases harboring either one or two fluorine substituents in the phenolic ring. These pyridine Schiff bases, (*E*)-2-[(3-Amino-pyridin-4-ylimino)-methyl]-4,6-difluoro-phenol (**F1**) and (*E*)- 2-[(3-Amino-pyridin-4-ylimino)-methyl]-6-fluoro-phenol (**F2**) (Figure 1), were analyzed regarding the structural, optical, and electronic properties, complemented by theoretical calculations (DFT level of theory). Furthermore, we assessed the antifungal properties of **F1** and **F2** against *C. albicans* and *Cryptococcus* spp., two yeasts involved in opportunistic infections in humans, potentially with death risk (Taylor-Smith and May, 2016; Boral et al., 2017). To improve biocompatibility, and considering that usually these kind of Schiff bases are prepared in dimethyl sulfoxide (DMSO) for biological tests (Karthikeyan et al., 2006), we also performed inclusion in epichlorohydrin-β-cyclodextrin polymer (**βCD**) (Gidwani and Vyas, 2014). Cyclodextrins (CDs) are cyclic oligosaccharides closed in a ring that allows the formation of inclusion compounds. Versatility and bioadaptability of CDs are useful to increase the solubility in water to improve delivery of drug molecules (Gidwani and Vyas, 2014). In this context, we also performed studies with **F1** and **F2** included in **βCD**. In this work, we found that there is a structure—bioactivity relationship, where the antifungal activity depends on the presence of two fluorine substitutions in the phenolic ring, since only **F1** exhibited a clear antifungal effect comparable to that of fluconazole. Finally, we found that the inclusion of either **F1** or **F2** in **βCD** improved their solubility in aqueous media and enhanced their antifungal activity, allowing the detection of antimicrobial effects normally hidden by the presence of solvents with some cellular inhibitory activity, such as DMSO.

## Experimental, computational methods and biological essays

### Synthesis

All chemicals and solvents were purchased from Aldrich and Merck. All solvents were stored over the appropriate molecular sieves prior to use. For electrochemical analyses, acetonitrile was purged in an inert atmosphere of argon. Synthesis of (*E*)-2-[(3-Amino-pyridin-4-ylimino)-methyl]-4,6-difluoro-phenol (**F1**) and (*E*)-2-[(3-Amino-pyridin-4-ylimino)-methyl]-6-fluoro-phenol (**F2**) was performed by condensation of 3,4-diaminopyridine with 3,5-di-fluorine-2-hydroxy-benzaldehyde (in the case of **F1**) or 5-fluorine-2-hydroxy-benzaldehyde (in the case of **F2**) in 20 ml of methanol. The reaction was stirred for 24 h at room temperature, without the need of temperature and inert atmosphere. The precipitate was filtered and washed with ethanol and diethyl ether (50/50 v:v). Both for **F1** and **F2**, the yellow products were purified by crystallization, and dried under vacuum.

**Synthesis of (*E*)-2-[(3-Amino-pyridin-4-ylimino)-methyl]-4,6-difluoro-phenol (F1):** Yield 78%. m.p. 182.5–183.7°C.

^1^H NMR (400 MHz, DMSO-_d6_, ppm): δ = 6.15 [bs, 2H, NH_2_], 6.66 [d, J = 5.5; 1H; H2], 7.44–748 [m, 2H; H5 and H6], 7.95 [d, J = 5.5 Hz, 1H; H1], 8.04 [s, 1H; H3], 8.89 [s, 1H; H4]. ^13^C NMR (400 MHz, DMSO-_d6_, ppm): δ = 159.33, 148.97, 148.47, 144.81, 144.74, 139.16, 131.81, 122.81, 122.72, 112.27, 111.96, 109.59, 108.39, 108.11, 107.94. DEPT (400 MHz, DMSO-_d6_, ppm): δ = 159.38, 148.43, 139.26, 112.38, 112.08, 109.64, 107.78. ^19^F NMR (400 MHz, DMSO-_d6_, ppm): δ = −122.64, −132.76. FTIR (ATR, cm^−1^): 3,626 and 3,300 νOH; 3,109 νNH_2_; 1,648 νC = N; 1,593 νC = C. UV/VIS: (Dichloromethane, room temperature) λ nm (ε mol^−1^ dm^3^ cm^−1^): 366 (6.29 × 10^3^), 260 (11.59 × 10^3^); (acetonitrile, room temperature,) λ nm (ε mol^−1^ dm^3^ cm^−1^): 362 (7.76 × 103); (DMSO, room temperature) λ nm (ε mol^−1^ dm^3^ cm-^1)^: 374 (7.70 × 10^3^), 262 (14.96 × 10^3^). m/z: calculated for C_12_H_9_N_3_OF_2_ [M^+^]: 249.2; found 249.8.

**Synthesis of (*E*)-2-[(3-Amino-pyridin-4-ylimino)-methyl]-6-fluoro-phenol (F2):** Yield 82%. m.p. 163.4–164.8°C.

^1^H NMR (400 MHz, DMSO-_d6_, ppm): δ = 6.11 [bs, 2H, NH_2_], 6.70 [d, J = 5.5; 1H; H2], 7.03 [dd; J = 9.0 Hz and 4.6 Hz, 1H; H6], 7.31 [td, J = 8.7 and 3.2 Hz, 1H; H7], 7.68 [dd, J = 9.2 Hz and 3.2 Hz; 1H; H5], 7.99 [d, J = 5.5; 1H; H1], 8.05 [s, 1H; H3], 8.89 [s, 1H; H4]. ^13^C NMR (400 MHz, DMSO-_d6_, ppm): δ = 159.55, 156.75, 156.18, 154.42, 148.79, 148.34, 139.11, 132.20, 121.36, 120.44, 120.21, 118.35, 118.28, 116.66, 116.43, 109.52. DEPT (400 MHz, DMSO-_d6_, ppm): δ = 159.37, 148.22, 138.98, 120.44, 118.28, 116.43, 109.60. ^19^F NMR (400 MHz, DMSO-_d6_, ppm): δ = −125.07. FTIR (ATR, cm^−1^): 3,626 and 3,300 νOH; 3109 νNH_2_; 1,648 νC = N; 1,593 νC = C. UV/VIS: (Dichloromethane, room temperature) λ nm (ε mol^−1^ dm^3^ cm^−1^): 364 (9.46 × 10^3^), 274 (10.18 × 10^3^); (Acetonitrile, room temperature) λ nm (ε mol^−1^ dm^3^ cm^−1^): 360 (9.30 × 10^3^); (DMSO, room temperature) λ nm (ε mol^−1^ dm^3^ cm^−1^): 372 (9.90 × 10^3^), 262 (15.80 × 10^3^). m/z: calculated for C_12_H_10_N_3_OF [M^+^]: 231.2; found 231.8.

### Physical measurements

The purity of each new compounds (**F1** and **F2**) was checked by TLC using glass plates pre-coated with SiliaPlate TLC Aluminum Backed TLC supplied by Silicycle as stationary phase and a suitable solvent system as mobile phase (ethyl acetate). The spots were visualized with short wave ultraviolet light (λ = 254 nm) using Spectroline LongLife TM Filter. Melting points were determined on a Stuart Scientific melting point apparatus SMP3 (UK) in open capillary tubes. The molecular ion peak obtained from the experimental m/z data were recorded on SCIEX QTRAP Systems instrument. The ^1^HNMR, ^13^CNMR, DEPT, HHCOSY, and TOCSY spectra were recorded on a Bruker AVANCE 400 spectrometer operating at 400 MHz, at 25°C. Samples were dissolved in deuterated dimethyl sulfoxide (DMSO-_d6_), using tetramethylsilane as internal standard. Chemical shifts are expressed in δ (ppm) units; J-values for ^1^H–^1^H coupling constant are given in Hertz (Hz) and s, d, dd, br refer to singlet, doublet, doublet of doublets, and broad, respectively. FTIR techniques were recorded in an UATR spectrum Two Perkin Elmer. UV-vis spectra were performed using a Shimadzu Model UV-3101 PC UV-vis-NIR scanning spectrophotometer.

Thermal studies were carried out using a Star System 1 thermogravimetric analyzer (TGA) at a heating rate of 10°C/min. DSC measurements were performed using a Mettler Toledo Star System 822e used to determine the glass transition temperature (T_g_) of the ligands, polymer, and inclusion complexes. The T_g_ was measured at a heating rate of 10°C min^−1^. TGA and DSC experiments were conducted under a nitrogen atmosphere. Samples of 2–3 ± 0.1 mg were used for each experiment. Nitrogen adsorption–desorption analysis was measured on a Micromeritics Gemini (Gemini VII 2390, Micromeritics, America).

### Voltammetry methods

For the electrochemical experiments, the working solution contained 10^−3^ mol L^−1^ of the respective compound (**F1** and **F2**) with 10^−1^ mol L^−1^ tetrabutylammonium hexafluorophosphate (TBAPF_6_, supporting electrolyte) in anhydrous CH_3_CN. Prior to each experiment, the working solution was purged with high purity argon, and an argon atmosphere was maintained during the whole experiment, as previously reported (del Valle et al., 2009). A polycrystalline non-annealed platinum disc (2 mm diameter) was used as the working electrode. A platinum gauze of a large geometrical area, separated from the cell's main compartment by a fine sintered glass, was used as the counter electrode. All potentials quoted in this paper are referred to an Ag/AgCl electrode in tetramethylammonium chloride to match the potential of a saturated calomel electrode (SCE) at room temperature. All electrochemical experiments were performed at room temperature on a CHI900B bipotentiostat interfaced to a PC running the CHI 9.12 software that allowed experimental control and data acquisition.

### Polymer loading and A_L_-phase solubility diagram

Polymer loading was achieved by adding 100 mg of epichlorohydrin-β-cyclodextrin polymer (**βCD**) polymer to a saturated solution of Schiff bases (10 mg of either **F1** or **F2** in 5 mL of ethanol) and stirred for 24 h, obtaining a 9.09% w/w of the corresponding Schiff base/cyclodextrin. The resulting solution were concentrated to 1 mL in a rotary evaporator and then lyophilized. Precipitation of either **F1** or **F2** was not observed during the concentration process, which indicates that both **F1** and **F2** were included in **βCD**. Saturated solutions of either **F1** or **F2** were prepared in an aqueous solution of **βCD** (1 mL in the 0–100 mg mass range) and stirred at 30°C until the equilibrium was reached (24 h). Solutions were centrifuged, and the concentration was determined spectrophotometrically at 365 nm for both **F1** and **F2**. The apparent inclusion constant of the Schiff Base–**βCD** complex (i.e., **βF1** and **βF2**) were determined from the A_L_-phase solubility diagram according to Equation (1).

$$\frac{{\left[D\right]}_{t}-S_{0}}{{\left[\beta CD\right]}_{t}}=\frac{k_{i}S_{0}}{1+k_{i}S_{0}}$$

Where [*D*]_*t*_ corresponded to the total concentration of the guest (mol/L); [**βCD**]_*t*_ corresponded to the total concentration of cyclodextrin in the polymer (mol/L), *S*_0_ (mol/L) was the intrinsic solubility of the guest in water. Value of *k_i_* was determined from the slope (m) of the A_L_-phase solubility diagram (Equation 2).

$$k_{i}=\frac{m}{S_{0}(1-m)}$$

**F1** and **F2** included in β-CDP were named **βF1** and **βF2**, respectively.

### DFT calculations

All structural and electronic properties were obtained using the Amsterdam Density Functional (ADF) code (Te Velde et al., 2001). All molecular structures were fully optimized by an analytical energy gradient method as implemented by Verluis and Ziegler using the hybrid B3LYP functional and the standard Slater-type-orbital (STO) basis set with triple-z quality double plus polarization functions (TZ2P) for all the atoms (Stephens et al., 1994; Ramírez-Tagle et al., 2010; Cohen et al., 2012; Alvarado-Soto and Ramirez-Tagle, 2015; Bjorgaard et al., 2015). Frequency analyses were performed after geometry optimization to corroborate the minimum and to compare with experimental infrared spectra. Time-dependent density functional theory (TDDFT) was used at the same level of theory to calculate excitation energy with the conductor-like screening model for realistic solvents (COSMO) (Sinnecker et al., 2006; Mosquera and Wasserman, 2015; Simpson et al., 2015; Yamin et al., 2016). Three different media were considered for calculations (dichloromethane, acetonitrile, and DMSO) to estimate the hydrogen bond (IHB) stability and to visualize the possible conformational changes due to the solvent polarity. Additionally, calculations were also performed in the gas phase. Natural bond orbital (NBOs) analysis was used to characterize energy of the IHB (Avilés-Moreno et al., 2017; Guajardo Maturana et al., 2017).

### Antimicrobial activity against yeasts

**F1** and **F2**, as well as the corresponding compounds included in **βCD** (i.e., **βF1** and **βF2**) were evaluated for their *in vitro* growth inhibitory activity against the clinical yeasts *Cryptococcus* spp. and *C. albicans*, obtained from the Hospital Clínico of the Universidad de Chile, Santiago, Chile. Minimum inhibitory concentration (MIC) was obtained by broth dilution as described (Cuenca-Estrella et al., 2003). The MIC is defined as the lowest concentration of the tested compounds at which no growth of the strain was observed after the incubation (Cuenca-Estrella et al., 2003). *Cryptococcus* spp. and *C. albicans* were previously cultured in Sabouraud agar (Bacto peptone, 10 g/L; glucose, 40 g/L; agar, 15 g/L; pH 5.6) at 28°C. Further dilutions of microorganisms (0.5 McFarland) were performed with Bacto Tryptic Soy broth (pancreatic digest casein 17.0 g/L, papaic digest of soybean 3.0 g/L, dextrose 2.5 g/L, sodium chloride 5.0 g/L, dipotassium phosphate 2.5 g/L). Stock solutions of the tested compounds were prepared in dimethyl sulfoxide (DMSO) for **F1**, **F2**, and fluconazole (**K1**, commercial antifungal compound used as control); or in water for **βF1** and **βF2**. The concentration range of the compounds tested was between 1.56 and 200 μg/mL; and 15.63–2,000 μg/mL for **βCD** alone. The inoculated wells were then incubated at 28°C for 24, 48, and 72 h. As control, DMSO alone or **βCD** alone were used when necessary. The MIC values of the tested compounds were obtained as μg/mL. All the experiments were performed in biological triplicate, each in technical triplicate.

### Statistical analysis

All values of analyzed data are presented as mean standard error (SE) from three replications. Statistical analysis included was an unpaired *t*-test. Differences among groups were considered statistically significant when the *p* < 0.05.

## Results and discussion

### Synthesis and characterizations

The chemical structure of **F1** and **F2** is shown in Figure 1. Characteristic constants (molecular weight, yield, melting point, solid color, and Rf are shown in Table S1 in the Supplementary Materials). Purity of both **F1** and **F2** was confirmed by mass spectra (Figures S2, S3 in the Supplementary Materials) and thin layer chromatography (TLC) (Table S1 in the Supplementary Materials). FTIR for **F1** presented bands at 3,483 and 3,298 cm^−1^ assigned as the stretch modes of the –OH group; and the doublet signals at 3,134 and 3,077 cm^−1^ for characteristic stretch modes of the –NH_2_ group (Carreño et al., 2014; Yilmaz et al., 2017). In the case of **F2**, the FTIR spectrum showed signals at 3,483 cm^−1^ (νOH); 3,288 cm^−1^ (νOH); 3,153 and 3,057 cm^−1^ (νNH_2_) and at 2,959 cm^−1^, assigned to the stretch modes of –CH groups (Bukowska, 1979; Singh, 2008; Greve et al., 2012) (Figures S4, S5 in the Supplementary Materials). In both cases, peaks assigned as the azomethine –C = N– frequencies (Giannicchi et al., 2013; Vasanthi and Ravikumar, 2013; Senol and Kaya, 2017), and –C = C– stretching were observed at 1,640 and 1,591 cm^−1^ for **F1**, and at 1,628 and 1,581 cm^−1^ for **F2**, respectively.

The structures of the compounds (**F1** and **F2**) were confirmed by 1D and 2D NMR obtained in DMSO-_d6_ solutions (see Figures S6, S7 in the Supplementary Materials for numbering of protons and carbons, respectively). The ^1^HNMR spectrum for **F1** (Figures S8, S9 in the Supplementary Materials) exhibits the amino proton at 6.15 ppm, whereas the amino proton was observed at 6.11 ppm for **F2** (Figures S10, S11 in the Supplementary Materials), effects that can be explained by the nature of fluorine in the phenolic ring (Carreño et al., 2015). In both **F1** and **F2**, the amino proton signal disappeared from the spectrum after D_2_O exchange (Figures S11, S13 in the Supplementary Materials), confirming the assignation.

HHCOSY was also performed (Figures S14, S15 in the Supplementary Materials). Signals assigned to the aromatic protons of both pyridine and phenolic rings appeared at 7.70–6.50 and 7.99–9.00 ppm for **F1** and **F2**, respectively. In the case of **F1**, for the assignment of H5 and H6 (phenolic ring, see Figure S9 in the Supplementary Materials), we obtained a multiplet (m) between 7.44 and 7.48 ppm. In this context, we performed 1D TOCSY spectrum to complement our analyses and to identify resonances of all the protons in both the pyridine and phenolic rings. Regarding **F1**, when irradiating H5 (Figure S16 in the Supplementary Materials), an enhancement was observed for H6 and, similarly, when H6 was irradiated (Figure S17 in the Supplementary Materials), an enhancement was observed for H5. In the case of protons located in the pyridine ring, when H1 or H2 were irradiated, a spectrum showing enhancement for H3, and the corresponding H1 or H2, were obtained (pyridine ring, see Figures S18, S19 in the Supplementary Materials). All these results indicate that H1, H2, and H3 are located in the pyridine ring (See Figure S6 in the Supplementary Materials). With respect to **F2**, when H5 was irradiated (phenolic ring), enhancements in H6 and H7 were observed (see Figure S20 in the Supplementary Materials). A similar result was obtained when H7 was irradiated (see Figure S21 in the Supplementary Materials), confirming the proton assignment in the phenolic ring. On the other hand, when H2 was irradiated (pyridine ring), it was possible to observe H1 and H3 signals (see Figure S22 in the Supplementary Materials). These experiments allowed us assigning all signals to their respective protons in the proposed structures (Figure 1).

To complement our analyses, ^13^CNMR broad-band decoupled spectrum in DMSO-_d6_ solution was performed to a total peak assignment for **F1** and **F2**, showing a total agreement with the corresponding inferred structures (see Figures S23, S24 in the Supplementary Materials). DEPT experiments allowed us to corroborate **F1** and **F2** structures (see Figures S25, S26 in the Supplementary Materials). The signals observed at 159.23 and at 159.55 ppm were assigned to the carbon of the azomethine group in **F1** and **F2**, respectively, as previously assigned for other Schiff bases (Aranha et al., 2007; Alsaygh et al., 2014).

^19^FNMR spectra showed two signals for **F1** at −122.64 and −132.75 ppm, corresponding to the fluorine atoms in the 4 and 6 positions in the phenolic ring, respectively (see Figure S27 in the Supplementary Materials). In the case of **F2**, only one signal, corresponding to the fluorine at the position 6 in the phenolic ring, appeared at −125.07 ppm (see Figure S28 in the Supplementary Materials). Altogether, these results confirmed the structures proposed for both **F1** and **F2**.

### UV-vis studies

The fluorinated pyridine Schiff bases **F1** and **F2** are water insoluble at room temperature. Nevertheless, they presented low solubility in dichloromethane and acetonitrile, and high solubility in DMSO at room temperature. In this context, we recorded UV-vis in dichloromethane (ε = 8.93), acetonitrile (ε = 37.5), and DMSO (ε = 46.7) at room temperature, three organic solvents exhibiting increasing polarity (Liu et al., 2010; Płowaś et al., 2013) to determine possible changes in spectra. For both **F1** and **F2**, the UV-vis spectra showed a shoulder and a band for both dichloromethane and DMSO (see Figures S29–S34, and Table S2 in the Supplementary Materials). The shoulder, centered approximately at 260 nm, was assigned to n → π^*^ (–C = N–); whereas the band (approximately at 370 nm) was assigned to π → π^*^ transitions, according to previous reports showing other similar compounds (Reddy et al., 2009; Zoubi and Kandil, 2012; Carreño et al., 2015). All these results were summarized in Table S2 in the Supplementary Materials. These results show that, in both apolar (dichloromethane) and polar (acetonitrile) solvents, and independently on the concentration of the respective Schiff base, the UV-vis exhibits a similar pattern, strongly suggesting that the compounds show no significant interaction with the solvents. In this sense, the π → π^*^ transition (related to the IHB, as will be discussed later in the computational studies), remains essentially unchanged, strongly suggesting the high stability of the IHB. On the other hand, we also tested the UV-vis in presence of DMSO, a solvent that could form intermolecular hydrogen bonds with the solutes (Charisiadis et al., 2014; Sigalov et al., 2017), to corroborate the stability of the IHB under this condition. In this case, we observed a better definition the band assigned to n → π^*^ (–C = N–) (~260 nm). Furthermore, the band assigned to π → π^*^ appeared approximately at 370 nm, showing a red-shift of only 10 nm in comparison with the results obtained with the other tested solvents. These results reinforce the strength of the IHB present in the both pyridine Schiff bases, as will be discussed below in the computational studies.

Regarding luminescent properties, neither **F1** nor **F2** exhibit luminescence in solid state and under UV lamp, consistent with previous studies showing characterization of similar compounds harboring an azomethine group (Garcia-Amorós et al., 2010; Carreño et al., 2018).

### DFT and TD-DFT studies

To better understand the electronic and optical properties of both **F1** and **F2**, we performed DFT calculations. In the first place, geometry optimization of the neutral compounds was carried out with the hybrid B3LYP functional (Orio et al., 2009; Cohen et al., 2012) and the standard Slater-type-orbital (STO) basis, set with triple-z quality double plus polarization functions (TZ2P) for all the atoms (Cohen et al., 2012; Lashgari et al., 2016). Since both **F1** and **F2** are new compounds, X-ray crystal data is not available. Nevertheless, geometry optimization analyses showed that the main atom distances of both **F1** and **F2** are in good agreement with the experimental crystallographic data of a previously reported pyridine Schiff base **L1** ((*E*)-2-{[(2-aminopyridin-3-yl)imino]-methyl}-4,6-di-tert-butyl-phenol (see Figure S2 in the Supplementary Materials) (Carreno et al., 2012).

**Figure 2:**
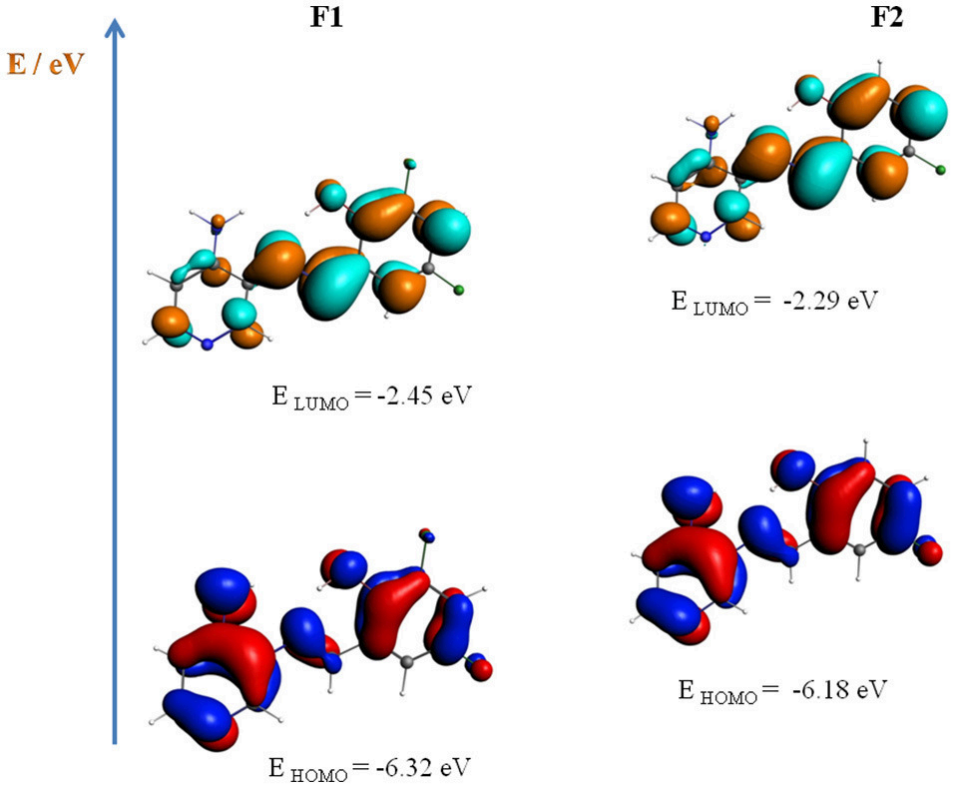
Molecular models for **F1** and **F2** compounds. The energy of the frontier orbital HOMO-LUMO is presented in eV.

Regarding the IHB, **F1** and **F2** present an OH···N distance of 1.772 and 1.773 Å, respectively (for optimized geometrical parameters, see Table S3 in the Supplementary Materials). With respect to the conformational structure, both **F1** and **F2** adopt a non-coplanar conformation, with a dihedral angle between the pyridine and the phenolic ring of 40.1 and 43.2°, respectively. Figure 2 shows the geometry optimizations of **F1** and **F2**, including the energy of the frontier orbital HOMO-LUMO in eV (E_HOMO−LUMO_ = 3.87 eV for **F1**, and 3.89 eV for **F2**). The calculated frequencies are congruent with the values observed in the FTIR spectra for the most important functional groups. All the frequencies were positive, which means that the minimum was found. In general, there is no difference between the frequencies of both compounds except for the azomethine vibration, which appears shifted in 30 cm^−1^ for **F1** (see Figures S35, S36, and Table S4 in the Supplementary Materials) (Carreño et al., 2015).

To further study the IHB of both **F1** and **F2**, we evaluated the second-order interaction energy by means of Natural Bond Orbitals (NBOs) calculations (Deshmukh et al., 2006; Weinhold, 2012). The interaction energy is related to stabilizing donor-acceptor interactions due to electron delocalization with respect to the zeroth-order natural Lewis structure (Purser, 1999; Jesus et al., 2008). In addition, NBO analysis provides an efficient method for studying participation of hydrogen bond in the stability of N-harboring compounds (Avilés-Moreno et al., 2017). Our analyses indicated that, for both **F1** and **F2**, the IHB acts providing stability between the pyridine and phenolic rings. Based on the energy of the IHB, it is possible to sort the molecules as: **F1** (26.7 kcal/mol) > **F2** (23.5 kcal/mol). It is important to remark that these energies are found in the typical range for this kind of hydrogen bonds (Avilés-Moreno et al., 2017).

On the other hand, and to further elucidate the UV-vis transitions, we conducted time dependent density functional theory (TD-DFT) calculations for both **F1** and **F2**, in the same solvents used for the Figures S29–S34 (see Supplementary Materials). As shown in the Table S5 in the Supplementary Materials, the most important bands were associated to n → π^*^ and π → π^*^, in turn related to azomethine group and to the IHB, respectively (see Figure 2 to complement the analysis). It is remarkable that these results were similar in the three solvents used for analysis and even with the gas phase, with no significant shifting in the expected UV-vis spectra in any case, complementing the experimental data and corroborating the high stability of the IHB.

### Electrochemical studies

**Figure 3:**
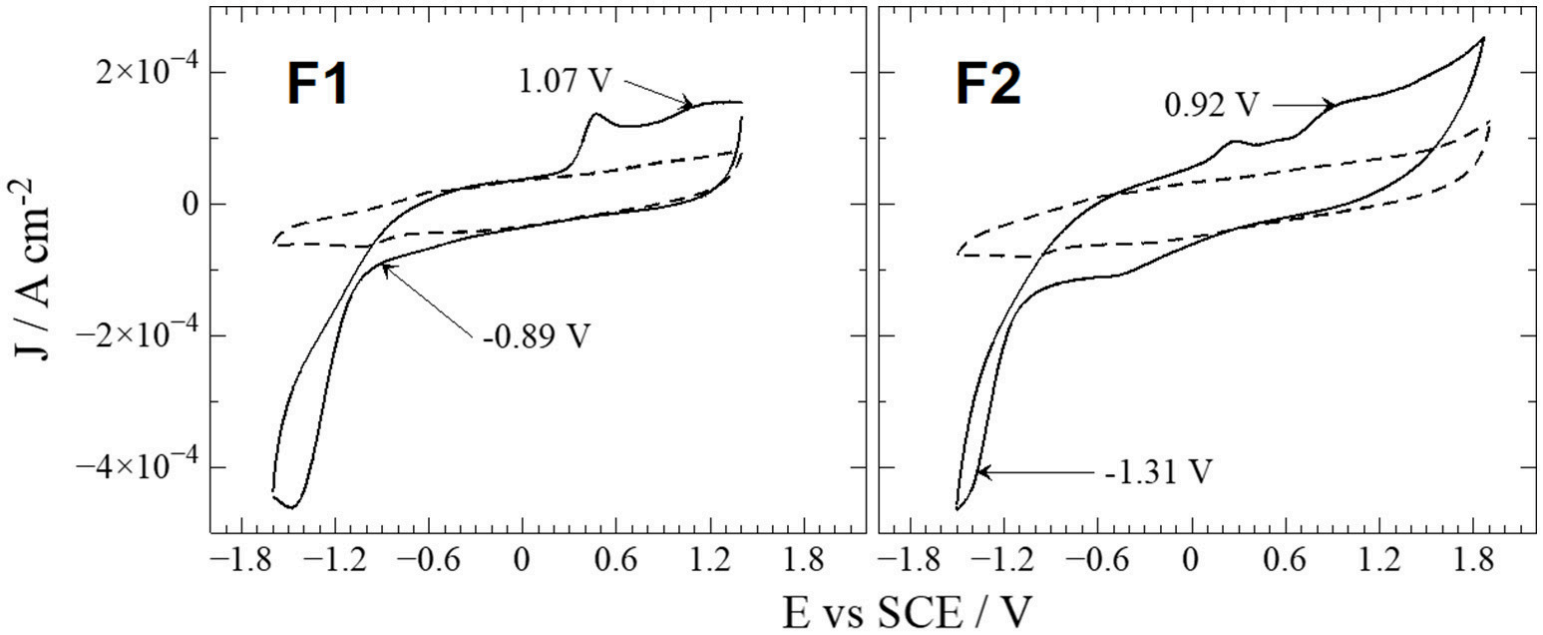
CV profiles of **F1**, **F2** (straight lines) and blank (dashed lines). Interphase: Pt| 0.01 mol L^−1^ of compound + 0.1 mol L^−1^ of TBAPF_6_ in anhydrous CH_3_CN under an argon atmosphere. Scan rate: 200 mV s^−1^.

Electrochemical properties of compounds were studied by observation of redox processes through cyclic voltammetry experiments (Figure 3). Cyclic voltammograms of compounds **F1** and **F2** (straight lines), performed in an anhydrous CH_3_CN under anoxic conditions, showed several signals, different from blank experiments (dashed lines). Nevertheless, it is possible that some of these signals corresponds to secondary processes of by-products formed after the first reduction/oxidation of compounds and/or solvent-molecules at the working window limits, as it has been observed on past studies for other Schiff bases (Carreño et al., 2014, 2015, 2018). Therefore, in order to discriminate which signal corresponds to a compound-original process, it is recommended to perform a working-window potential study (see Figure S37 in the Supplementary Materials). For instance, **F1** displays up to three redox processes, two irreversible oxidations at 0.48 and 1.07, along with an apparent-irreversible reduction at 0.89 V (Figure 3). However, in the working-window study (Figure S37), the signal at 0.48 is only visible when the working window limit gets near its negative limit; thus, it does not correspond to a compound-original process. Moreover, the apparent-irreversible reduction at 0.89 becomes reversible (Figure S37), indicating that the excessive current output of the complete-working window cyclic voltammogram (Figure 3) disturb the correct description of electrochemical processes. After all these analyses and data processing, signal description, potential values, reversible character, and mass-transport control (diffusional) were obtained and summarized on Table 1. Both **F1** and **F2** exhibited secondary signals (around +0.3 V vs. SCE) that were only visible when the working-window reached the most negative potential limit; thus, the electrode surface must and was cleansed between each measurement to assure the obtainment of trustful results.

**Table 1:** Electrochemical signals description for **F1** and **F2**.

**Compound**	**${\text{E}}_{1}^{ox}$**	**${\text{E}}_{1}^{\text{red}}$**
	**V vs. SCE**	
**F1**	+1.07 _(irr−d)_	−0.89 _(qrev−d)_
**F2**	+0.92 _(irr−nd)_	−1.31 _(irr−d)_

*Ox, oxidation; red, reduction; irr, irreversible; qrev, quasi-reversible; d, diffusional; nd, not diffusional*.

At first sight, the signals described on Table 1 describing redox processes might seem too subtle in the Figure 3. Nevertheless, if the scan-rate study is revised (Figure S38), these signals become more pronounced at lower scan-rates, due to negligible effect from double-layer capacitance at slow scans (Brett and Brett, 1993). As reported for similar compounds (Carreño et al., 2014, 2015, 2018), an irreversible oxidation process is always observed. The nature of the irreversible oxidations observed on the compounds is supported by theoretical calculations; the HOMO position determines that this process is typically ascribed to an oxidation taking place somewhere at the pyridine ring, most likely at the –NH_2_ moiety. Reductions, on the other hand, are ascribed for an intramolecular reductive coupling of the azomethine group, which involves a self-protonation reaction reported for similar compounds (Zolezzi et al., 2002; Carreño et al., 2014), which also explains the quasi- and irreversible character of this process. The mass-transport control of the process is defined by the scan rate study results (see Figure S38 and Table S6 in the Supplementary Materials), suggesting that in the experimental conditions all electrochemical processes, except for **F2** oxidation, are controlled by diffusion of species from bulk solution.

### Solubility studies

**Figure 4:**
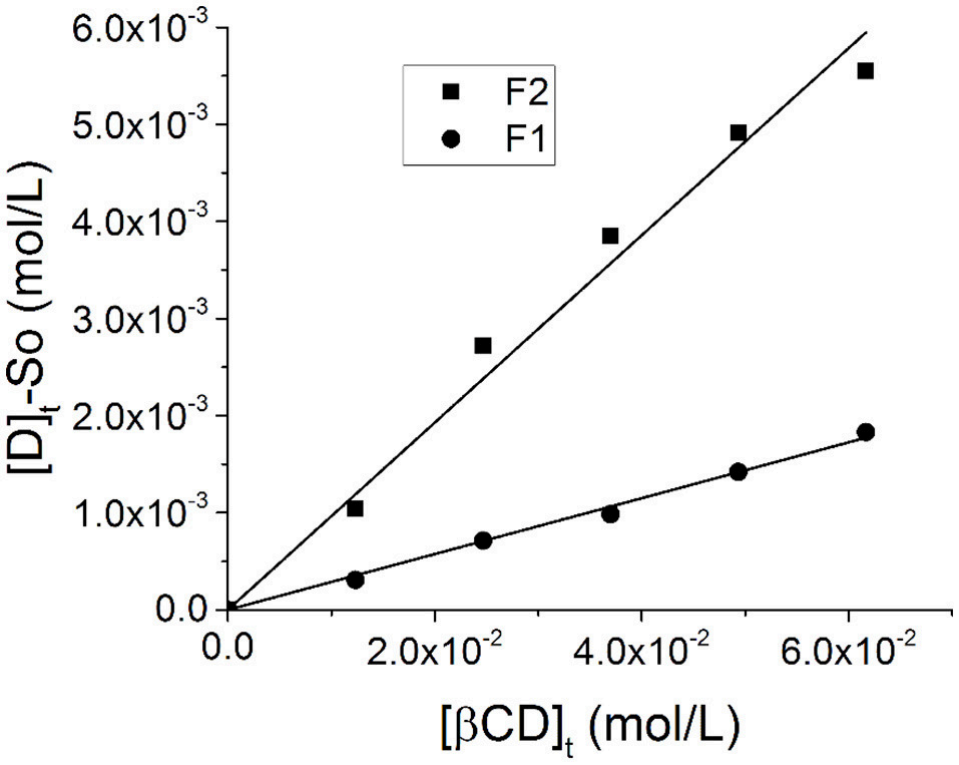
A_L_-Phase solubility diagram of **βF1** (●) and **βF2** (■) in presence of **βCD**. [*D*]_*t*_ corresponded to the total concentration of the guest (mol/L) (i.e., **F1** or **F2**); [**βCD**]_*t*_ corresponded to the total concentration of cyclodextrin in the polymer (mol/L); *S*_0_ (mol/L) was the intrinsic solubility of the guest in water (see **Equation 2**).

As stated, inclusion of Schiff bases in epichlorohydrin-β-cyclodextrine polymer (**βCD**) could improve biocompatibility by increasing solubility of these compounds in aqueous systems (Gidwani and Vyas, 2014). In this context, we included **F1** or **F2** in **βCD**. As the first step, the host-guest interactions were studied in terms of the apparent inclusion constants (*k*_*i*_) of **F1** and **F2** within (or inter) **βCD** cavity. Due to the basic nature of both the pyridine and the amino group present in **F1** and **F2**, we speculated that the formation of the inclusion complexes will preferentially occurs between the phenol moiety of the Schiff bases and the **βCD** cavity. Figure 4 shows A_L_-type phase-solubility diagrams of **F1** + **βCD** (**βF1**) and **F2** + **βCD** (**βF2**). The A_L_-Phase solubility diagram shows a linear dependence between total concentration of the **βCD** with [D]_t_-S_0_. This linearity indicates a 1:1 (guest:host) stoichiometry; albeit this not necessarily implies that all guests (**F1** or **F2**) are included in the **βCD** cavity. In this sense, the micro voids found between the crosslinked chains of the **βCD** (in particular, –OH groups) could form hydrogen bonds with the guests by interactions with the amino groups of the pyridine moiety, contributing to increase the solubility of either **F1** or **F2** (see **Figure 6**). Thus, we hypothesize that Schiff bases are included inside **βCD** cavity (see below). Inclusion constants are shown in Table 2.

**Table 2:** *k*_*i*_ (inclusion constant) calculated from the A_L_-type phase-solubility diagram for **βF1** and **βF2** in **βCD**.

	**βF1**	**βF2**
Slope × 10^2^	2.3 ± 0.8^*^	7.6 ± 0.5
S_0_·10^4^ (mol L^−1^)	5.1 ± 0.9	7.5 ± 0.9
*k_*i*_*	45 ± 13	101 ± 10

* *Standard deviation*.

As can be noted, the *k*_*i*_ value determined for **βF2** is approximately the double than *ki* determined for **βF1** (Table 2). This difference can be explained by the structure of **F1** and **F2**. **F1** has two fluorine atoms as substituents (4- and 6-positions) in the phenolic ring, whereas **F2** has only one fluorine atom (6-position; see Figure 1). In the case of **F1**, the fluorine found at the 6-position could restrict the accommodation of the phenolic ring in the **βCD** cavity, explaining the decreased value of *ki* in this case.

These results show that it is possible to render **F1** and **F2** water-soluble by means of inclusion with **βCD**.

### Thermal studies by TGA and DSC, and nitrogen adsorption–desorption isotherm

**Figure 5:**
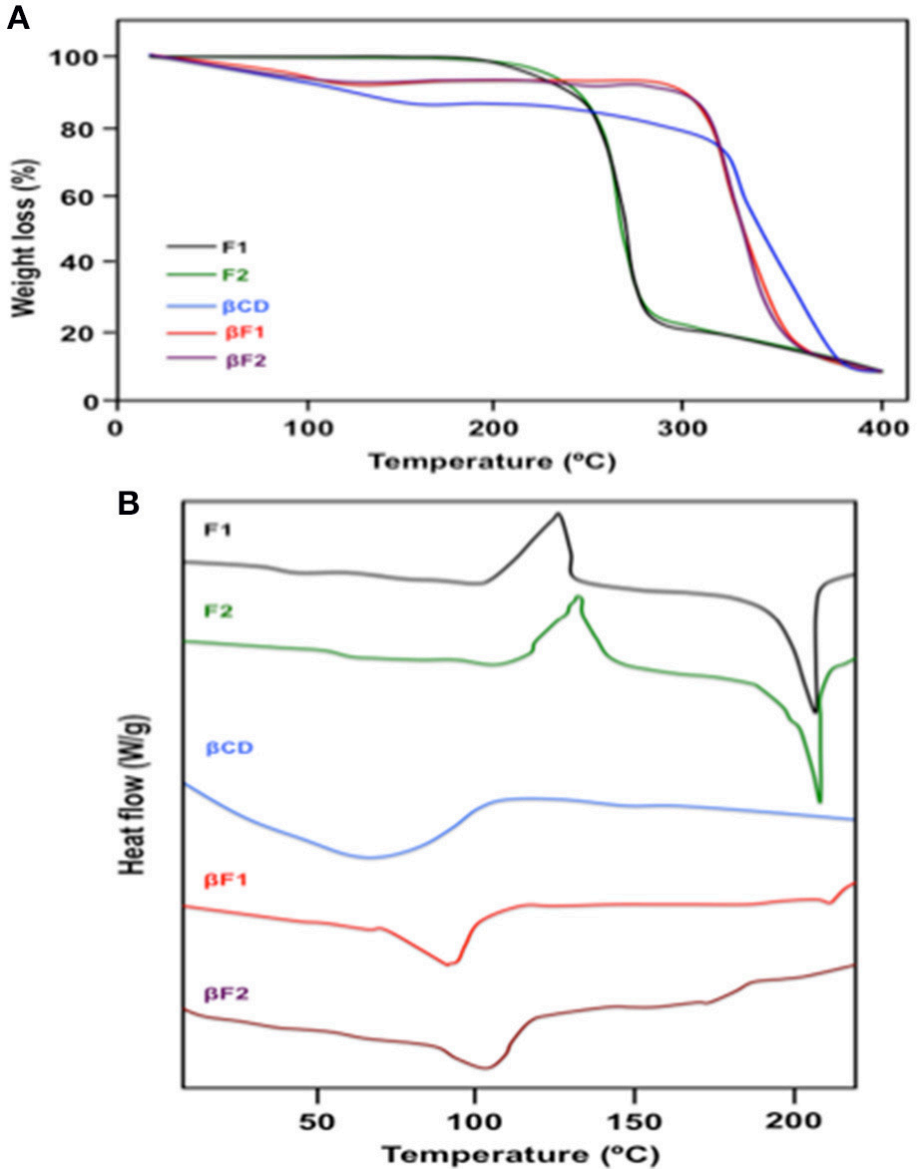
**(A)** Thermogravimetry analysis (TGA), and **(B)** DSC thermograms of **F1**, **F2**, **βCD**, **βF1**, and **βF2**.

To elucidate how **F1** and **F2** are interacting with **βCD**, we performed a physicochemical approach. For this purpose, thermogravimetric analysis (TGA) was used to compare the thermal decomposition temperature (TDT) of the Schiff bases alone (i.e., **F1** or **F2**) and Schiff bases included into **βCD** (i.e., **βF1** or **βF2**). As control, we also tested **βCD** alone. The TGA curves exhibited five decomposition curves in one-step corresponding to **F1**, **F2**, **βCD**, **βF1**, and **βF2** (Figure 5A). For **F1** and **F2**, a weight loss, observed approximately at 220°C, can be attributed to a decomposition of the Schiff if we considered their melting points (see Table S1 in the Supplementary Materials). In the case of **βF1** and **βF2**, a different pattern was found, where ~12.5% of weight loss was observed at 300°C. The **βF1** and **βF2** presented an extrapolated TDT between 330 and 345°C at which point the decomposition of the backbone chain occurs (Figure 5A). As expected, **βCD** alone exhibited a completely different behavior (Figure 5A). Table 3 shows a summary of the main data obtained for TDT.

**Table 3:** Thermal decomposition temperature (TDT).

**Sample**	Composition (%)^*^ (SB:βCD)	**TDT (°C)**	**Weight loss (%) at different temperatures (**^**°**^**C)**			
			**100°C**	**200°C**	**300°C**	**400°C**
**F1**	100:0	241.1	–	–	75.3	91.2
**F2**	100:0	238.4	–	–	77.6	93.4
**βCD**	0:100	308.3	6.43	10.8	18.8	91.9
**βF1**	50:50	345.6	7.87	9.12	13.1	88.6
**βF2**	50:50	330.0	6.95	9.03	12.6	89.1

* *SB, Schiff base (i.e., **F1** or **F2**). Heating rate: 10°C min^−1^*.

Differential Scanning Calorimetry (DSC) measurement is a good technique to analyzing the interaction between host and guest molecules during the complex inclusion formation (Karoyo et al., 2011). When guest molecules are embedded in the **βCD** cavity, their melting points usually shift to a different temperature or disappear (El-Maradny et al., 2008; Sapte and Pore, 2016). The DSC thermograms of **F1**, **F2**, **βCD**, **βF1**, and **βF2** are shown in Figure 5B. The DSC curve of **F1** and **F2** exhibited a weakly discernible glass transition temperature (T_g_) at 46.52 and 53.28°C, respectively. For both compounds, two similar phenomena were observed, a broad exothermic peak with an onset of 110.3°C for **F1** and 118.4°C for **F2**, and an endothermic peak with a start of 183.6°C for **F1** and 167.1°C for **F2**, assigned to a recrystallization and fusion, respectively. These results are in agreement with those obtained in the melting point measurements (see Table S1 in the Supplementary Materials). In the case of **βCD**, DSC showed a weight loss attributed to loss of water from **βCD** cavity (Figure 5B). On the other hand, the inclusion complexes (i.e., **βF1** and **βF2**) showed appearance of broad peaks at 93.76 and 110.84°C, respectively, assignable to water loss and disappearance of T_g_ of **F1** and **F2** (Figure 5B). This result suggests that both **F1** and **F2** are trapped inside **βCD** cavity, with replacement of water molecules. The peaks of **F1** and **F2** also disappeared when compared with **βF1** and **βF2**, respectively, indicating their involvement in the complexation process. These results suggest an existence of strong physical interaction between either **F1** or **F2** with **βCD**, indicating the formation of a stable inclusion complex in solid state. To complement these results and further understand the inclusion phenomenon, we performed a nitrogen adsorption–desorption analysis. This analysis is based on the adsorption of nitrogen on the surface of the tested compounds, as a technique for the measurement of the specific surface area (S_BET_). The analysis of the nitrogen adsorption isotherm profiles for **βF1** and **βF2** at 77 K showed an isotherm of type II (see Figure S39 in the Supplementary Materials) that involves physical adsorption of nitrogen gas by non-porous solids, with a negligible microporous volume and low mesoporous volume. Both **βF1** and **βF2** present a mesoporous volume (pore size) ranging 2–50 nm, according to the IUPAC classification (Table 4).

**Table 4:** Parameters obtained from nitrogen adsorption–desorption analysis at 77 K.

**Material**	***S*_BET_ (m^2^ g^−1^)**	**A (nm)**
**βCD**	1.87	4.37
**βF1**	93.50	3.81
**βF2**	89.56	3.90

*S_BET_, Brunauer–Emmett–Teller Surface area. A, Pore size of the inclusion complexes or **βCD** alone*.

The incorporation of **F1** and **F2** into the **βCD** cavity (i.e., **βF1** and **βF2**) caused a significant increase in nitrogen adsorption accompanied with a decrease in the pore size, compared with **βCD** alone (Table 4). These results show that either **F1** or **F2** incorporation into **βCD** likely occurs inside the pore channels, and not on the **βCD** surface. The guest inside the host improved nitrogen retention due to a steric effect and pore blockage. By contrast, if the guest (i.e., **F1** or **F2**) were been adsorbed on the host surface (i.e., **βCD** surface), we were observed a decrease in the S_BET_ value due to a hindrance produced by the guest, hampering nitrogen adsorption.

**Figure 6:**
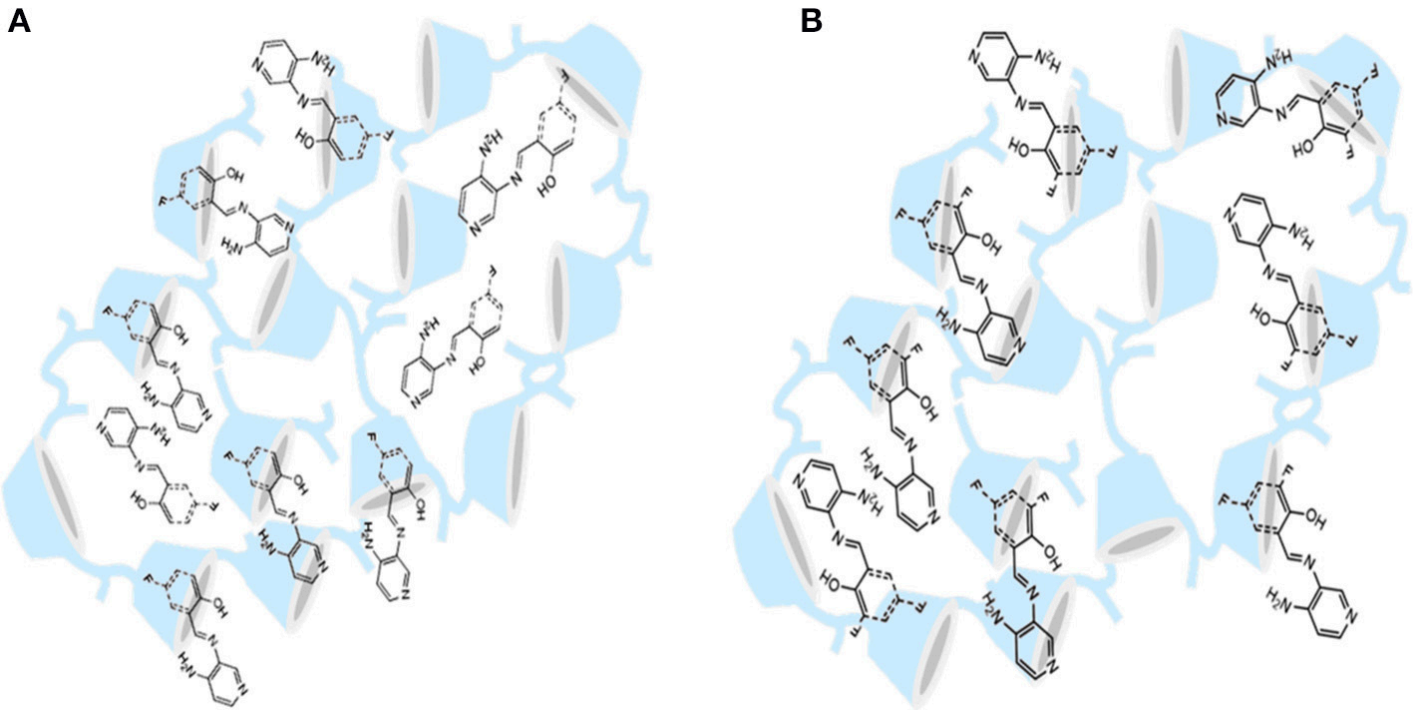
Representation of the inclusion complex of **(A)** **βF2** and **(B)** **βF1** inside **βCD** cavity.

All these results together confirmed that the guests are found inside **βCD**, as previously proposed in the solubility studies (Figure 6).

### Biological activity: antimicrobial activity of the schiff bases (F1 and F2)

As stated, structure of the Schiff bases is fundamental for their biological applications (Carreño et al., 2018). In fact, little changes regarding substituents can produce big effect with respect to the antimicrobial properties of pyridine Schiff bases (Carreño et al., 2015, 2018). In non-pyridine Schiff bases, the use of halogens in the phenyl moiety can positively increase the antifungal activity in some cases (Karthikeyan et al., 2006). In the case of pyridine Schiff bases, it has been reported that the biological activity against fungi relied on the nitrogen atom in the pyridine ring, and this activity can be modulated by different substituents (i.e., chlorine) in the phenolic ring at the 4 and 6 positions (Carreño et al., 2018). In this sense, the use of fluorine as substituent in different drugs has shown to modulate both chemical and biological properties, including membrane partitioning or interaction with their respective targets (Kirk et al., 1986; Kirk and Filler, 1996; Park et al., 2001; Luzina and Popov, 2013). Thus, we tested both **F1** and **F2** regarding their antifungal properties against *C. albicans* and *Cryptococcus* spp., two yeasts that produce systemic infections in immunocompromised patients (Taylor-Smith and May, 2016; Boral et al., 2017). For that, we determined the minimal inhibitory concentration (MIC); compounds with low MIC are considered better antifungal agents. We found that **F1** exerted an antifungal activity against *Cryptococcus* ssp., which was undistinguishable from the activity observed for the known, commercial antifungal fluconazole, at all the times tested (24 h: *p* = 0.73; 48 h: *p* = 0.59; 72 h: *p* = 0.44; Table 5). On the other hand, we found that neither **F1** nor **F2** exerted an antifungal effect against a clinical, fluconazole-resistant strain of *C. albicans* (Table 5), suggesting that the mechanisms involved in fluconazole resistance could be also participating in the **F1** resistance (e.g., efflux pumps; Monapathi et al., 2018). To demonstrate that the presence of fluorine substituents is important for the antifungal activity, we compared the antimicrobial effect of two analogous Schiff bases (**L3** and **L4**, see Figure S1 in the Supplementary Materials) (Carreño et al., 2018) harboring chlorine substituents at the same positions of the fluorine substituents found in **F1** and **F2**, respectively. We found that only **L3** exerted a noticeable antifungal activity against *Cryptococcus* spp. under the tested conditions, whereas **L4** exhibited an effect that was undistinguishable from the effect produced by DMSO alone. These results reinforce the fact that, to obtain efficient antifungal activity, it is necessary the presence of two substitutions in the phenolic ring at positions 4 and 6 (Carreño et al., 2018). Nevertheless, not any substitution is suitable, since **L4** (chlorine) exerts only a fungistatic activity that disappears after 72 h, whereas **F1** (fluorine) seems to produce a fungicide effect, similar to that produced by fluconazole (Table 5). All these results argue for the relative advantage of fluorinated pyridine Schiff bases over other very similar compounds. Considering that **F1** and **F2** only differ in one substituent (the fluorine at position 6 in the phenyl moiety is absent from **F2**), little structural changes can greatly affect the antimicrobial properties of this kind of pyridine Schiff bases. In other words, only one fluorine can make the difference between a fluconazole-like antifungal activity and the lack of antifungal activity. Previously, it has been proposed that, in the case of pyridine Schiff bases, the biological activity depends on the presence of the nitrogen atom in the pyridine ring, and this activity can be modulated by different substituents in the phenolic ring, suggesting that these two rings exert a cooperative effect (Carreño et al., 2015, 2018). Our results underline the importance of the substitutions in the phenolic rings, showing that only **F1** (i.e., two fluorine at position 4 and 6 in the phenolic rings), and not **F2**, exerts an important antifungal effect. It is remarkable that synthetic imidazole antifungal drugs, such as fluconazole, present a nitrogen-containing heteroaromatic ring (e.g., imidazole) as well as a phenyl moiety substituted with two fluorines.

**Table 5:** Minimal inhibition concentration (μg/mL) of tested compounds.

	**Concentration (μg/mL) ± SE**					
	***Cryptococcus*** **spp**.			***Candida albicans***		
**Compound**	**24 h**	**48 h**	**72 h**	**24 h**	**48 h**	**72 h**
**F1**	6.5 ± 1.2^*^	7.8 ± 0.5^*^	7.4 ± 0.5^*^	–	–	–
**F2**	–	–	–	–	–	–
**L3**	ND	33.3 ± 14.4	–	ND	–	–
**L4**	ND	–	–	ND	–	–
Fluconazole	5.8 ± 1.4	9.2 ± 2.3	10.2 ± 3.2	–	–	–

SE, Standard error; –, Undistinguishable from DMSO alone (i.e., no effect); ND, Not determined;

* ***F1** effect is undistinguishable from fluconazole effect against Cryptococcus spp., as assessed by t-test (p > 0.40 at all the times tested)*.

In order to better understand the effect of both **F1** and **F2** in more physiological conditions, we included these compounds in epichlorohydrin-β-cyclodextrin polymer (**βCD**) (see above) (Gidwani and Vyas, 2014). Cyclodextrins (CDs) are cyclic oligosaccharides formed by monomers of D-glucopyranose bound together by α-1,4-glucosidic linkages. These structures form a rigid, truncated, cone-shaped structure with an internal cavity (5–8 Å) that allows the inclusion of molecules, as stated above. Depending on the number of monomers, CDs may be classified into α (six glucose units), β (seven glucose units), or γ (eight glucose units) (Gidwani and Vyas, 2014; Duchêne and Bochot, 2016). CDs are useful for a broad of applications, especially in drug delivery, in part because they enhance biocompatibility by increasing water solubility (Gidwani and Vyas, 2014). In this context, we also performed studies with **F1** and **F2** included in **βCD** (called **βF1** and **βF2**, respectively). Unlike **F1** and **F2**, which were prepared in DMSO, **βF1** and **βF2** were dissolved in water due to their improved solubility in aqueous solvents (Figure 4). As expected, **βF1** exerted an antifungal effect against *Cryptococcus* spp. Interestingly, the inclusion of **F1** in **βCD** revealed an antimicrobial, previously undetected effect against *C. albicans* (Table 6), suggesting that the water solubility contribute to its biological activity. On the other hand, we also observed a consistent fungistatic effect against both *Cryptococcus* spp. and *C. albicans* produced by **βF2**. As stated above, **F2** exerted an antifungal effect that was undistinguishable from DMSO alone (Table 6). Although DMSO is extensively used in pharmaceutical, chemical, and biomedical applications, this solvent exerts an inhibitory effect against eukaryotic cells, including yeasts, by impairing cell replication or increasing cell apoptosis (Cho et al., 2014; Kakolyri et al., 2016). In this sense, DMSO could be screening the antimicrobial effect of **F2**, which was revealed with **βF2** dissolved in water. Accordingly, **F1** and DMSO alone were undistinguishable regarding the antimicrobial effect against *C. albicans* (Table 5). By contrast, **βF1** clearly exhibited an antifungal effect against *C. albicans*, as shown in the Table 6. Thus, the results obtained with **βF1** and **βF2** show that the inclusion in **βCD** can reveal antimicrobial effects normally hidden by the presence of solvents with some inhibitory activity, such as DMSO.

**Table 6:** Minimal inhibition concentration (μg/mL) of tested compounds included in **βCD**.

	**Concentration (**μ**g/mL)** ± **SE**					
	***Cryptococcus*** **spp**.			***Candida albicans***		
**Compound**	**24 h**	**48 h**	**72 h**	**24 h**	**48 h**	**72 h**
**βF1**	100.0 ± 0.0	200.0 ± 0.0	200.0 ± 0.0	100.0 ± 0.0	122.2 ± 22.2	188.9 ± 11.1
**βF2**	188.9 ± 11.1	NE	NE	200.0 ± 0.0	NE	NE
**βCD**	NE	NE	NE	NE	NE	NE

*SE, Standard error; NE, No effect*.

Interestingly, when we tested **F1**, **F2**, **βF1**, or **βF2** against Gram-negative bacteria (i.e.*, Salmonella enterica*) or Gram-positive bacteria (i.e., *Staphylococcus aureus*), we only observed an effect of **βF1** against *S. aureus* (see Table S7 in the Supplementary Materials). This result suggests that this kind of pyridine Schiff bases could also be useful against bacteria, and the inclusion in **βCD** could be a mean to reveal its activity. Nevertheless, it is necessary more experimentation to elucidate the mechanism of action of these kind Schiff bases against bacteria.

## Conclusion

We synthesized and fully characterized two new pyridine Schiff bases (**F1** and **F2**), and we compared them regarding their antifungal effect against yeasts. In addition, we include **F1** and **F2** into **βCD** in order to improve their solubility in aqueous media. We found that the phenolic ring is crucial for this kind of Schiff bases regarding the antifungal activity, where the positions 4 and 6 are critical. Furthermore, we found that the inclusion in **βCD** improves the solubility in aqueous media and can reveal antimicrobial effects normally hidden by the presence of solvents with some inhibitory activity, such as DMSO.

The study of structural prerequisites for antimicrobial activity, and the inclusion in polymers to improve solubility, is important in the design of new drugs.

## Author contributions

AC: design, synthesis and characterization of Schiff bases, discussion of all the experiments, theoretical calculations and paper writing; LR: contribution in inclusión of Schiff bases into cyclodextrin; DP-H: computational calculations; RM-T: synthesis of cyclodextrin polymer, inclusion of Schiff bases into cyclodextrin; CZ: contribution in UV-vis studies, melting point and TLC; DO: TGA and DSC; MG: electrochemical studies; ES: SBET studies; RA-P: discussion of theoretical calculations; JF: design of Schiff bases, biological assays, discussion of all the experiments, paper writing.

### Conflict of interest statement

The authors declare that the research was conducted in the absence of any commercial or financial relationships that could be construed as a potential conflict of interest.

## Abbreviations

**A**: Pore size
**ATR**: Attenuated total reflection
**B3LYP**: calculations based on Becke–Lee–Yang–Parr
**βCD**: Epichlorohydrin-β-cyclodextrine polymer
**βF1**: **F1** included into **βCD**
**βF2**: **F2** included into **βCD**
**COSMO**: COnductor-like Screening MOdel
**δ**: proton chemical shift used in ^1^HNMR spectroscopy
**DEPT**: Distortionless Enhancement by Polarization Transfer
**DFT**: density functional theory
**DSC**: Differential scanning calorimetry
**ε**: relative static permittivity
**E^*ox*^**: Oxidation potential measured in volts under standard conditions
**E^red^**: Reduction potential measured in volts under standard conditions
**F1**: (*E*)-2-[(3-Amino-pyridin-4-ylimino)-methyl]-4,6-difluoro-phenol
**F2**: (*E*)- 2-[(3-Amino-pyridin-4-ylimino)-methyl]-6-fluoro-phenol
**Hz**: Hertz
**HOMO**: highest occupied molecular orbital
**IHB**: Intramolecular Hydrogen Bond
**J**: coupling constant used in ^1^HNMR spectroscopy
***K_i_***: Apparent inclusion constant
**LUMO**: lowest unoccupied molecular orbital
**m/z**: mass divided by charge used in mass spectroscopy
**MIC**: Minimal inhibitory concentration
**NBO**: natural bond orbital
**NMR**: Nuclear magnetic resonance
**ppm**: parts per million
**Salen**: abbreviation for a popular chelating ligand formed by salicylaldehyde and ethylenediamine
**S_BET_**: Brunauer–Emmett–Teller Surface area
**TD-DFT**: Time-dependent density functional theory
**TDT**: Thermal decomposition temperature
**Tg**: Glass transition temperature
**TGA**: Thermogravimetric analysis
**TOCSY**: TOtal Correlated SpectroscopY.
