# Supplementary material for: Two New Fluorinated Phenol Derivatives Pyridine Schiff Bases: Synthesis, Spectral, Theoretical Characterization, Inclusion in Epichlorohydrin-β-Cyclodextrin Polymer, and Antifungal Effect
Source: Front Chem. 2018 Jul 30;6:312. doi: 10.3389/fchem.2018.00312 (PMC6080543; doi:10.3389/fchem.2018.00312)
Supplement: Supplementary file 1 [file Table_1.docx]

**Supplementary Tables**

**Table S1**. Characteristic constants of fluorinated pyridine Schiff bases **F1** and **F2**.

| **Schiff Base** | **Molecular Weight (g mol^-1^)** | **Yield (%)** | **Melting point (ºC)** | **Solid Color** | **Thin Layer Chromatography R_f_** |
| --- | --- | --- | --- | --- | --- |
| **F1** | 249.0 | 78 | 182.5 – 183.7 | Yellow | 0.31 |
| **F2** | 231.0 | 82 | 163.4 – 164.8 | Yellow | 0.40 |

**Table S2.** UV-Vis absorption spectra of **F1** and **F2**.

| **Compound** | **Dichloromethane** | | **Acetonitrile** | | **DMSO** | |
| --- | --- | --- | --- | --- | --- | --- |
|  | **λ exp*** | **ε** | **λ exp** | **ε** | **λ exp** | **ε** |
| **F1** | 366  260 (sh 278) | 6.29  11.59 | 362 | 7.76 | 374  262 | 7.70  14.96 |
| **F2** | 364  274 | 9.46  10.18 | 360 | 9.30 | 372  262 | 9.90  15.80 |

* λ in nm

ε (10^3^ mol^-1^ dm^3^ cm^-1^)

**Table S3.** Optimized geometrical parameters (All the distances are in Å).

| **Compound** | **d(N-C_py_)** | **d(C-N)**  **azomethine group** | **d(N-H)** | **d(O-H)**  **phenolic ring** |
| --- | --- | --- | --- | --- |
| **F1** | 1.367 | 1.284 | 1.772 | 0.989 |
| **F2** | 1.368 | 1.285 | 1.773 | 0.988 |

**Table S4.** Frequencies of the principal groups calculated for **F1** and **F2**.

| **Compound** | **νOH (cm^-1^)** | **νNH (cm^-1^)** | **νN=C (cm^-1^)** |
| --- | --- | --- | --- |
| **F1** | 3252 | ν_as_: 3702  ν_s_: 3595 | 1626 |
| **F2** | 3252 | ν_as_: 3701  ν_s_: 3593 | 1592 |

**Table S5.** Most important transition energies calculated for **F1** and **F2**

| **Compound** | **Solvent** | **λ(nm)** | **f** | **Assignment** |
| --- | --- | --- | --- | --- |
| **F1** | CH_2_Cl_2_ | 216 | 0.32 | HOMO-1 → LUMO+4 (n→π*)  HOMO-2 → LUMO+1 (n→π*) |
|  |  | 288 | 0.29 | HOMO-3 → LUMO (n→π*)  HOMO-2 → LUMO (n→π*) |
|  |  | 390 | 0.47 | HOMO → LUMO (π→π*) |
|  | ACN | 214 | 0.28 | HOMO-1 → LUMO+4 (n→π*)  HOMO-2 → LUMO+1 (n→π*) |
|  |  | 289 | 0.26 | HOMO-3 → LUMO (n→π*)  HOMO-2 → LUMO (n→π*) |
|  |  | 385 | 0.30 | HOMO → LUMO (π→π*) |
|  | DMSO | 220 | 0.38 | HOMO-1 → LUMO+4 (n→π*)  HOMO-2 → LUMO+1 (n→π*) |
|  |  | 284 | 0.31 | HOMO-3 → LUMO (n→π*)  HOMO-2 → LUMO (n→π*) |
|  |  | 390 | 0.30 | HOMO → LUMO (π→π*) |
|  | Gas phase | 213 | 0.28 | HOMO-1 → LUMO+3 (n→π*)  HOMO-2 → LUMO+1 (n→π*) |
|  |  | 277 | 0.24 | HOMO-3 → LUMO (n→π*)  HOMO-2 → LUMO (n→π*) |
|  |  | 394 | 0.33 | HOMO → LUMO (π→π*) |
| **F2** | CH_2_Cl_2_ | 220 | 0.30 | HOMO-2 → LUMO+3 (n→π*)  HOMO-2 → LUMO+1 (n→π*) |
|  |  | 290 | 0.32 | HOMO-3 → LUMO (n→π*)  HOMO-2 → LUMO (n→π*) |
|  |  | 380 | 0.56 | HOMO → LUMO (π→π*) |
|  | ACN | 215 | 0.40 | HOMO-1 → LUMO+3 (n→π*)  HOMO-2 → LUMO+1 (n→π*) |
|  |  | 283 | 0.30 | HOMO-3 → LUMO (n→π*)  HOMO-2 → LUMO (n→π*) |
|  |  | 387 | 0.56 | HOMO → LUMO (π→π*) |
|  | DMSO | 218 | 0.40 | HOMO-1 → LUMO+3 (n→π*)  HOMO-2 → LUMO+1 (n→π*) |
|  |  | 285 | 0.32 | HOMO-3 → LUMO (n→π*)  HOMO-2 → LUMO (n→π*) |
|  |  | 382 | 0.56 | HOMO → LUMO (π→π*) |
|  | Gas phase | 216 | 0.22 | HOMO-1 → LUMO+3 (n→π*)  HOMO-2 → LUMO+1 (n→π*) |
|  |  | 284 | 0.27 | HOMO-3 → LUMO (n→π*)  HOMO-2 → LUMO (n→π*) |
|  |  | 389 | 0.47 | HOMO → LUMO (π→π*) |

**Table S6.** Scan rate study results

| Compound | | **F1** | | **F2** | |
| --- | --- | --- | --- | --- | --- |
| Process | | E_ox_ | E_red_ | E_ox_ | E_red_ |
| V vs SCE | | +1.07 | -0.89 | +0.92 | -1.31 |
| Scan-rate vs current-density peak | m | 6.0×10^7^ | -3.0×10^7^ | 5.0×10^6^ | -2.0×10^6^ |
|  | n | -96.3 | -131.2 | 1.4 | -7.0 |
|  | R^2^ | 0.914 | 0.968 | 0.990 | 0.882 |
| (Scan-rate)^½^ vs current-density peak | m | 3.0×10^6^ | -2.0×10^6^ | 9.0×10^7^ | -5.0×10^7^ |
|  | n | -1.0 | -2.6 | -56.0 | -201.5 |
|  | R^2^ | 0.973 | 0.996 | 0.997 | 0.786 |
| Diffusion control? | | yes | yes | no | yes |

**Table S7.** Minimal inhibition concentration (μg/mL) of tested compounds alone or included in **βCD**

|  | **Concentration (μg/mL) ± SE** | |
| --- | --- | --- |
| **Compound** | ***Salmonella enterica***  **(18 h)** | ***Staphylococcus aureus***  **(18 h)** |
| **F1** | - | - |
| **F2** | - | - |
| **βF1** | NE | 200.0 ± 0.0 |
| **βF2** | NE | NE |
| **βCD** | NE | NE |

SE: Standard error

- : Undistinguishable from DMSO alone (i.e. no effect)

NE: No effect
