# Supplementary material for: Two New Fluorinated Phenol Derivatives Pyridine Schiff Bases: Synthesis, Spectral, Theoretical Characterization, Inclusion in Epichlorohydrin-β-Cyclodextrin Polymer, and Antifungal Effect
Source: Front Chem. 2018 Jul 30;6:312. doi: 10.3389/fchem.2018.00312 (PMC6080543; doi:10.3389/fchem.2018.00312)
Supplement: Supplementary file 2 [file Presentation_1.pptx]

## Slide 1
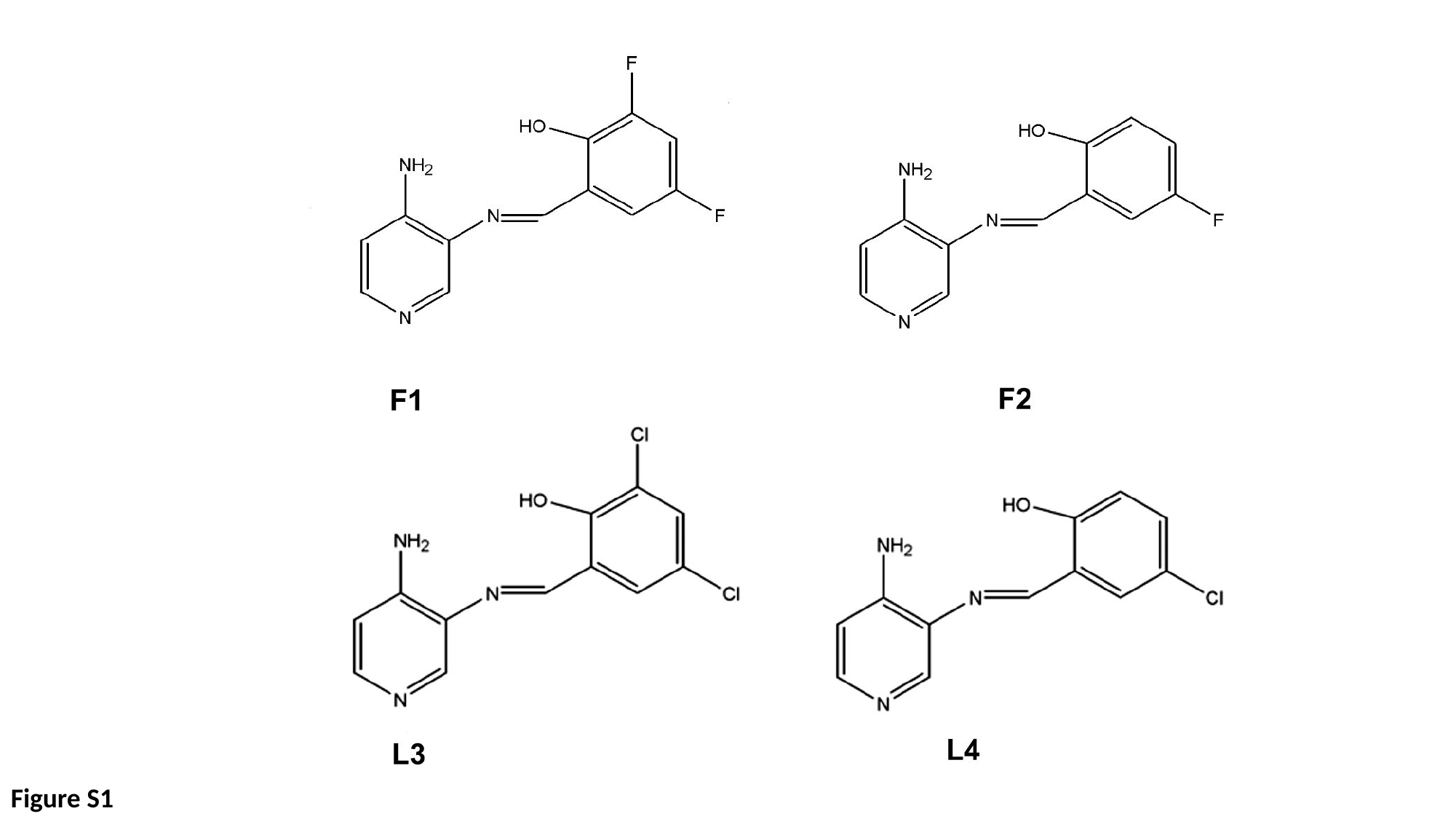

Figure S1

## Slide 2
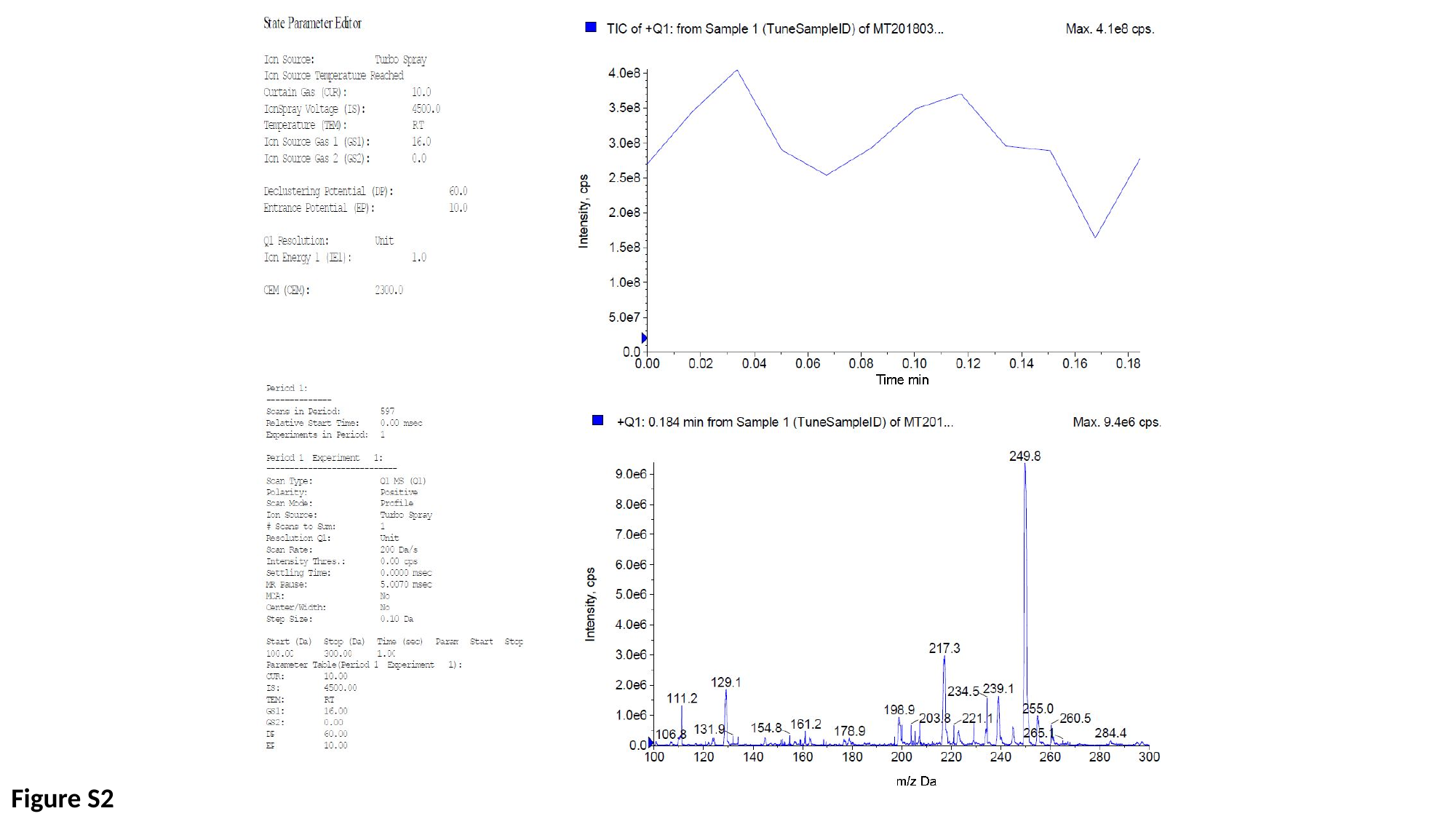

Figure S2

## Slide 3
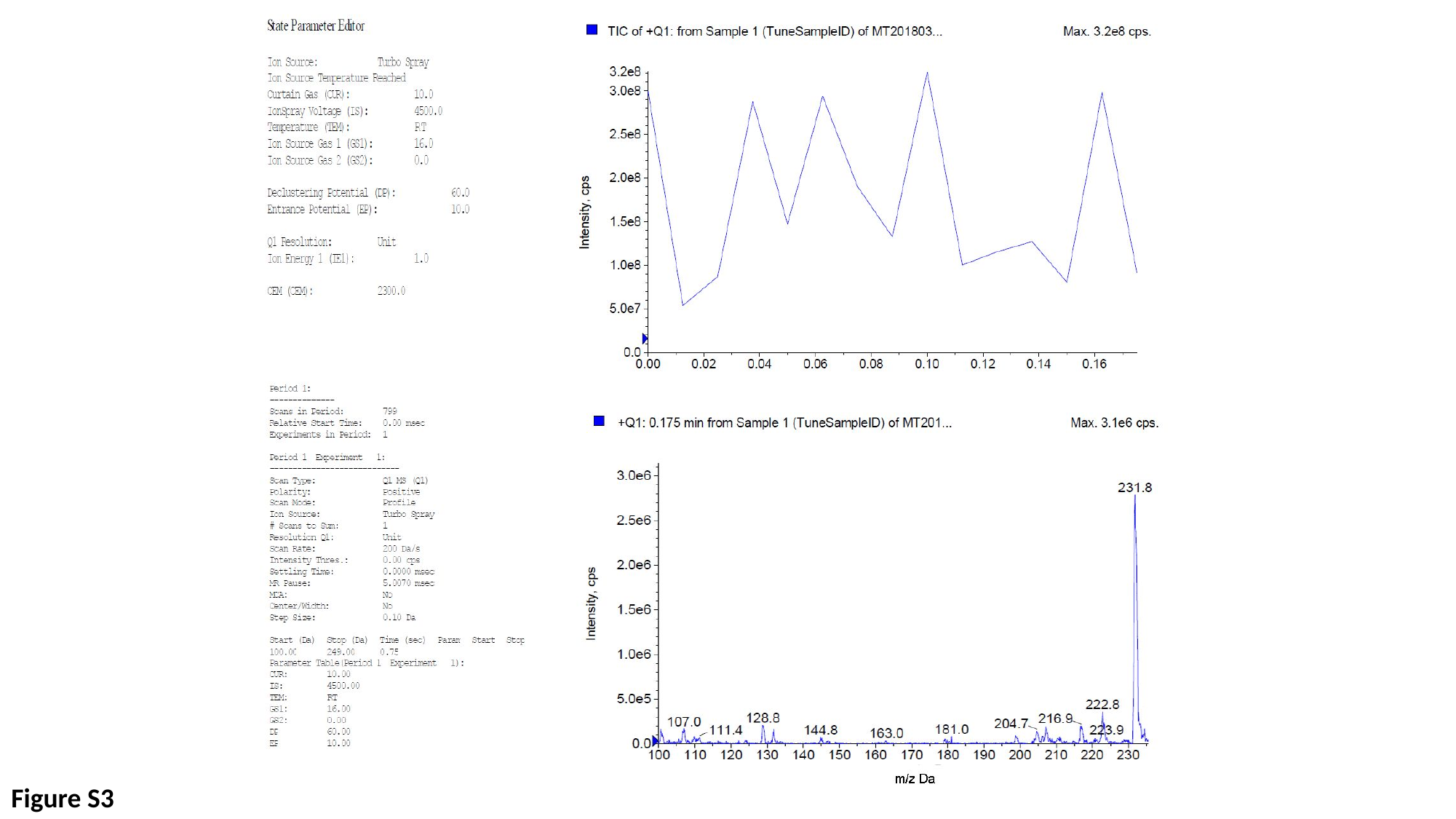

Figure S3

## Slide 4
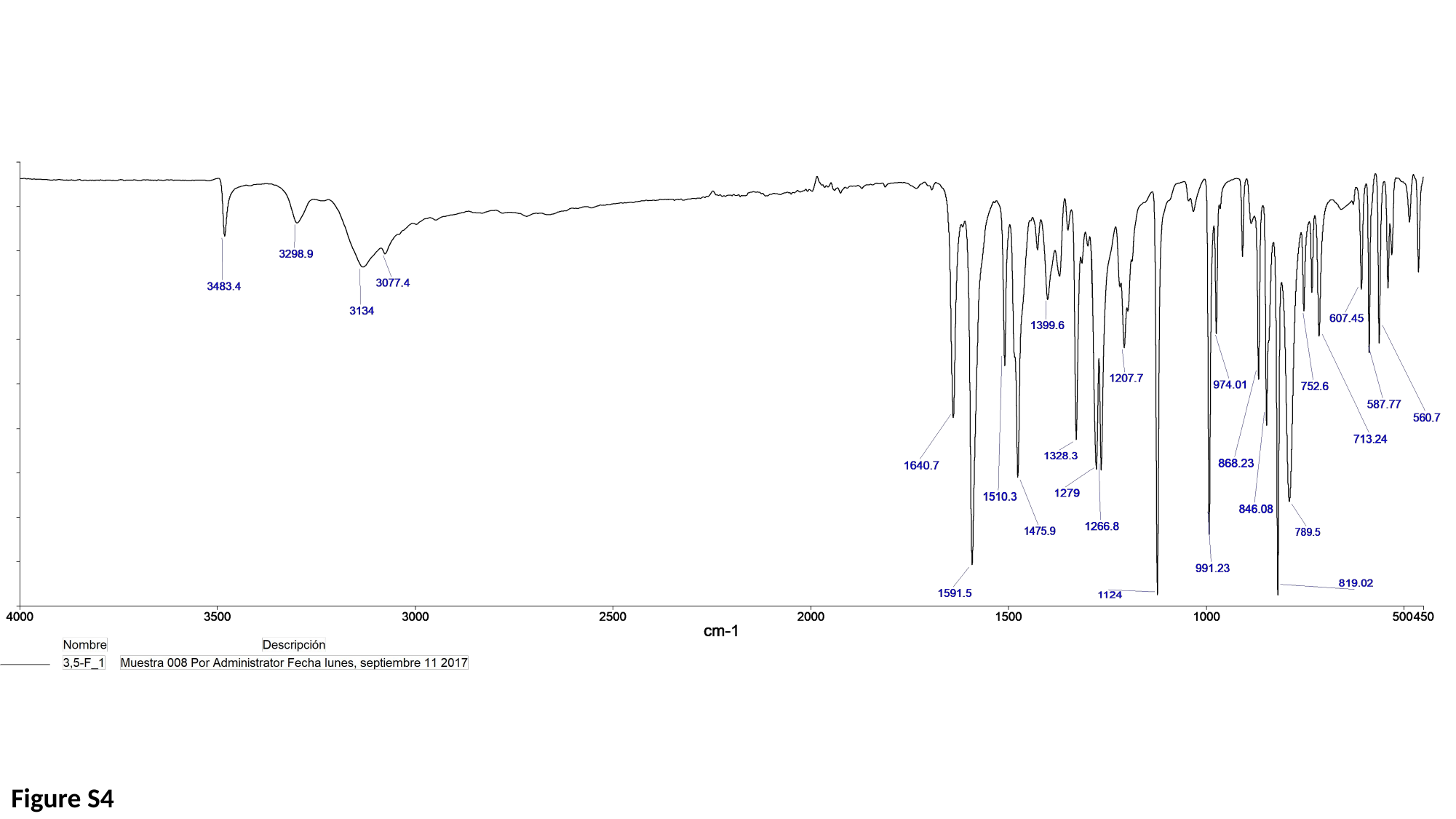

Figure S4

## Slide 5
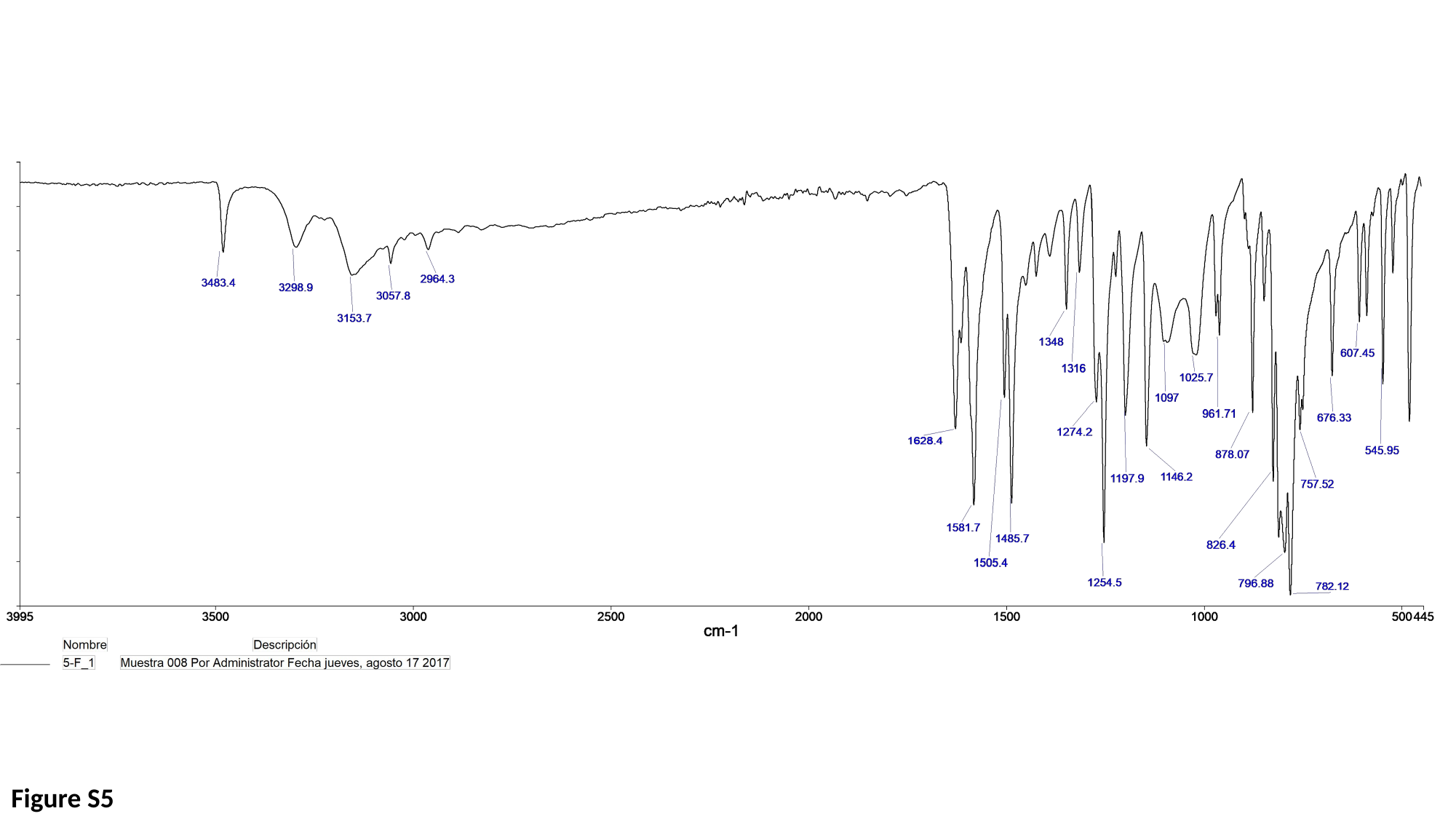

Figure S5

## Slide 6
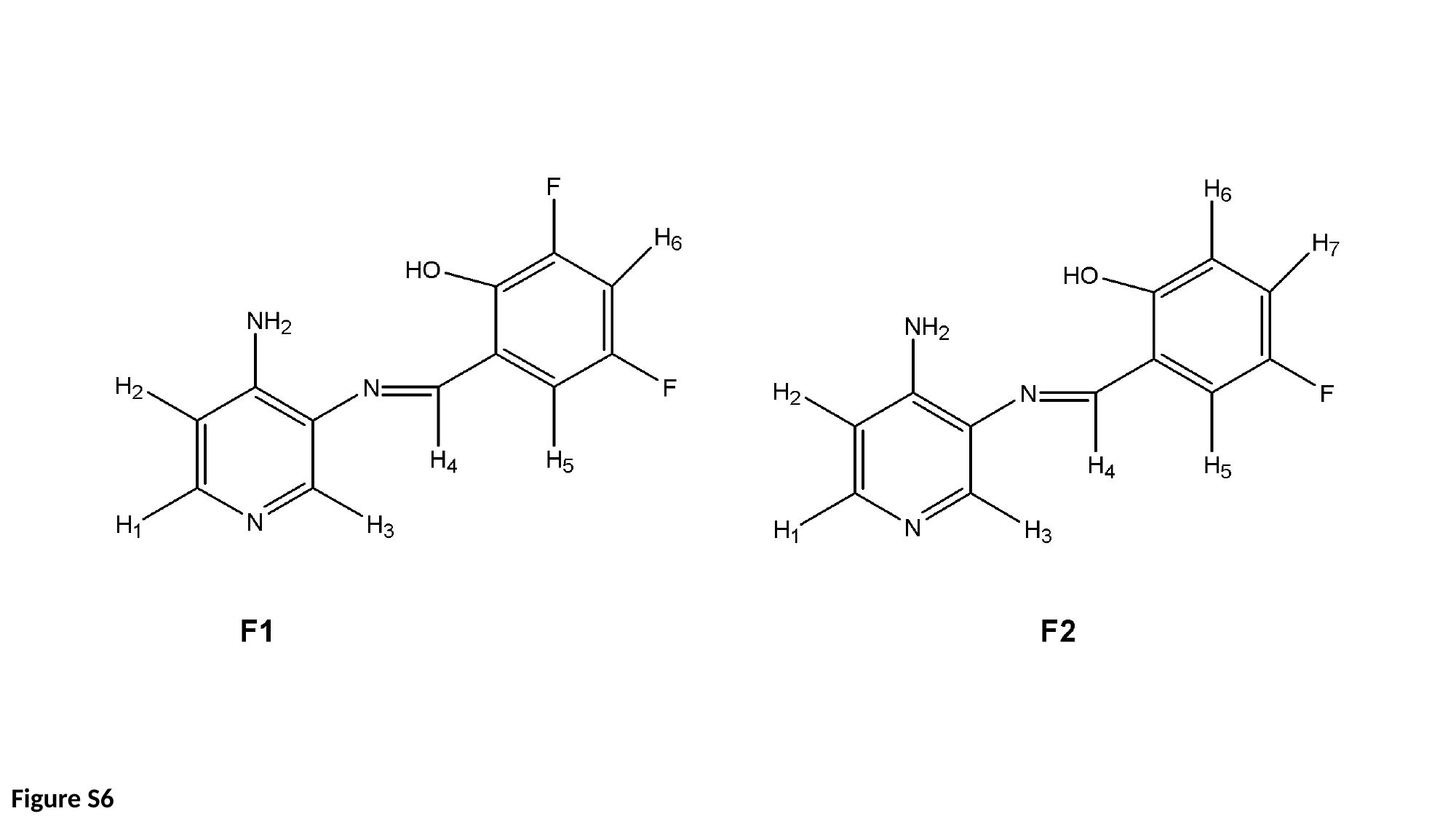

Figure S6

## Slide 7
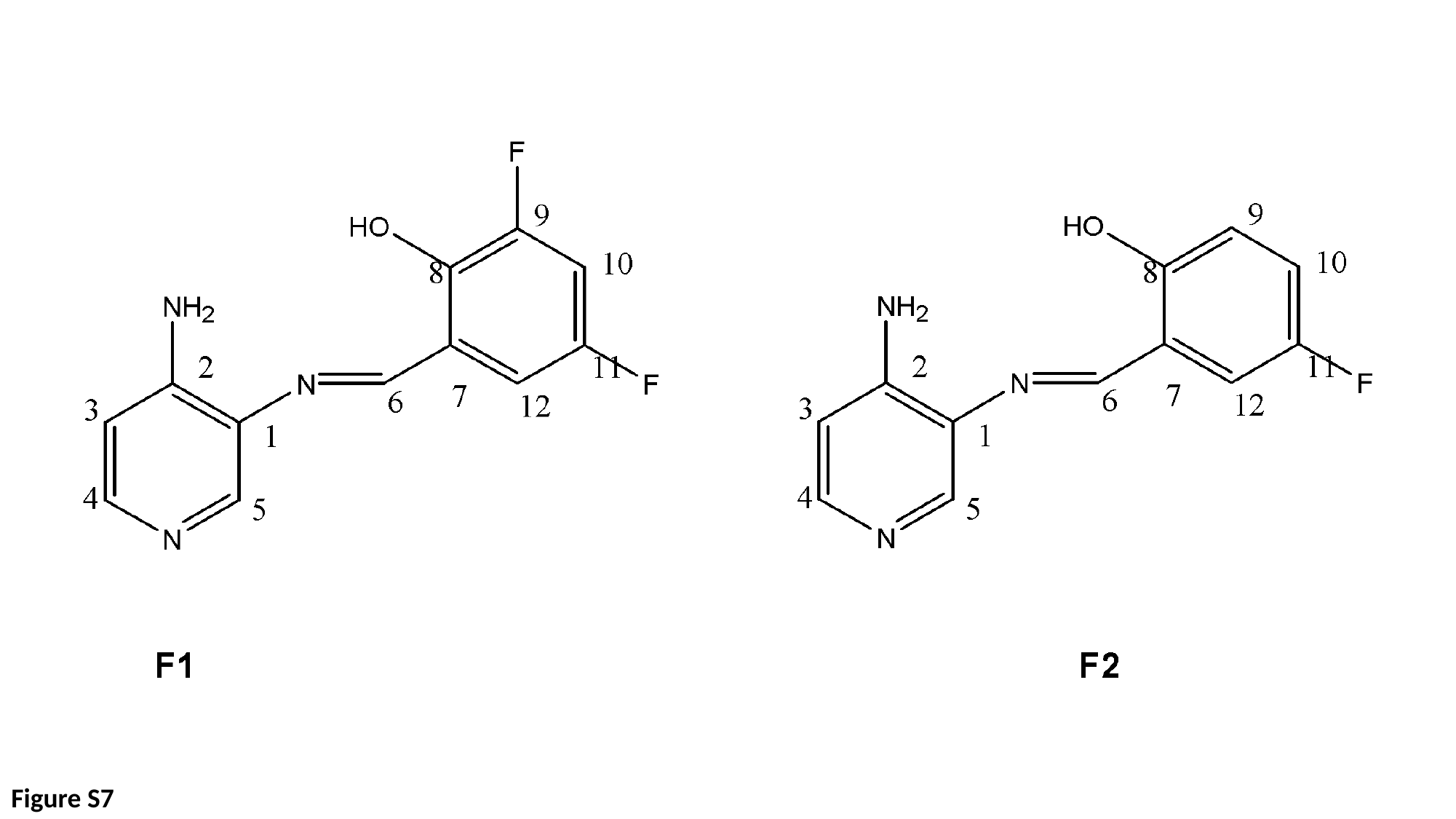

Figure S7

## Slide 8
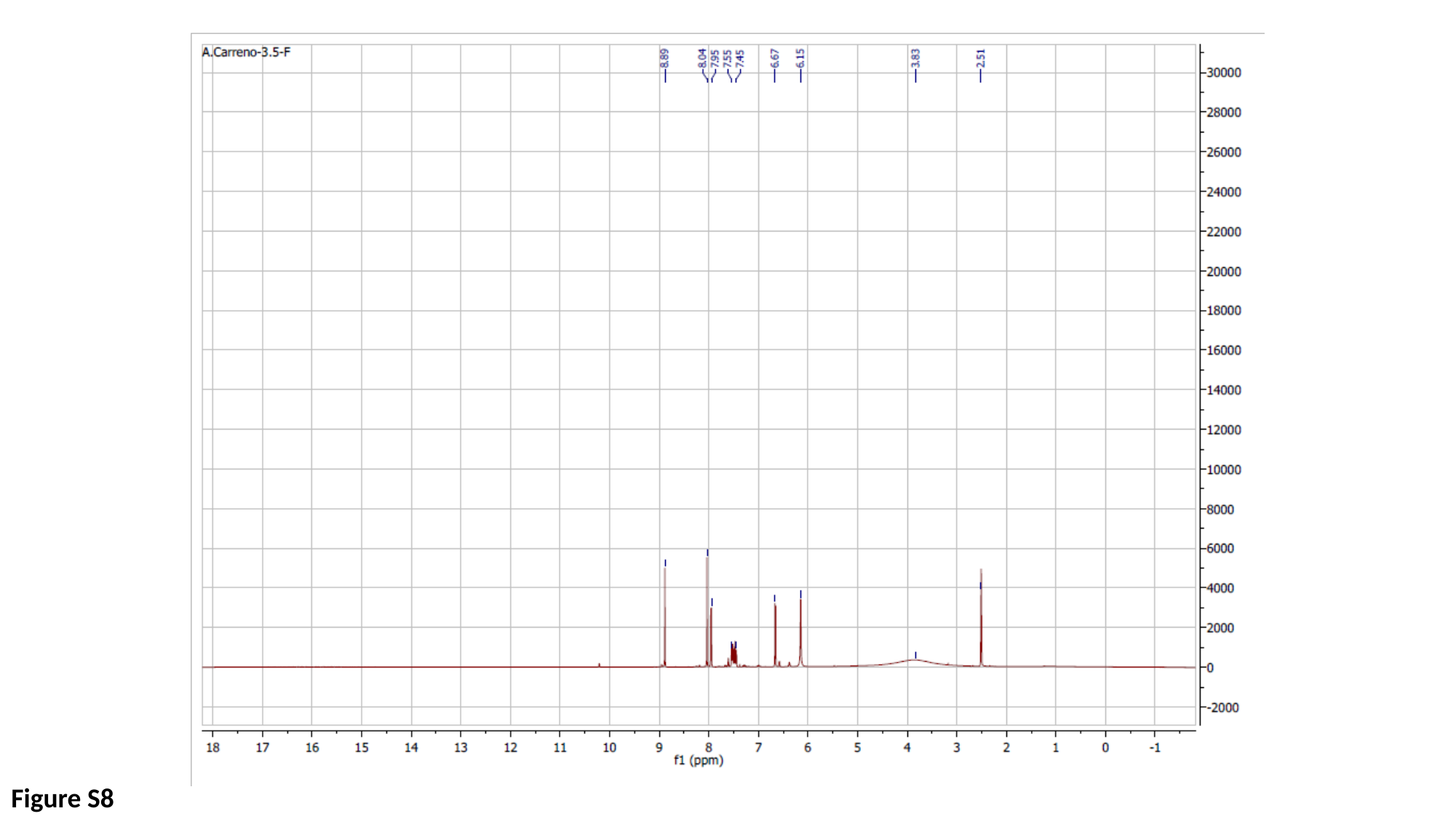

Figure S8

## Slide 9
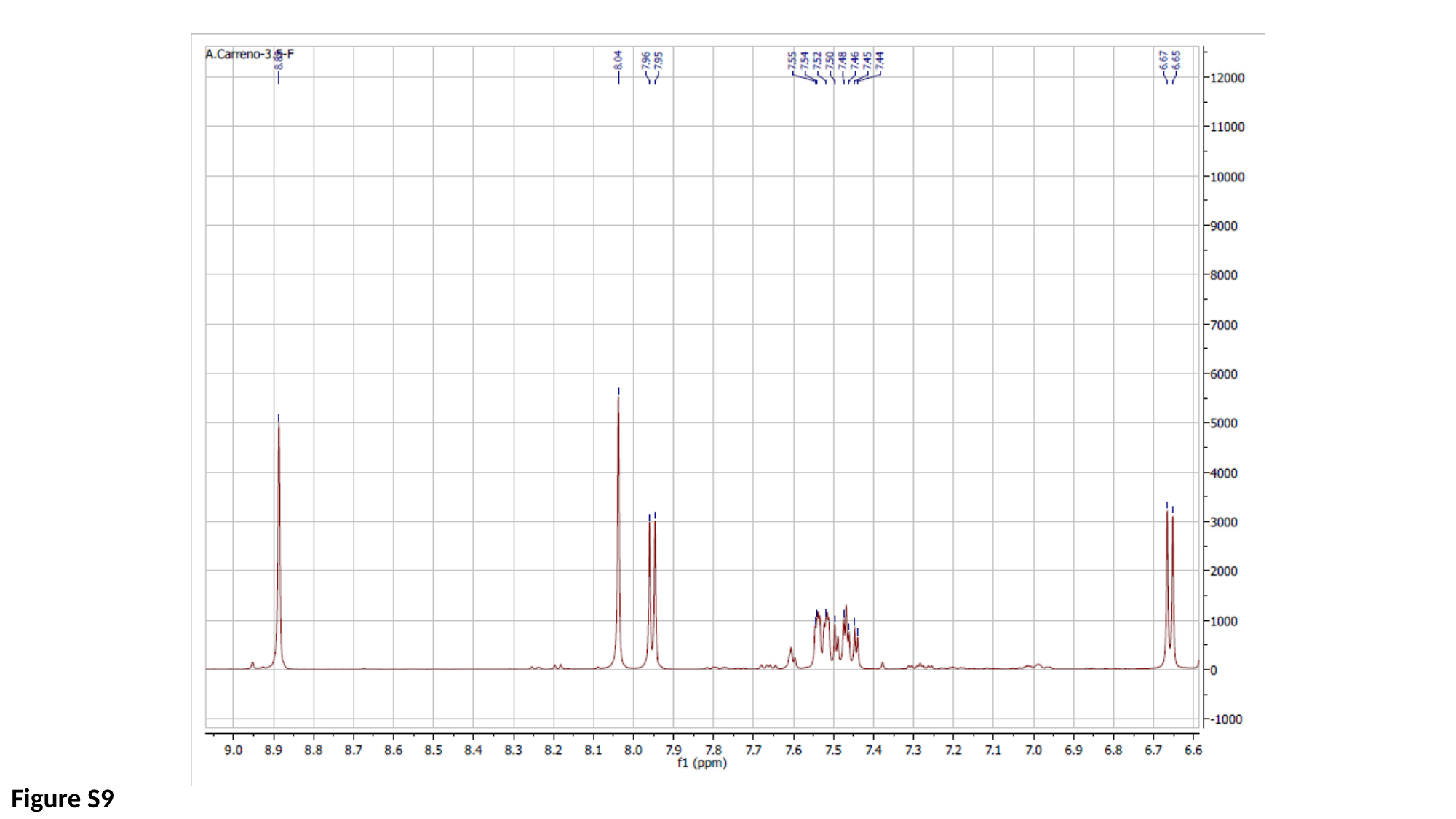

Figure S9

## Slide 10
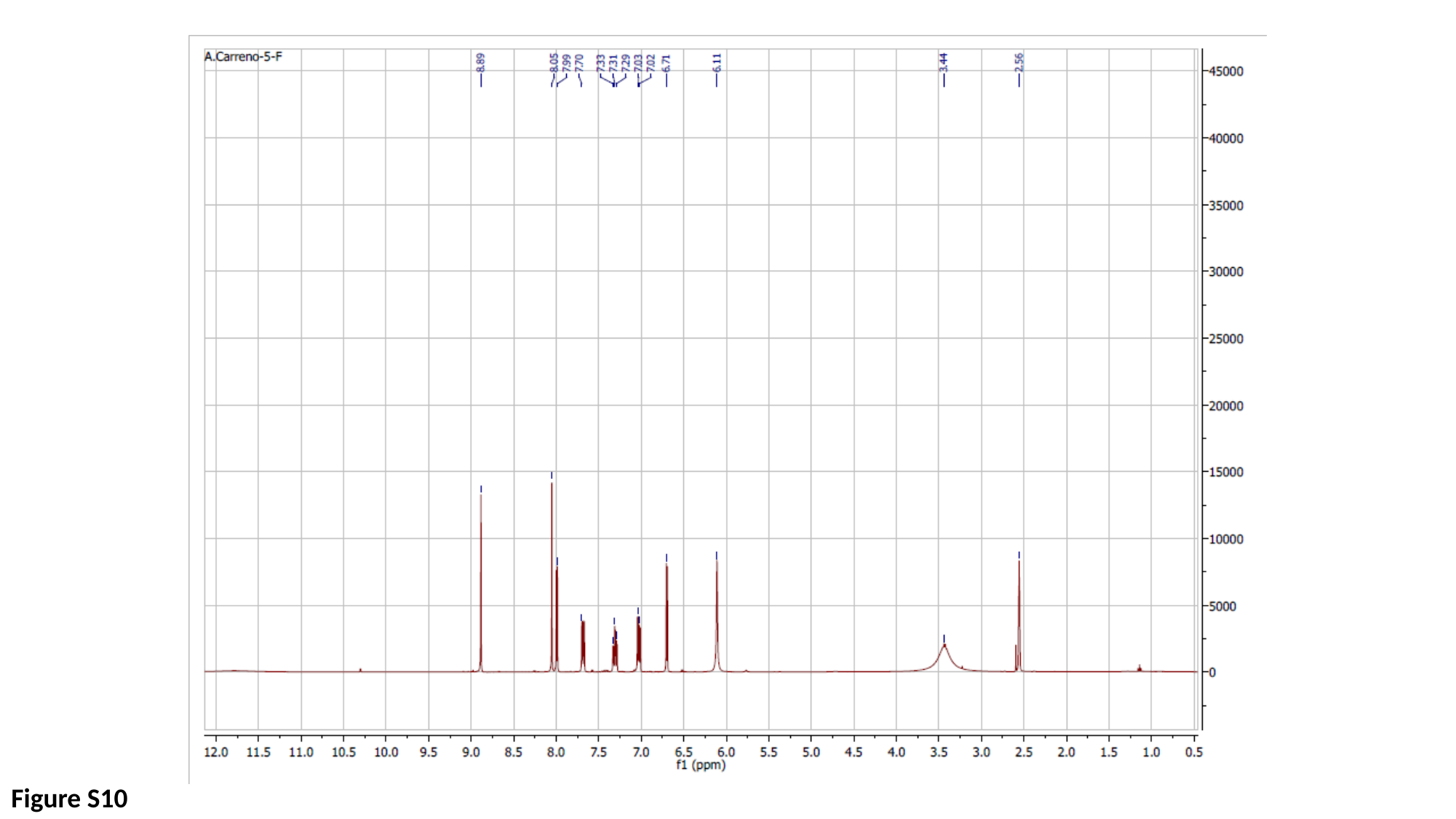

Figure S10

## Slide 11
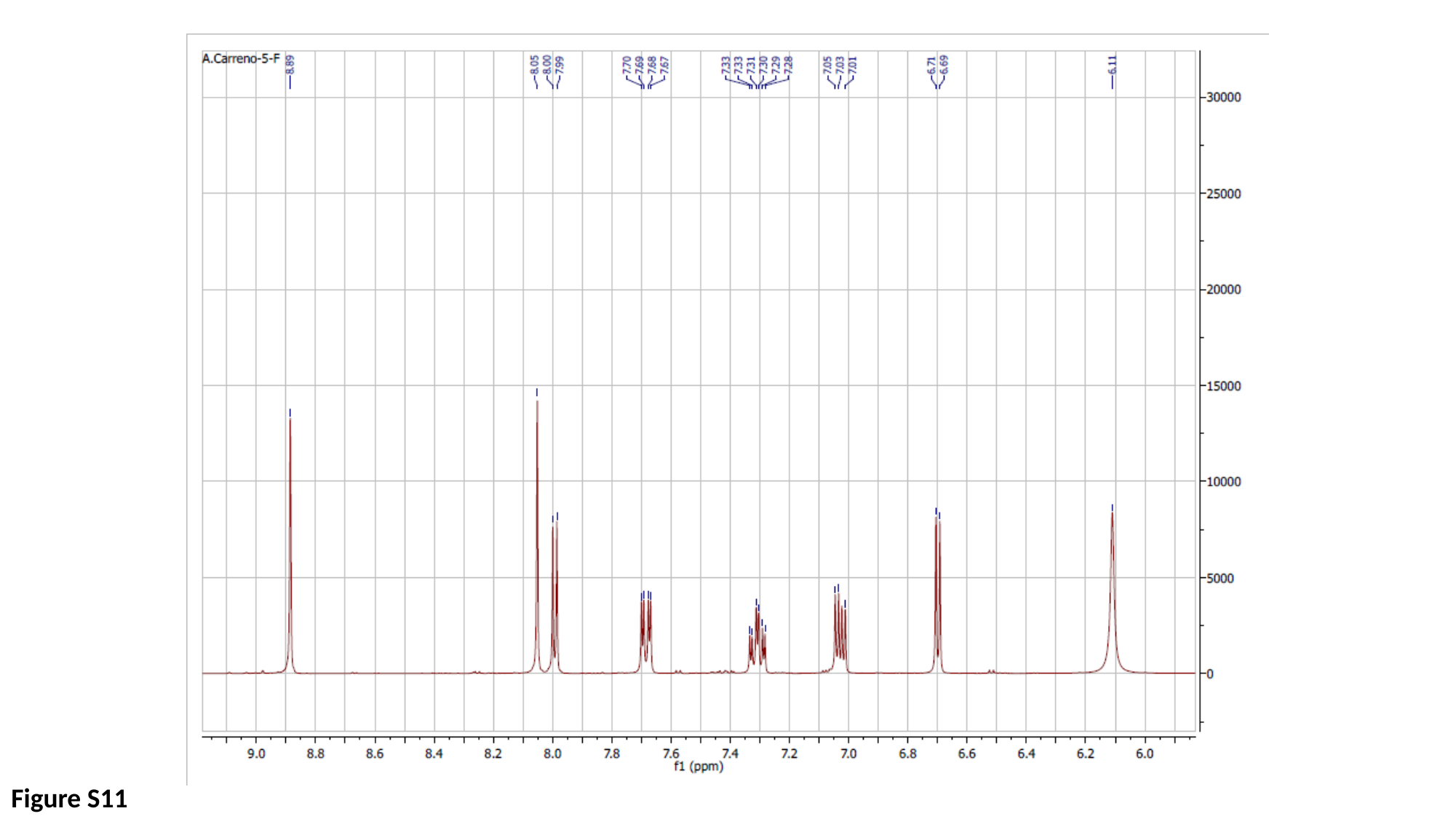

Figure S11

## Slide 12
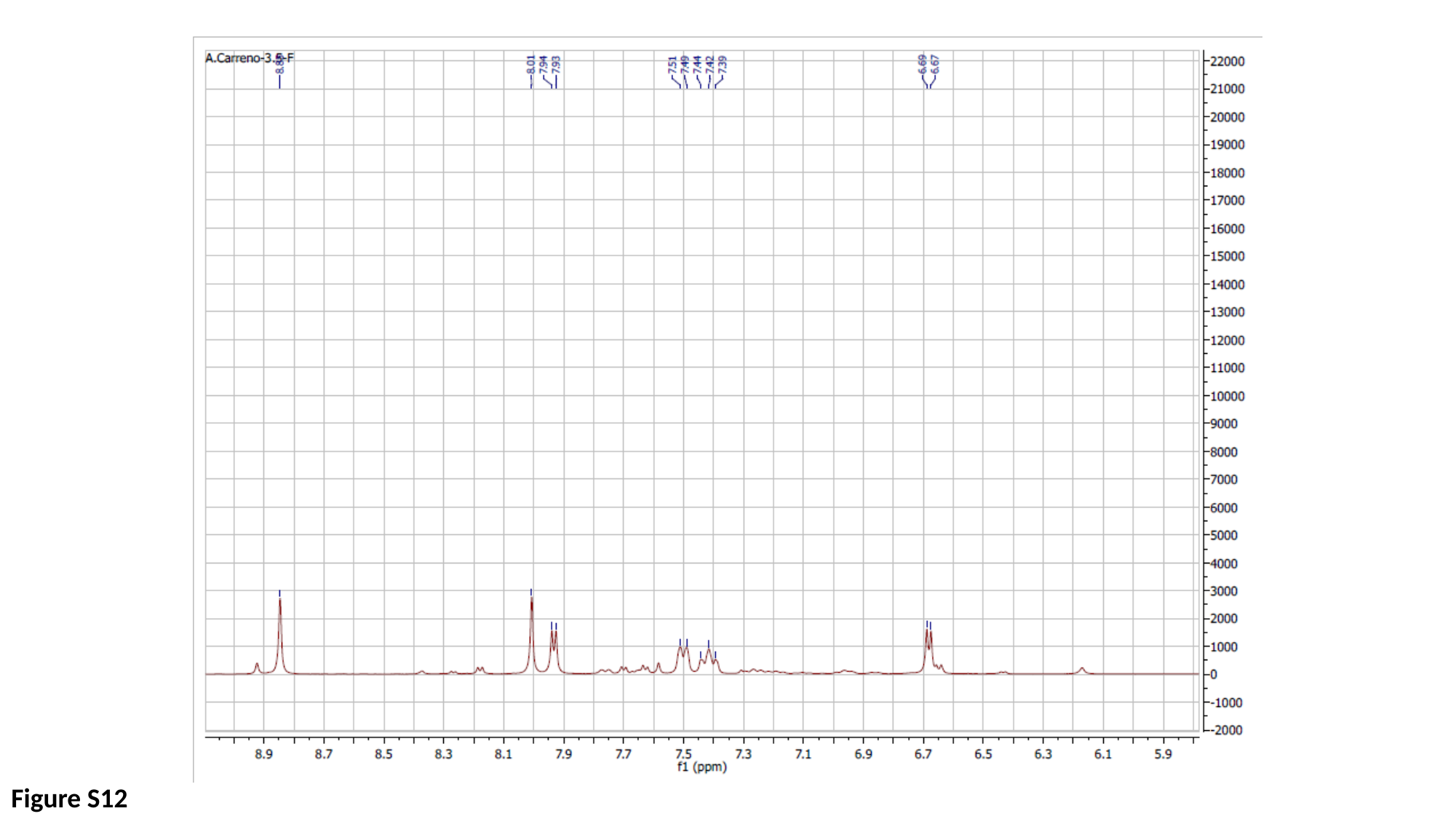

Figure S12

## Slide 13
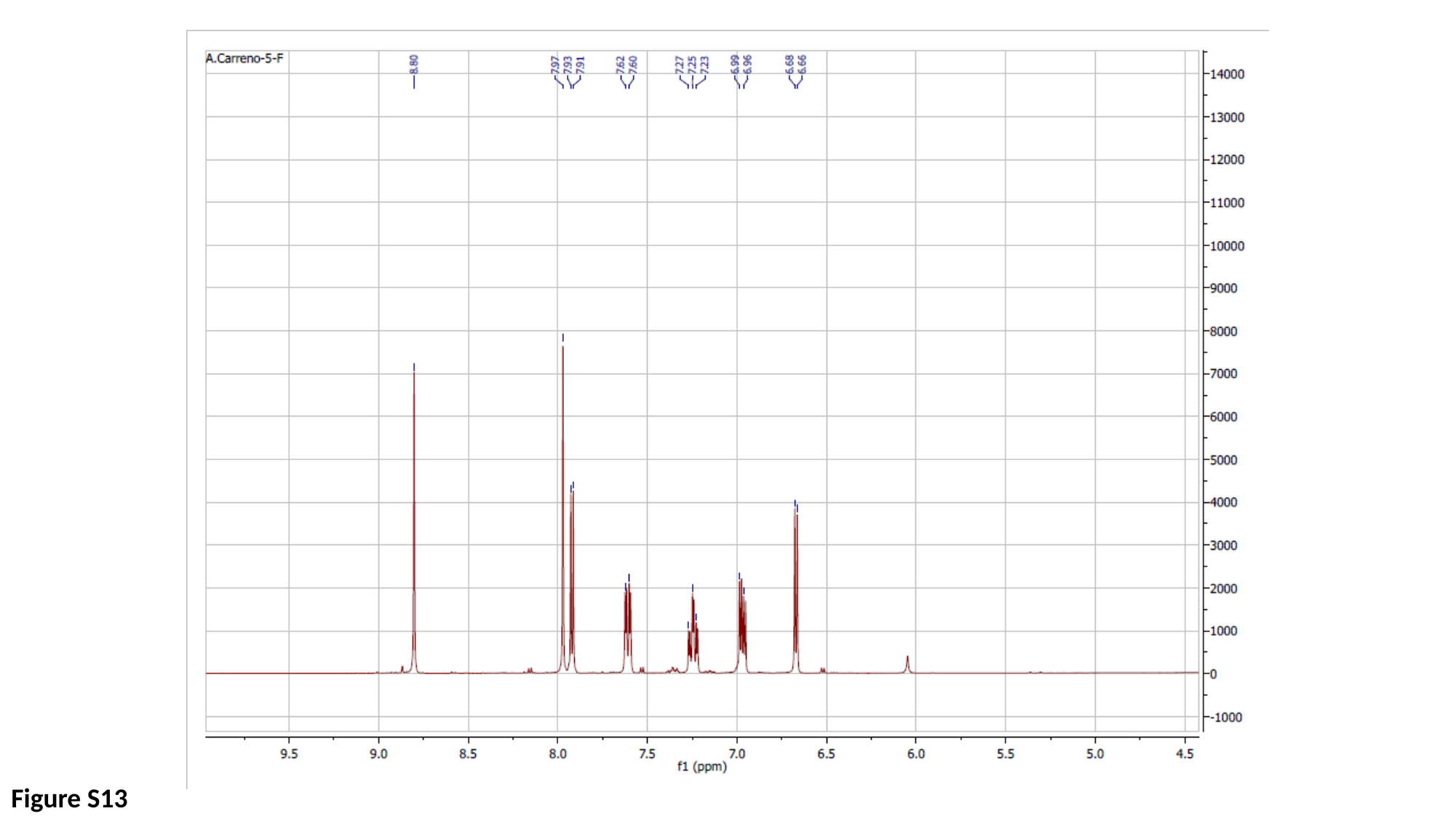

Figure S13

## Slide 14
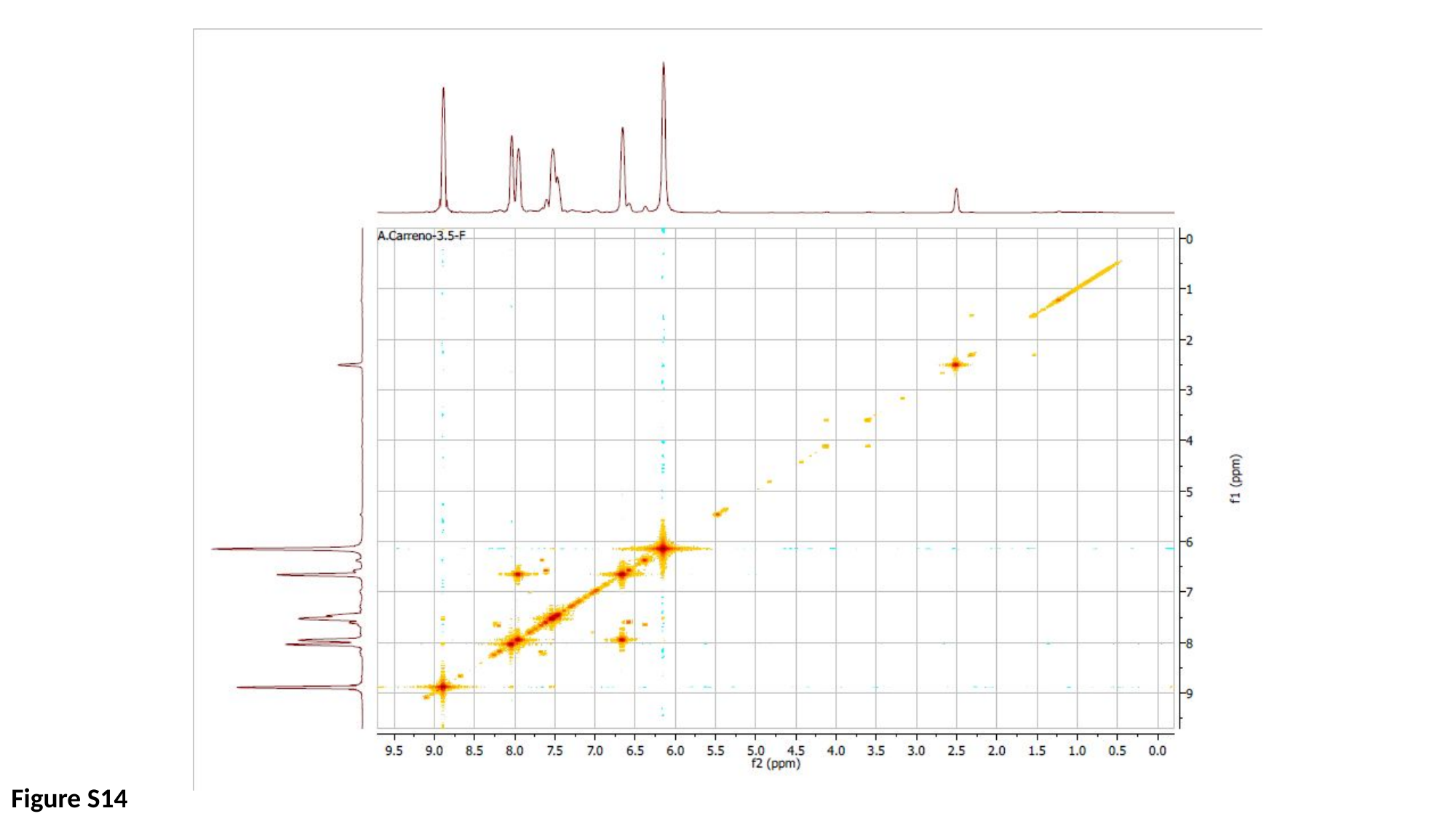

Figure S14

## Slide 15
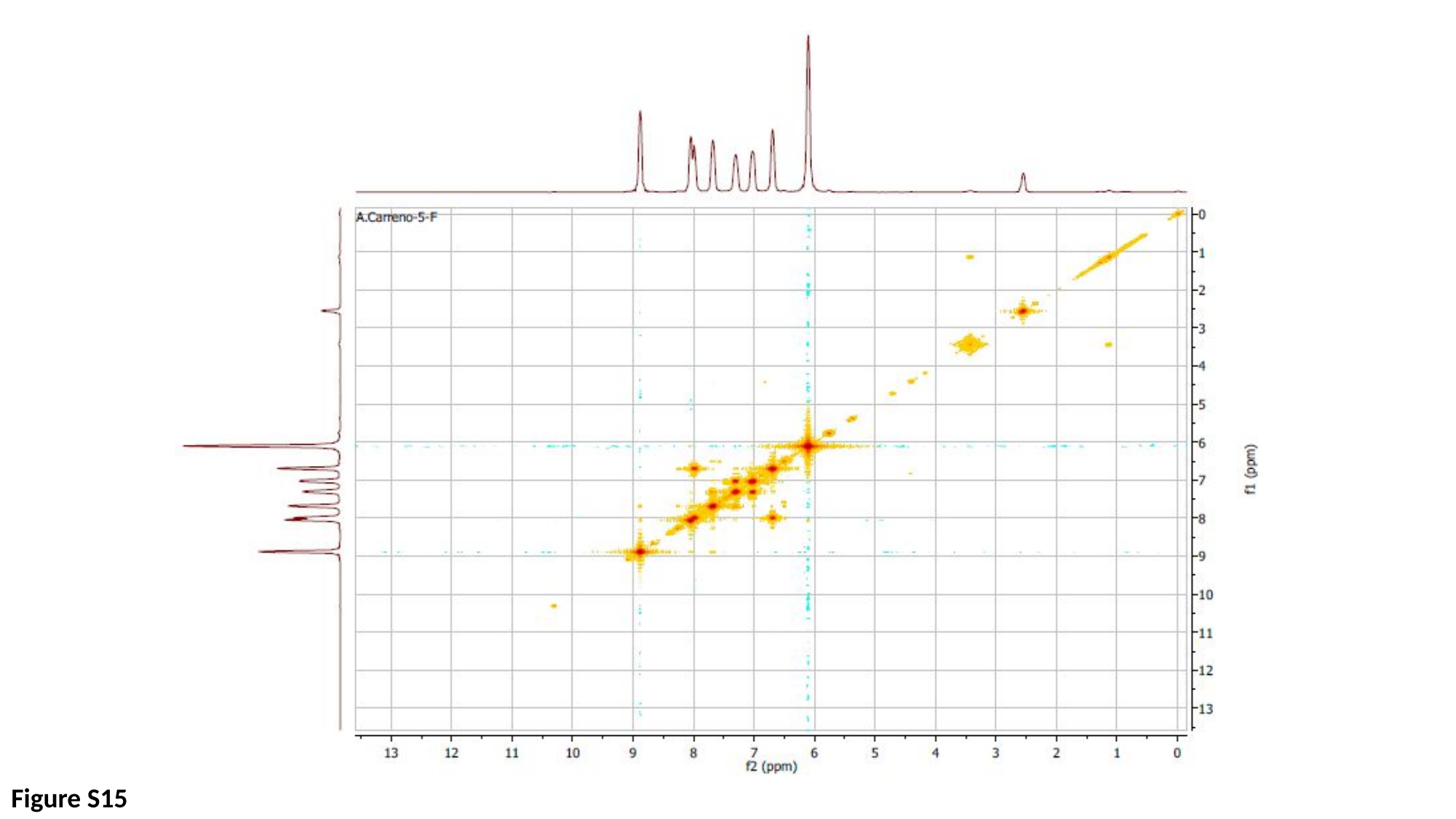

Figure S15

## Slide 16
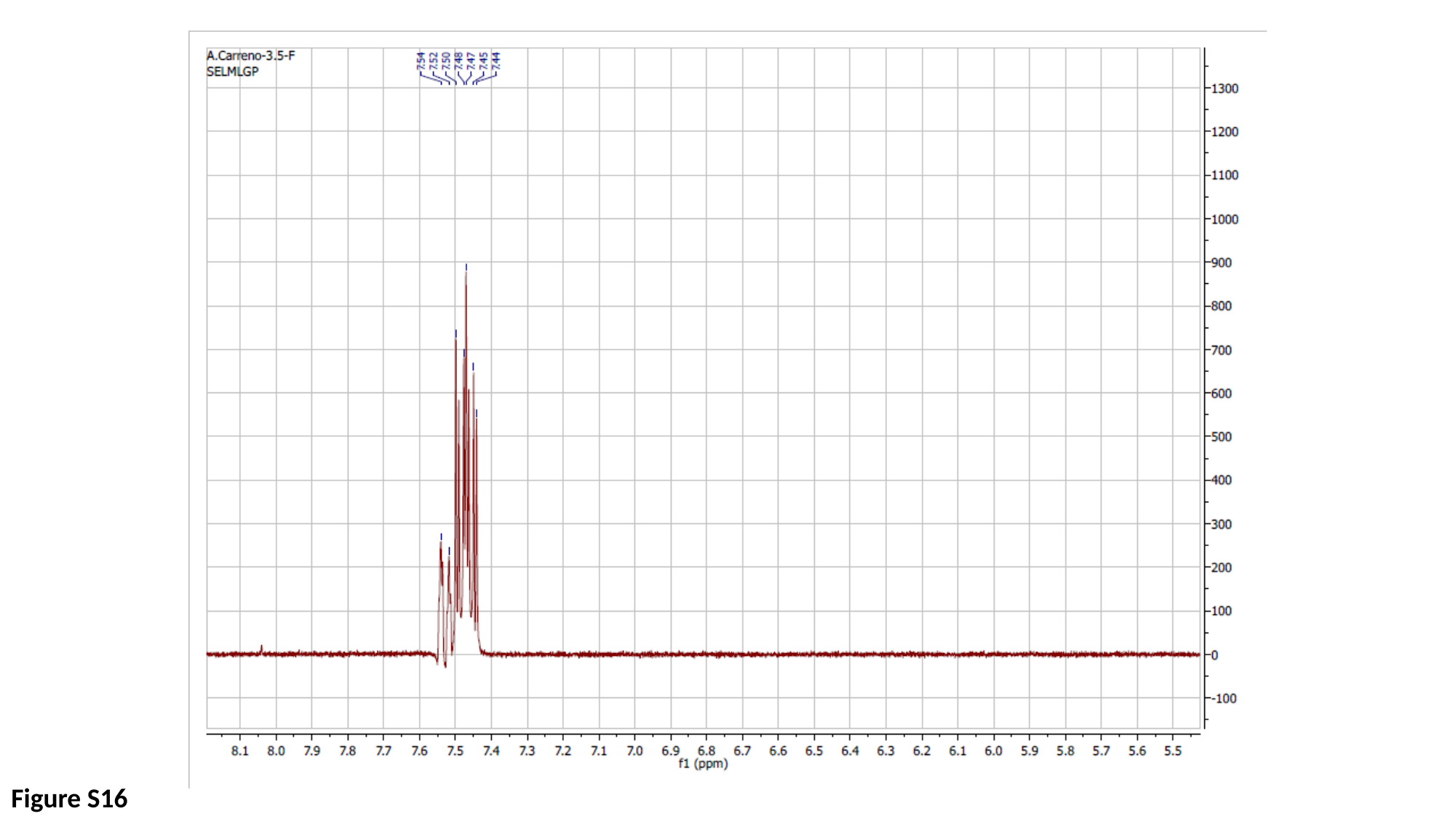

Figure S16

## Slide 17
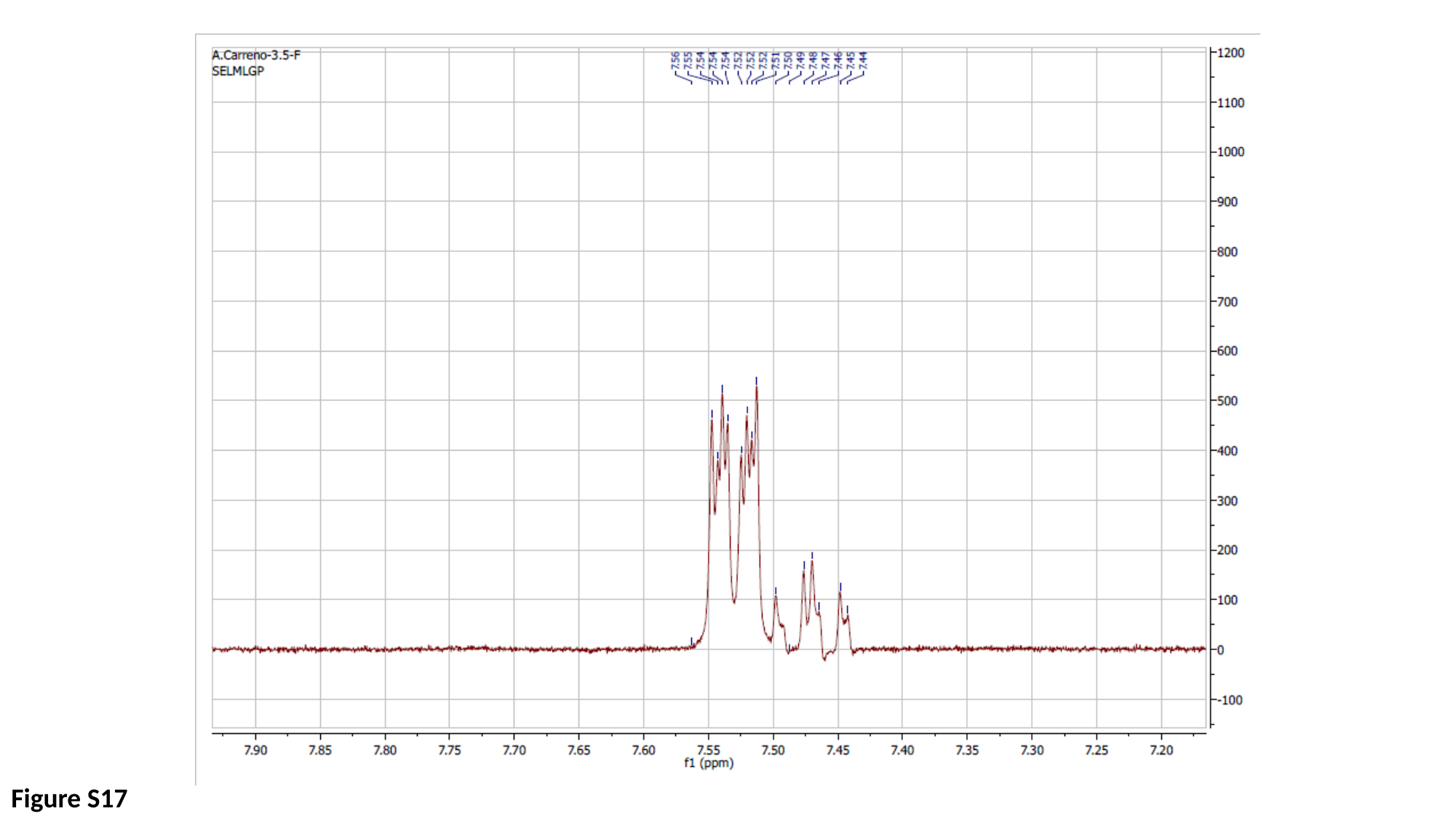

Figure S17

## Slide 18
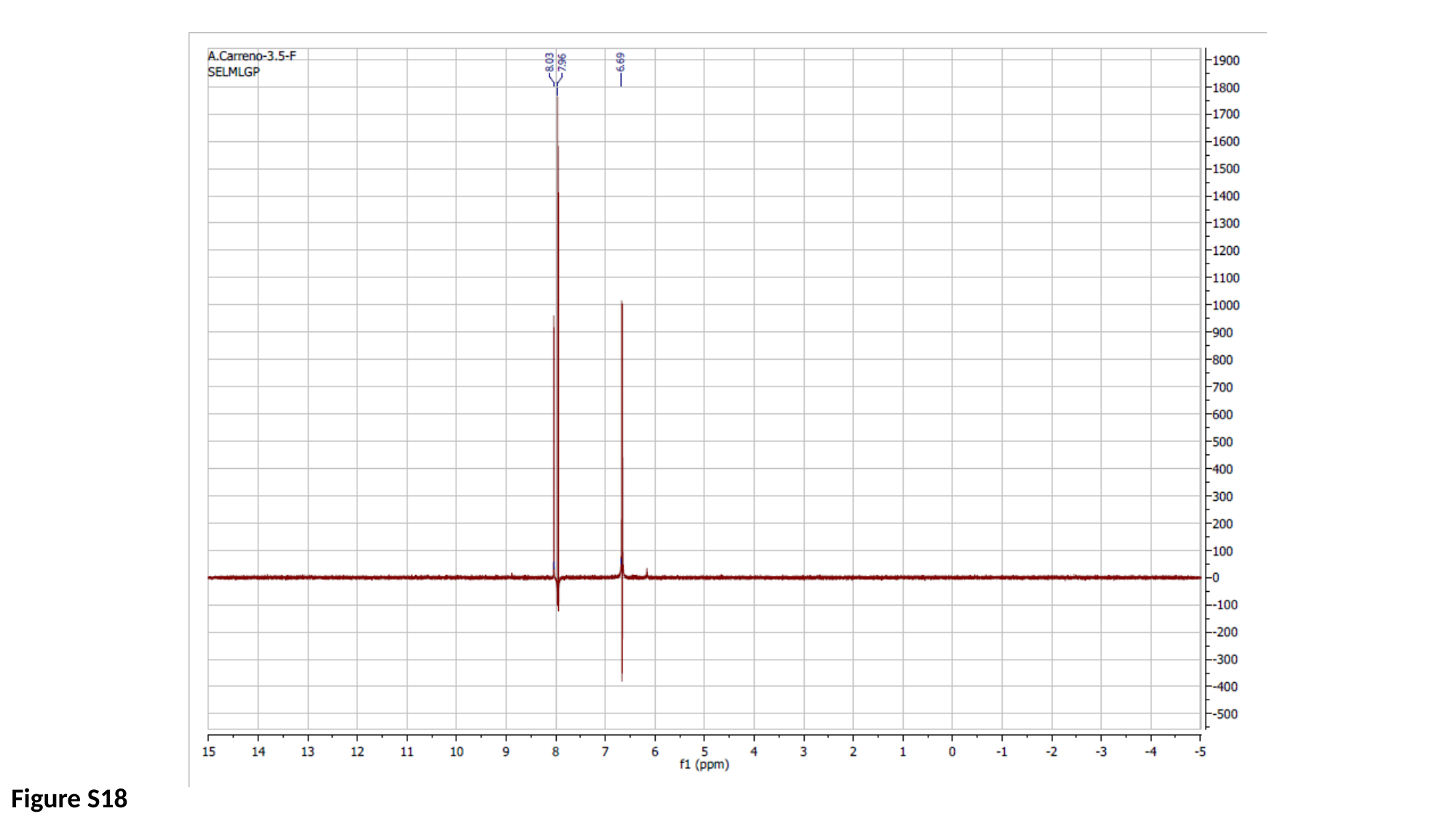

Figure S18

## Slide 19
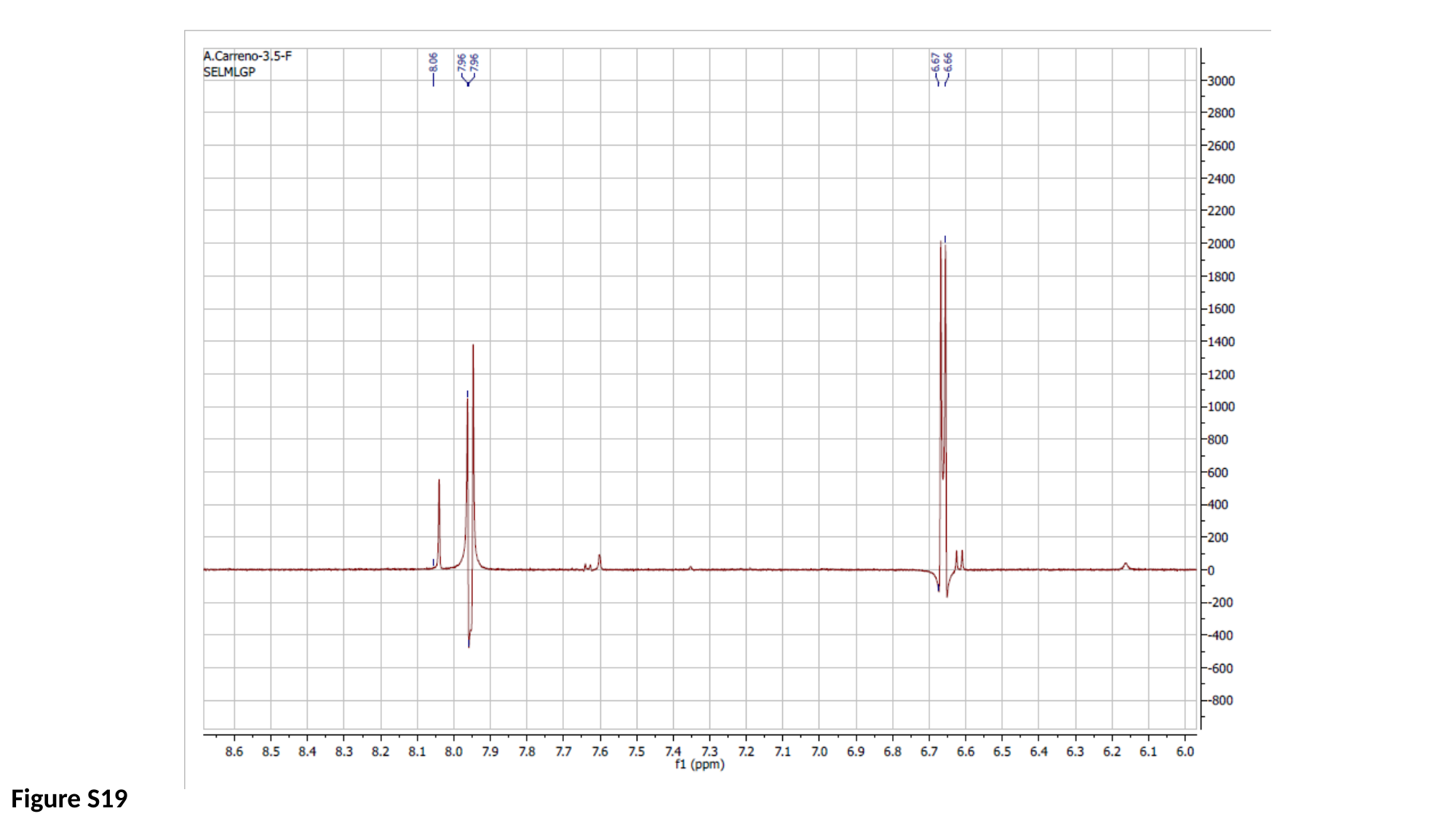

Figure S19

## Slide 20
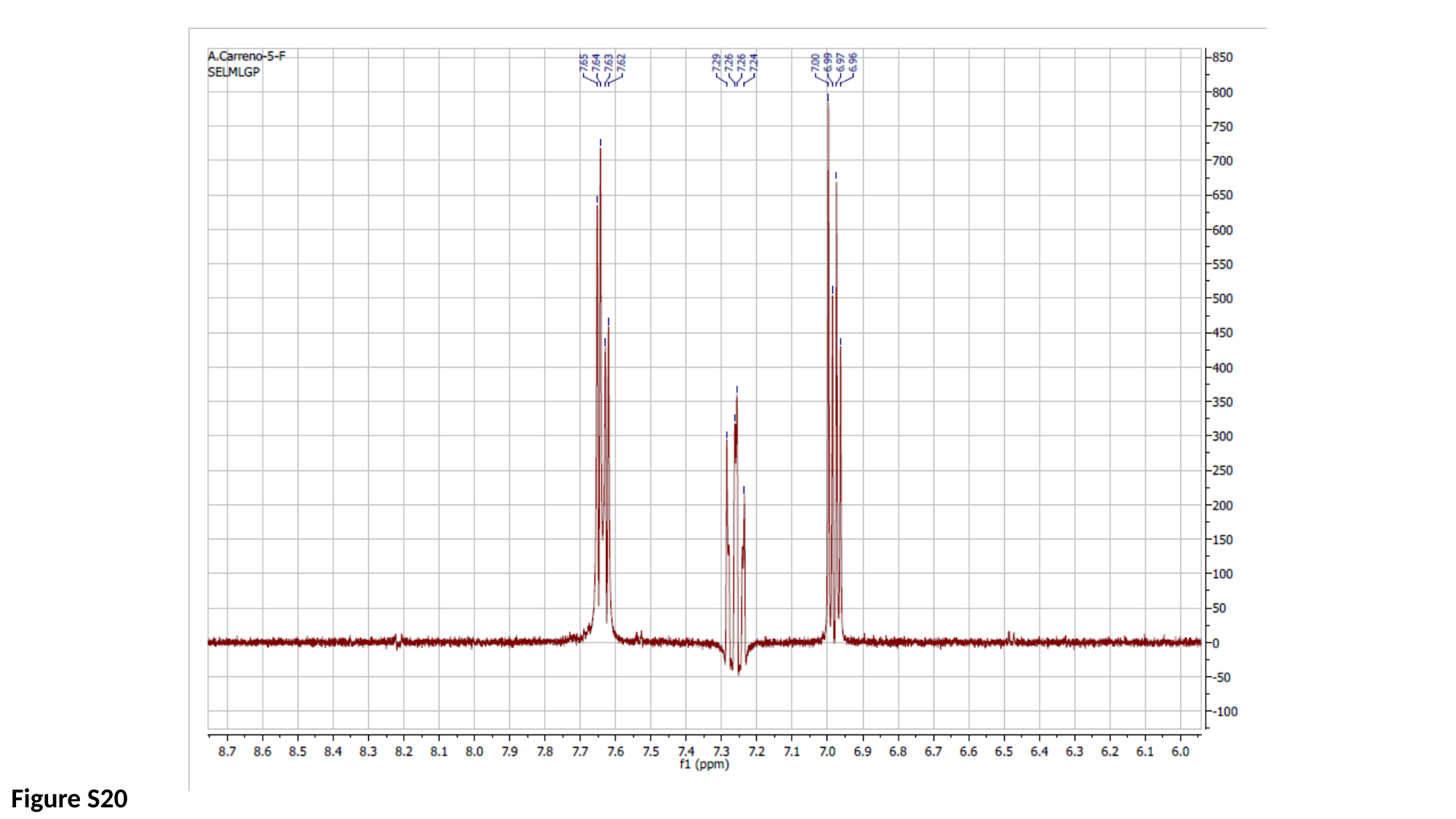

Figure S20

## Slide 21
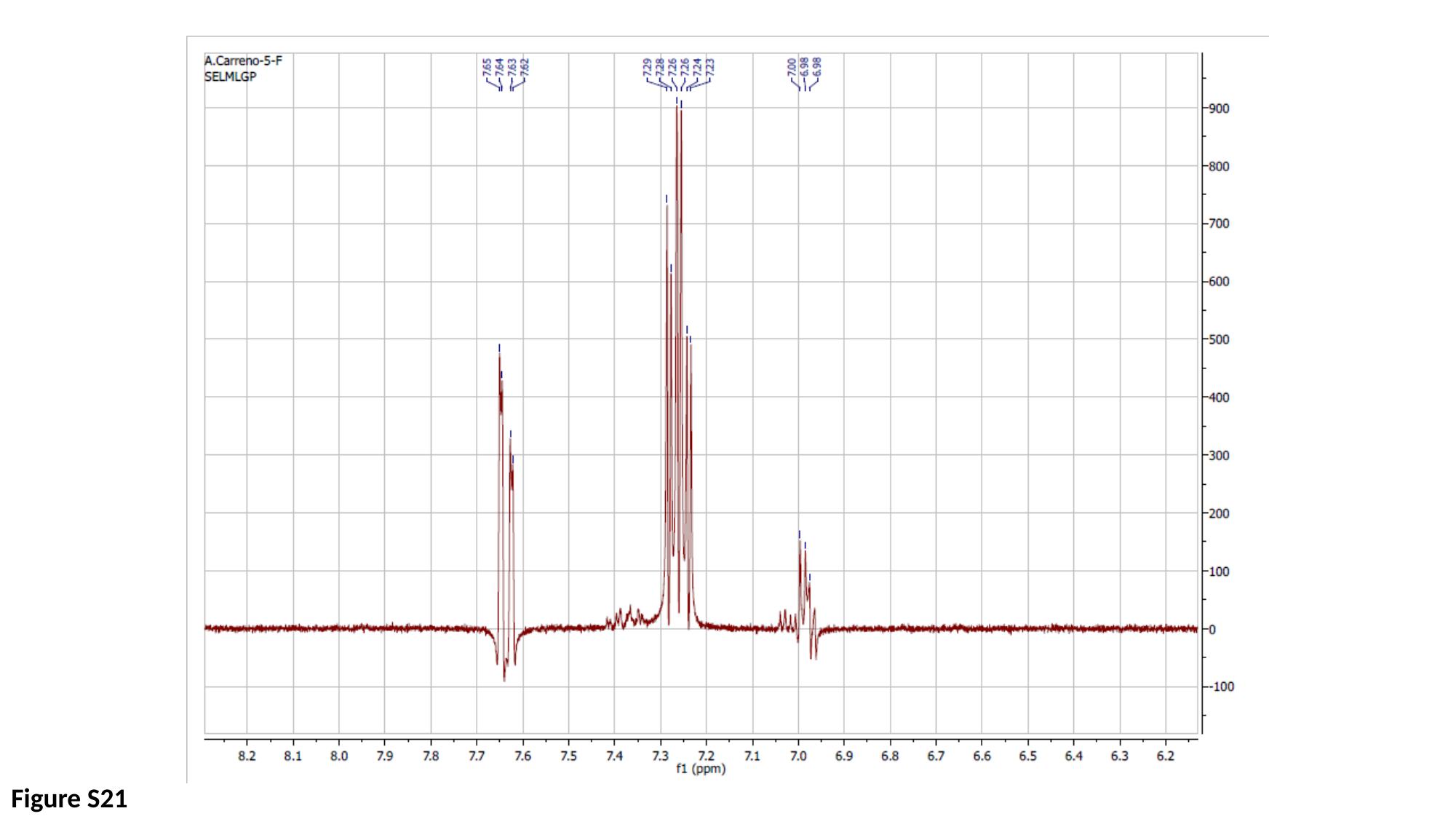

Figure S21

## Slide 22
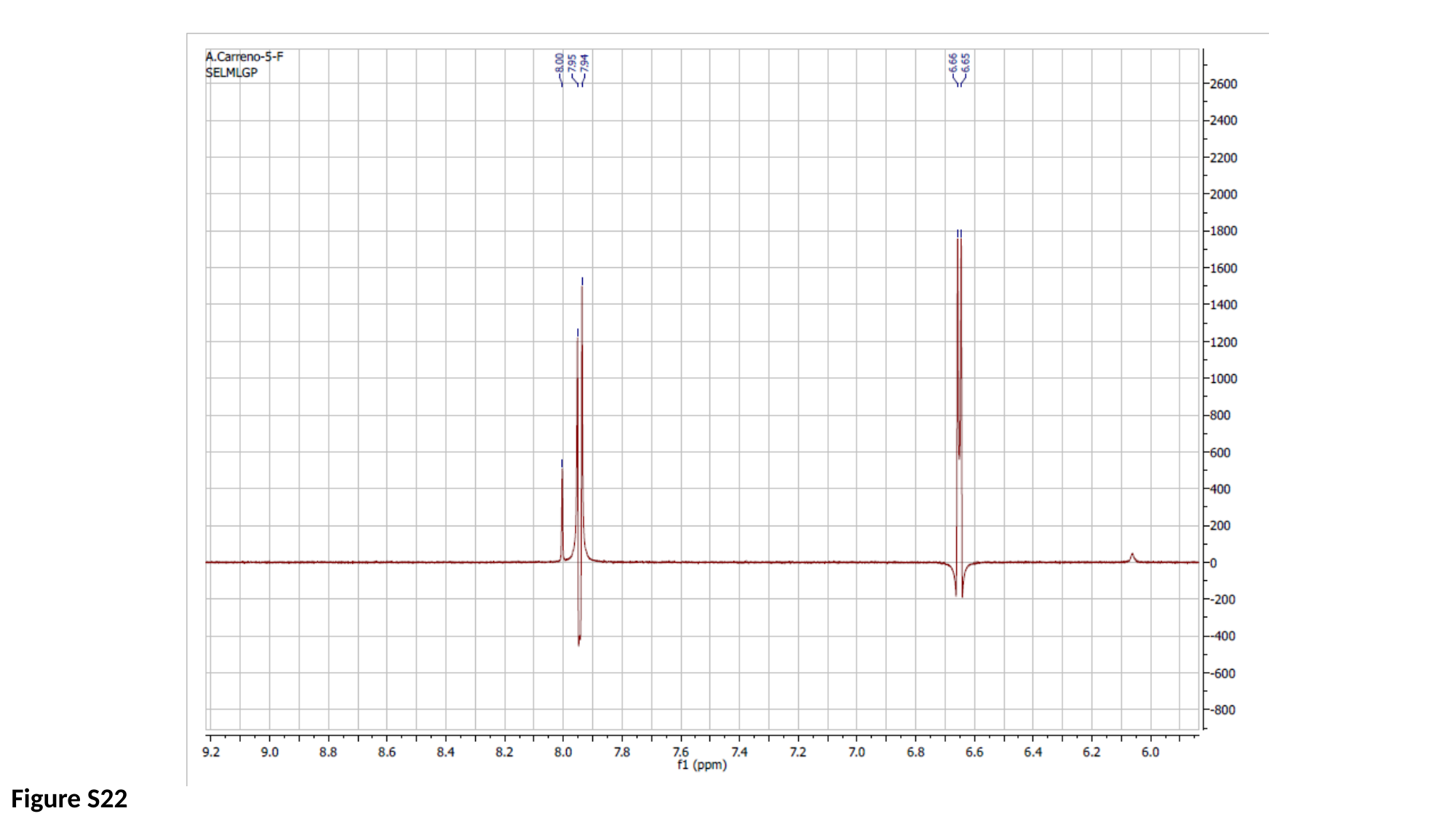

Figure S22

## Slide 23
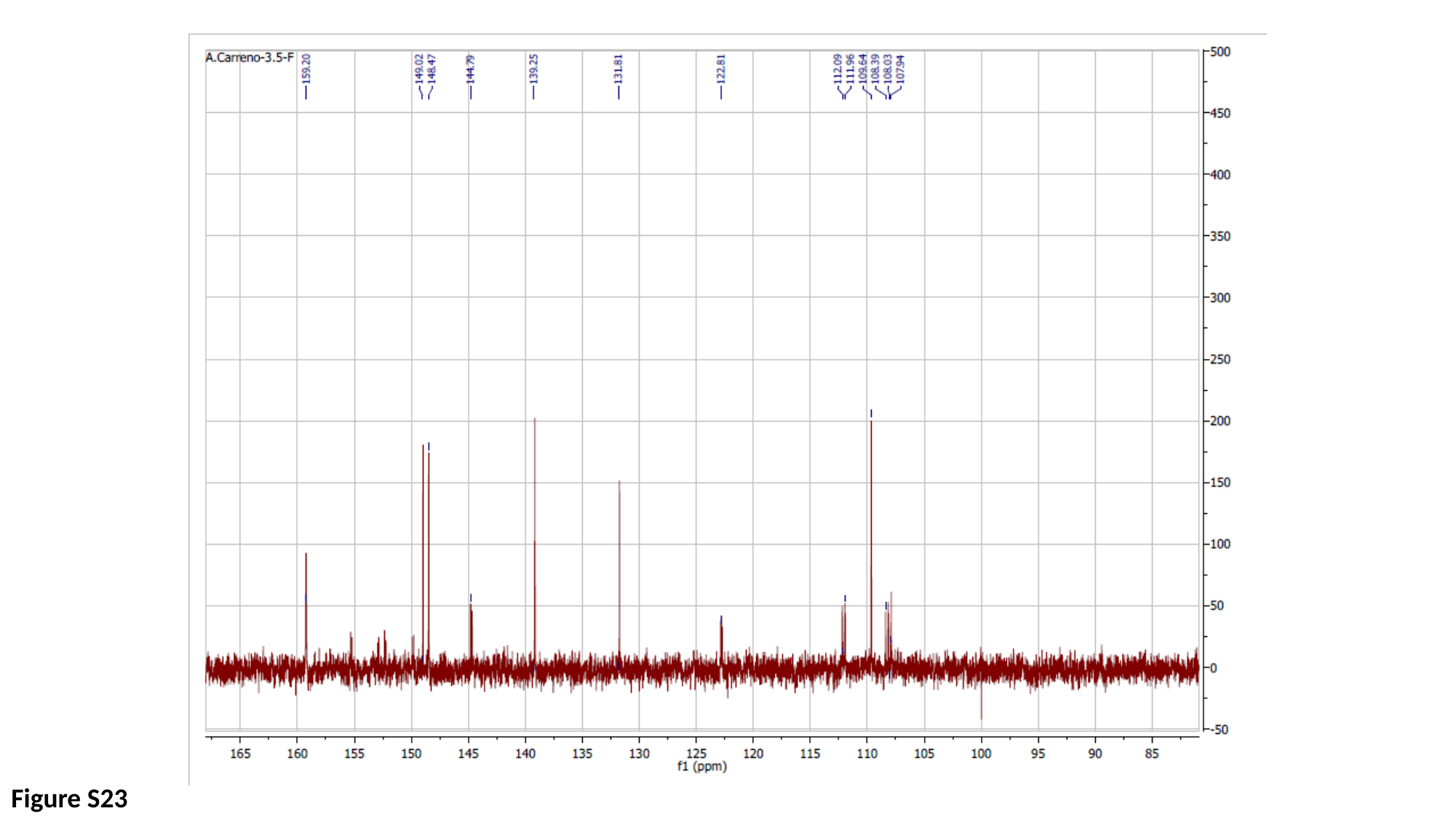

Figure S23

## Slide 24
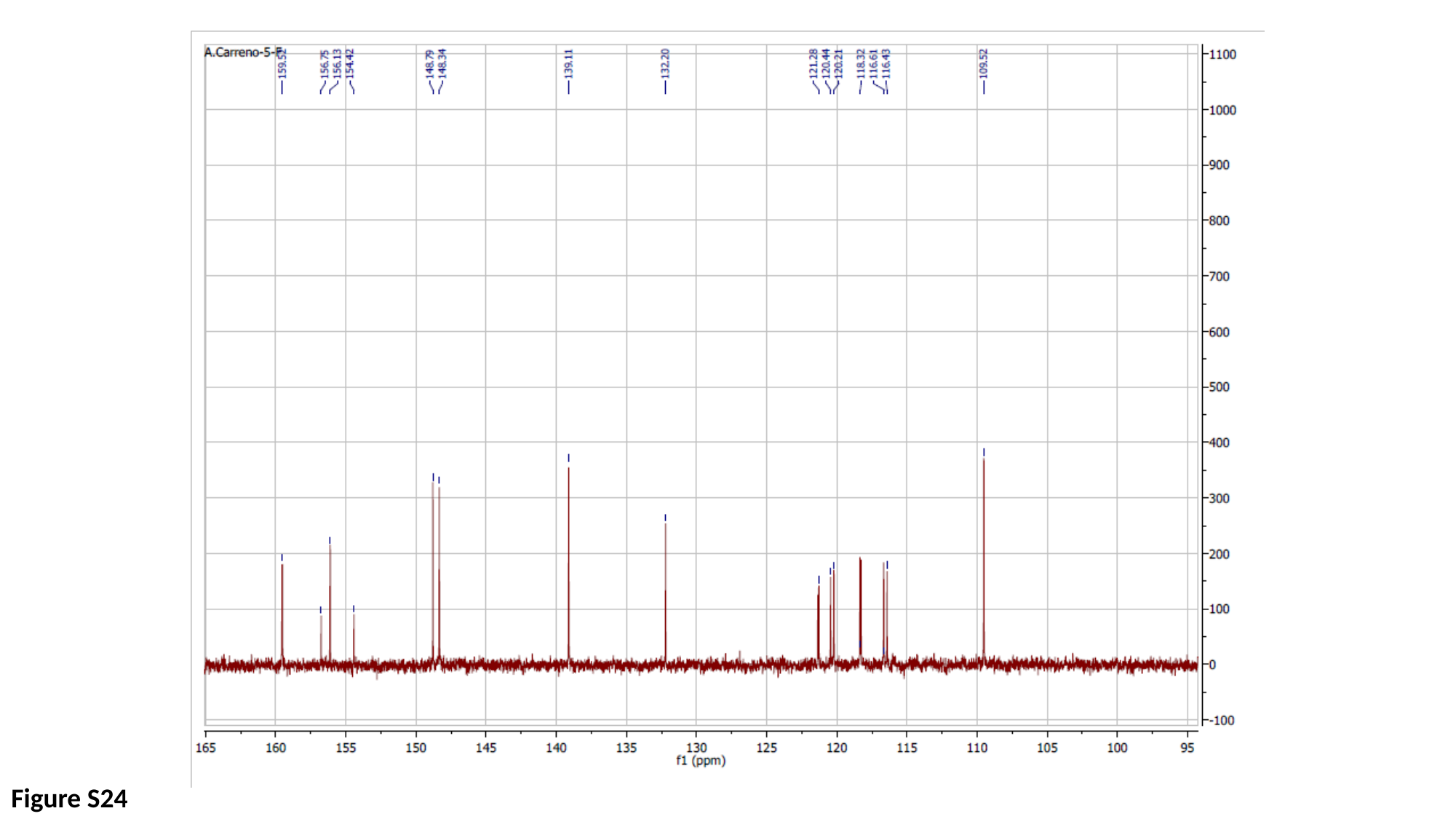

Figure S24

## Slide 25
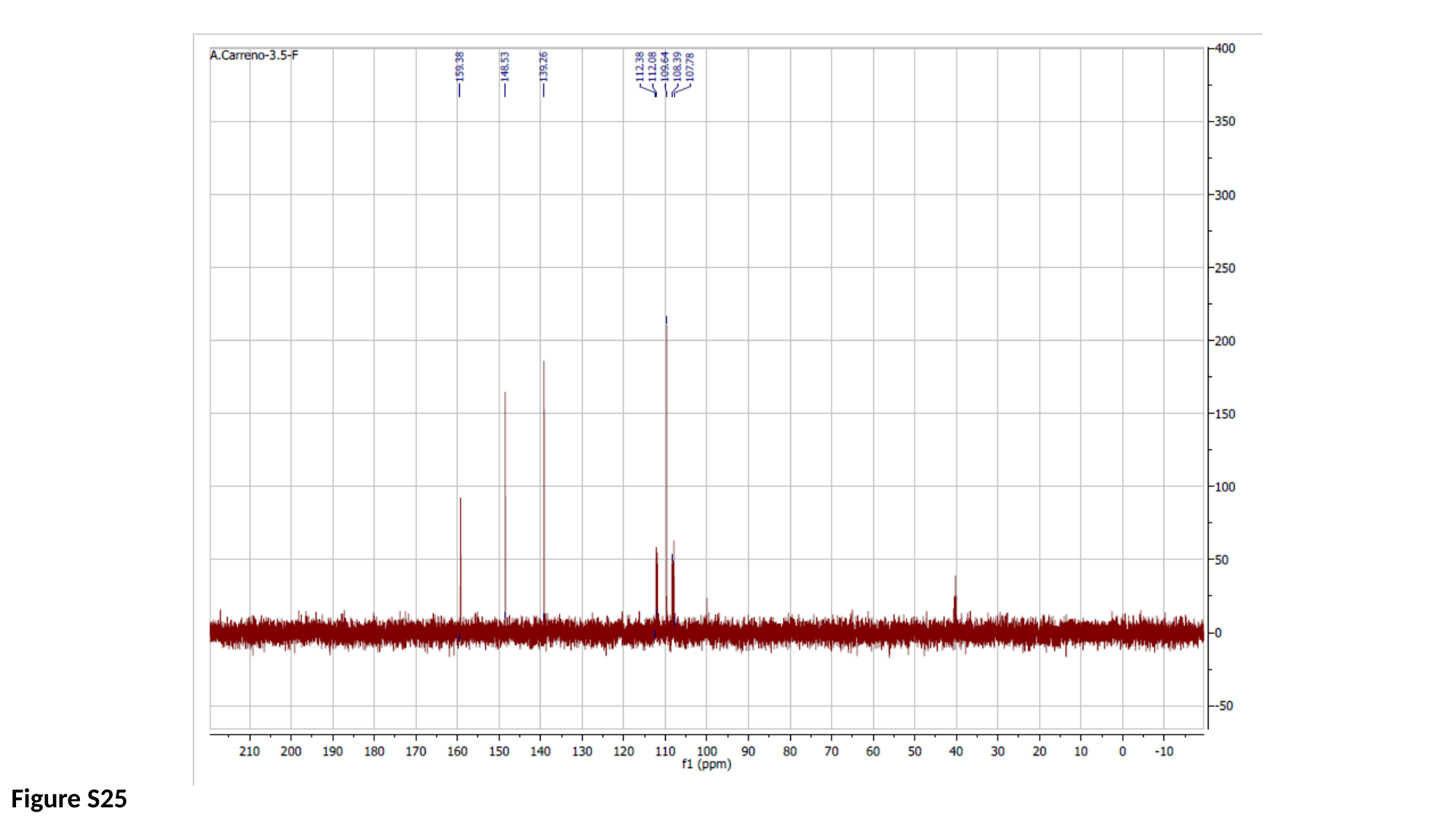

Figure S25

## Slide 26
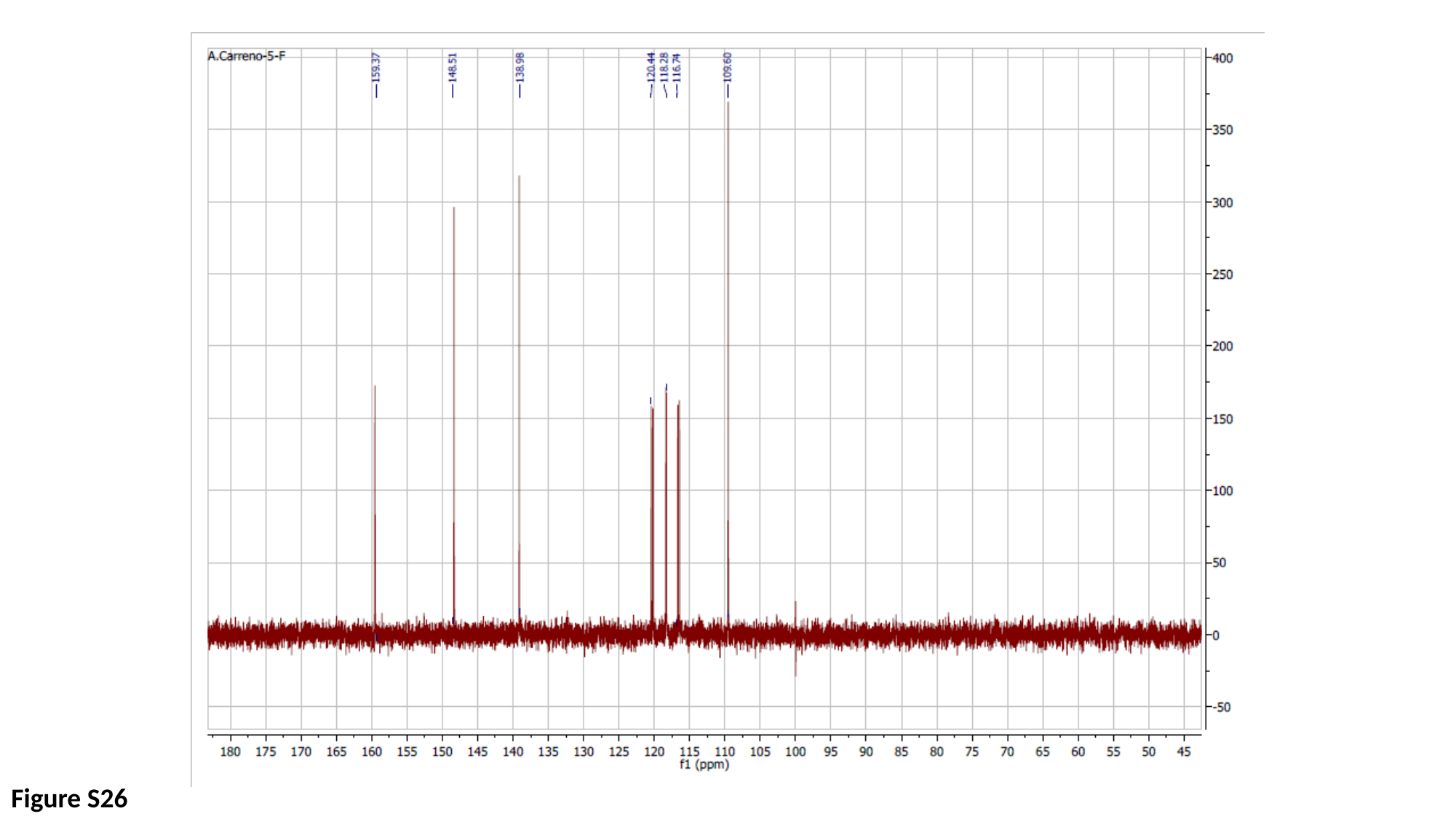

Figure S26

## Slide 27
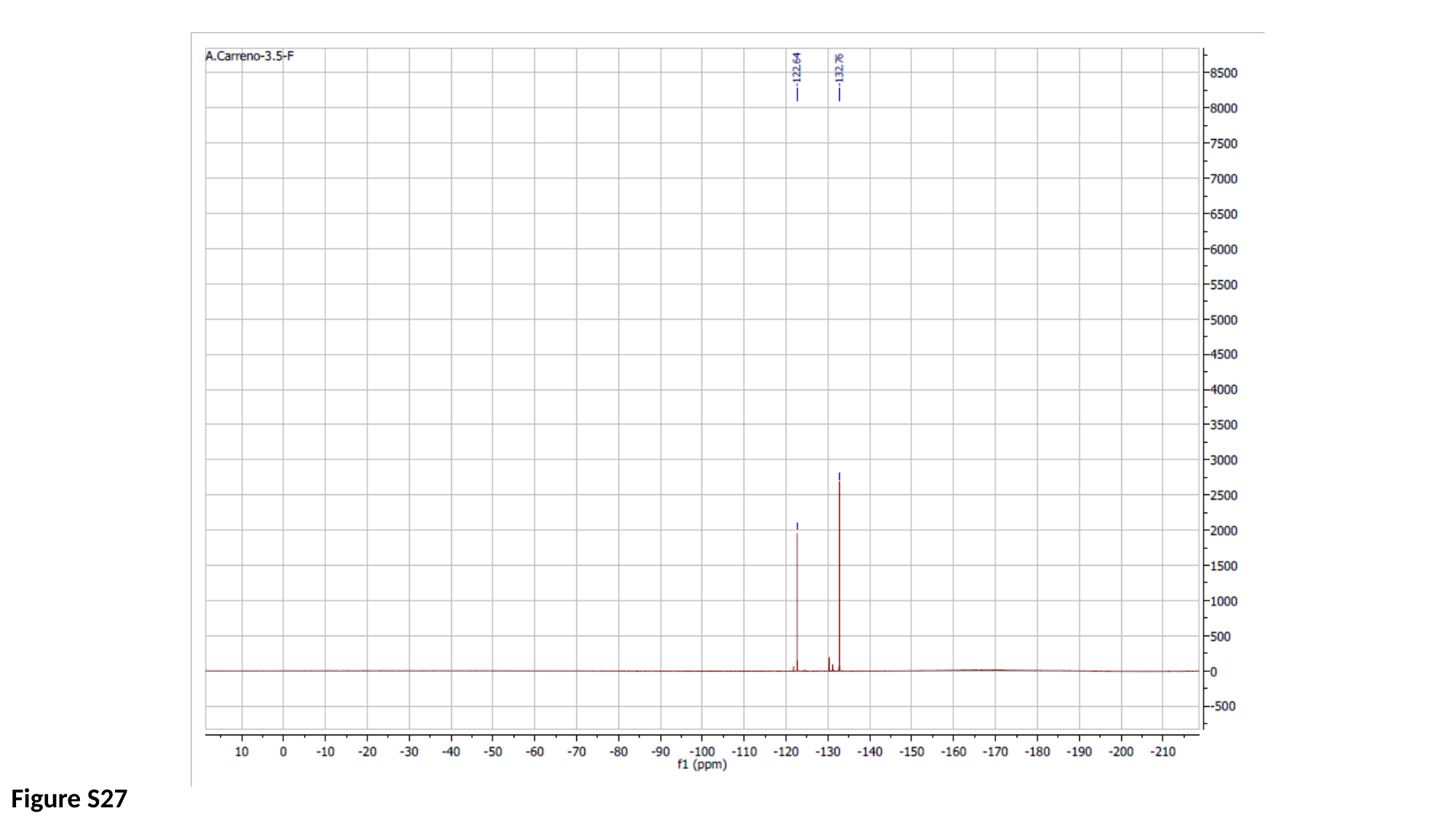

Figure S27

## Slide 28
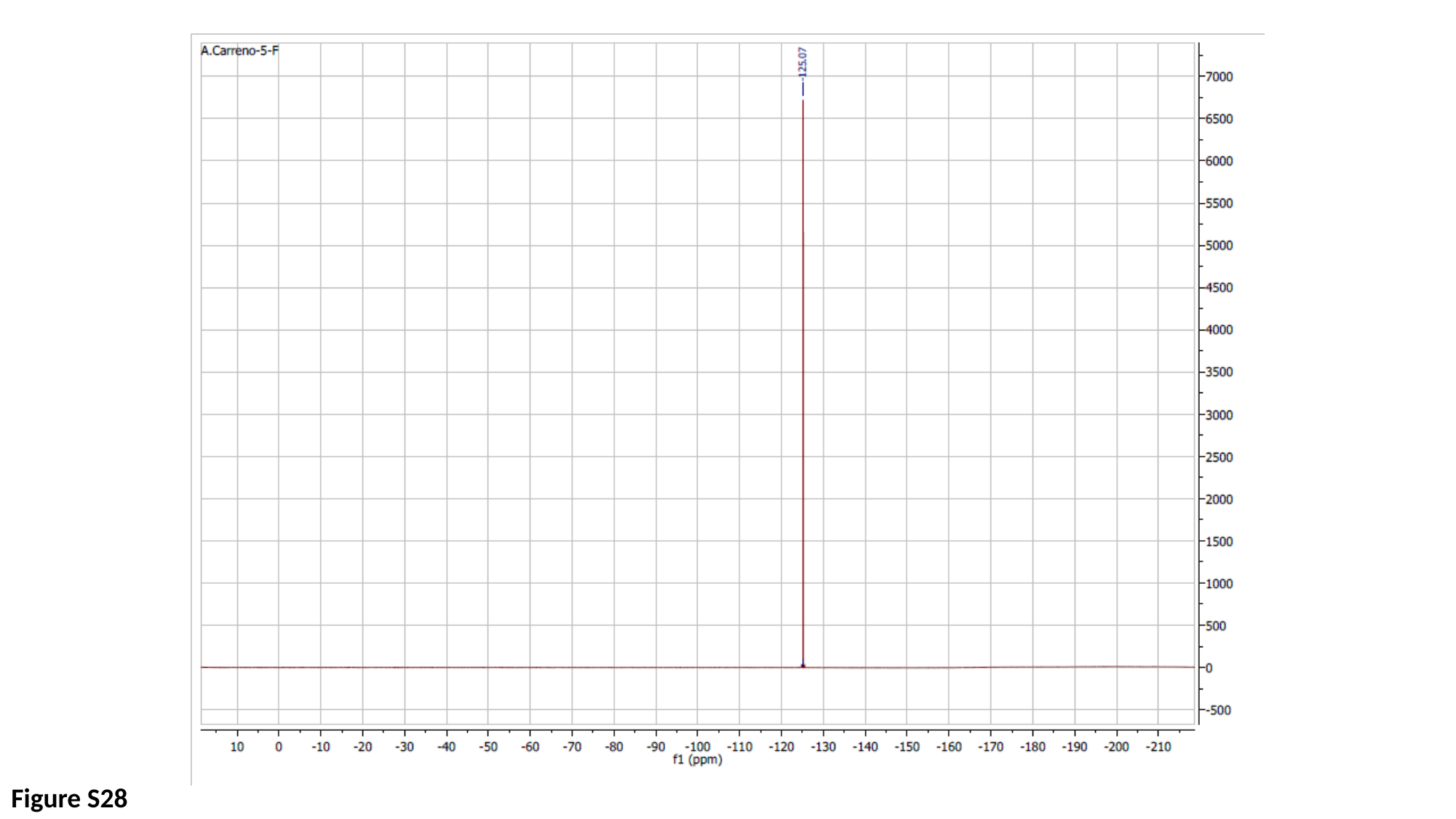

Figure S28

## Slide 29
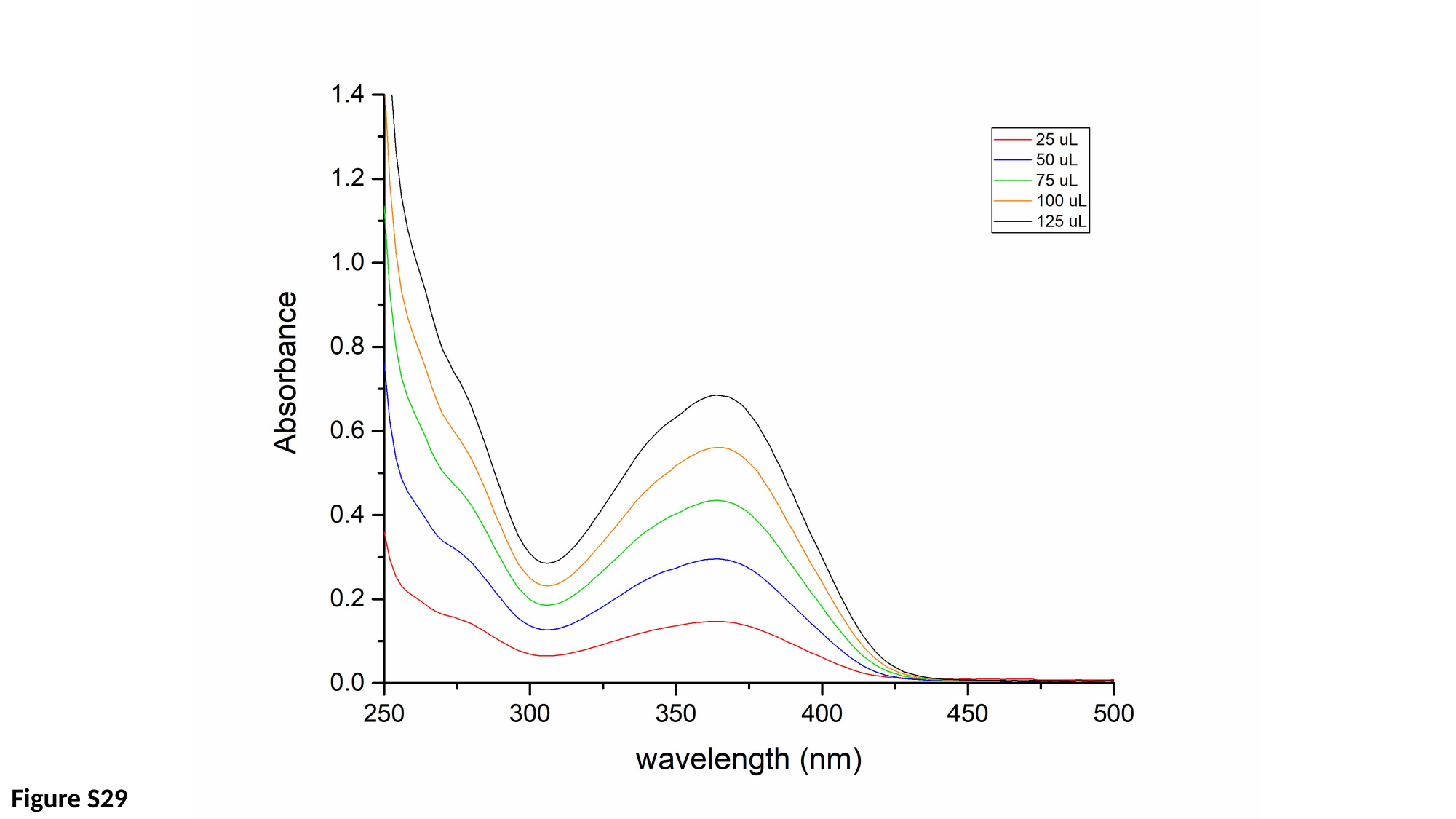

Figure S29

## Slide 30
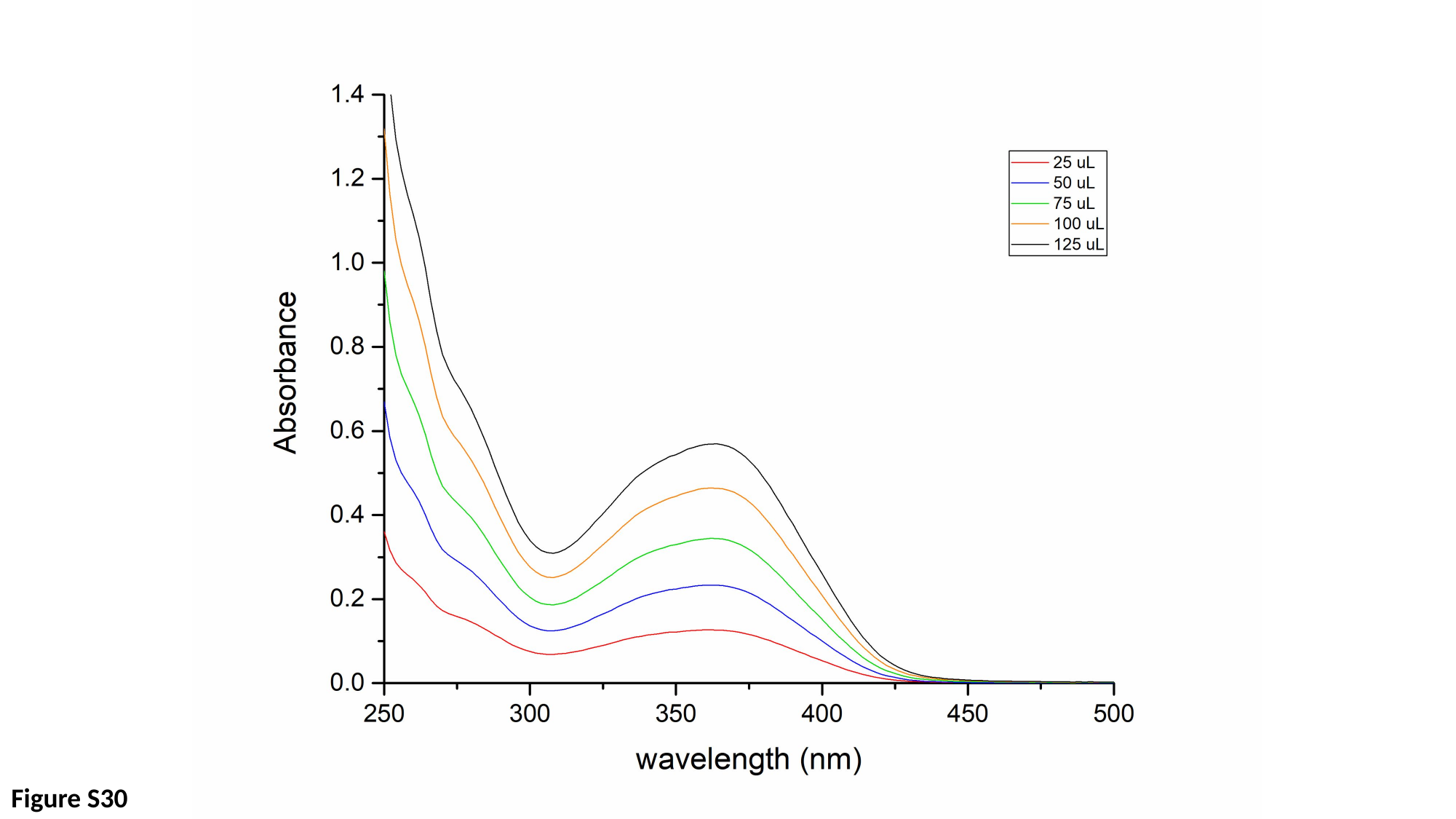

Figure S30

## Slide 31
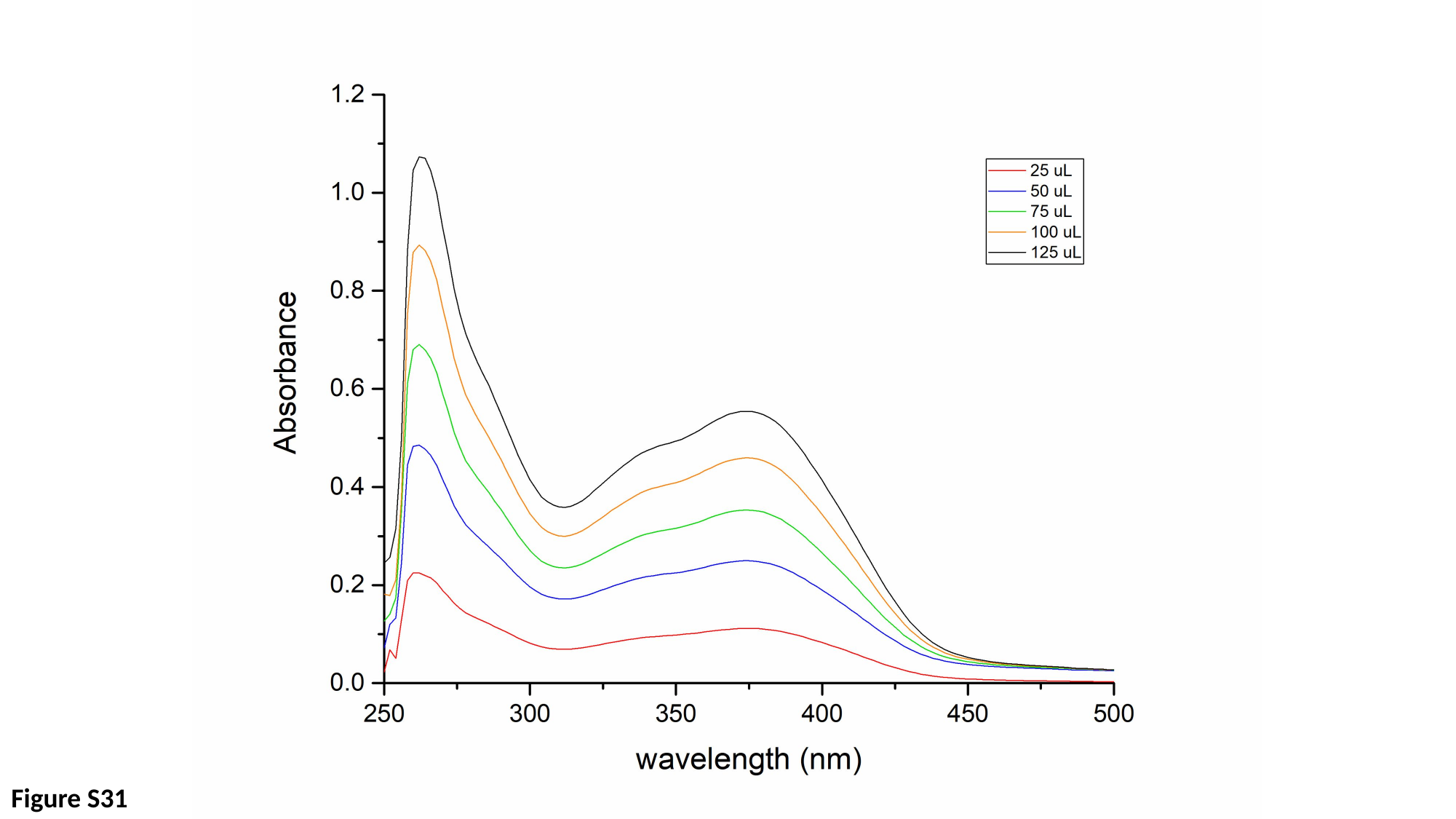

Figure S31

## Slide 32
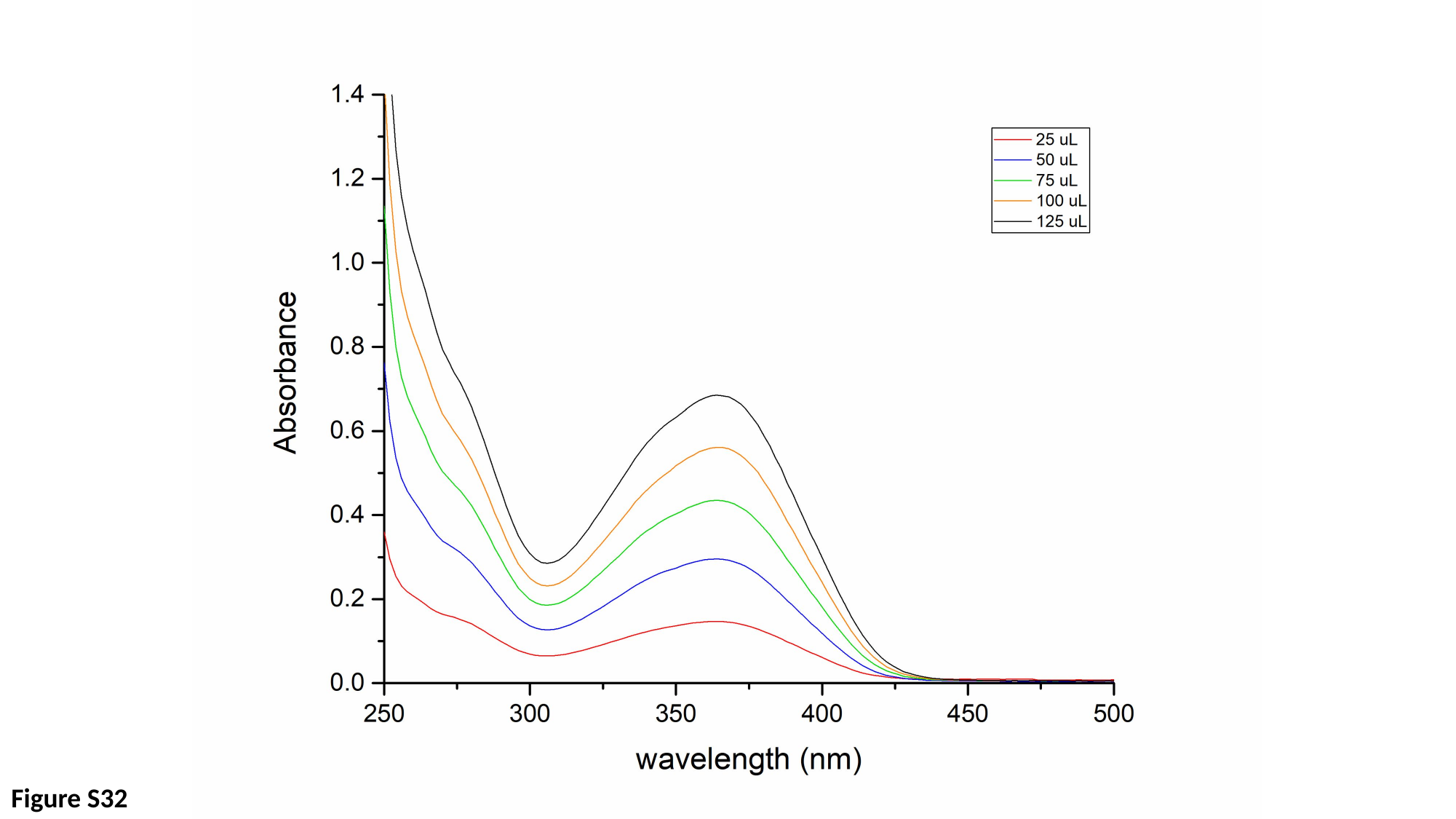

Figure S32

## Slide 33
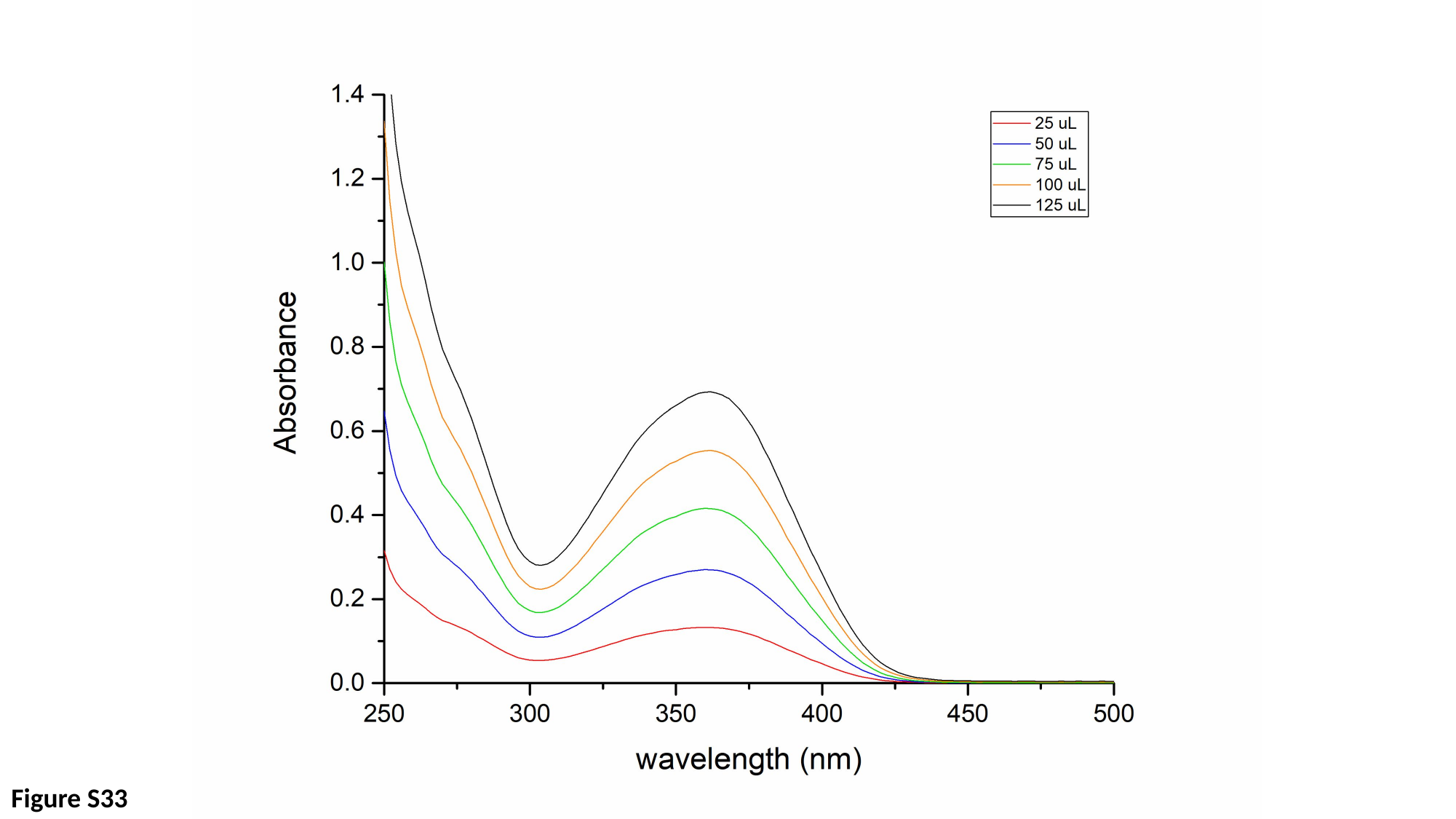

Figure S33

## Slide 34
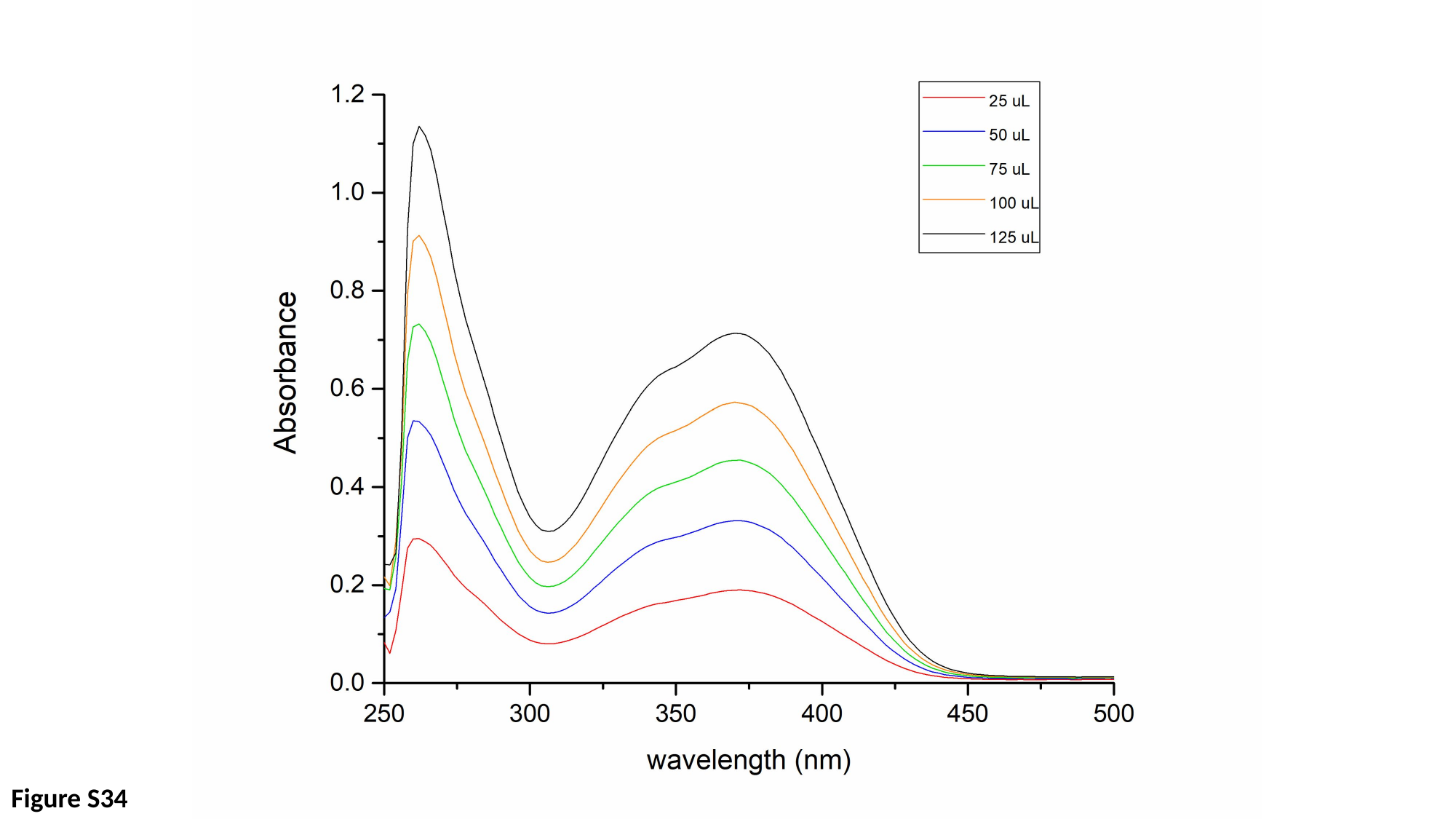

Figure S34

## Slide 35
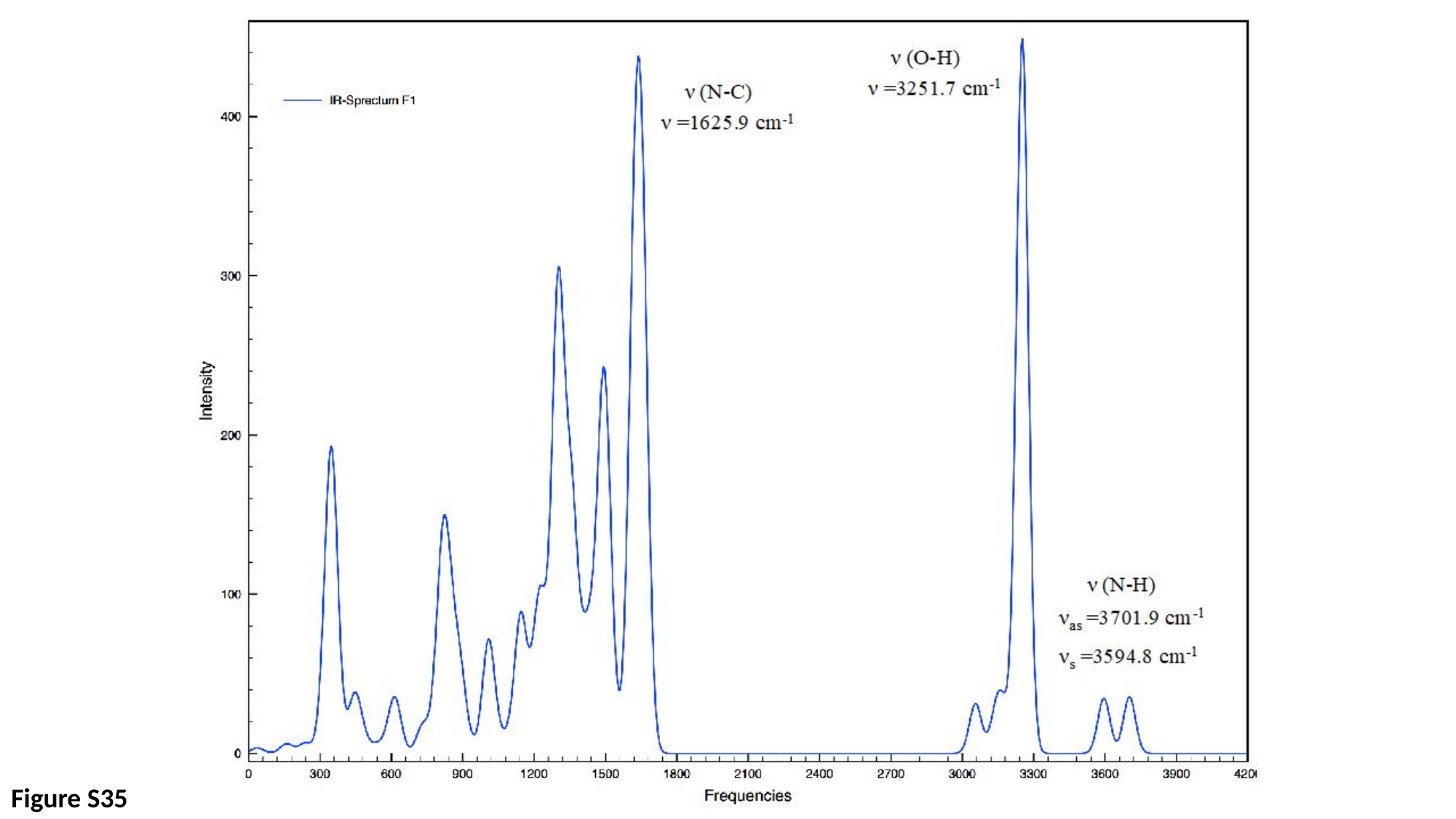

Figure S35

## Slide 36
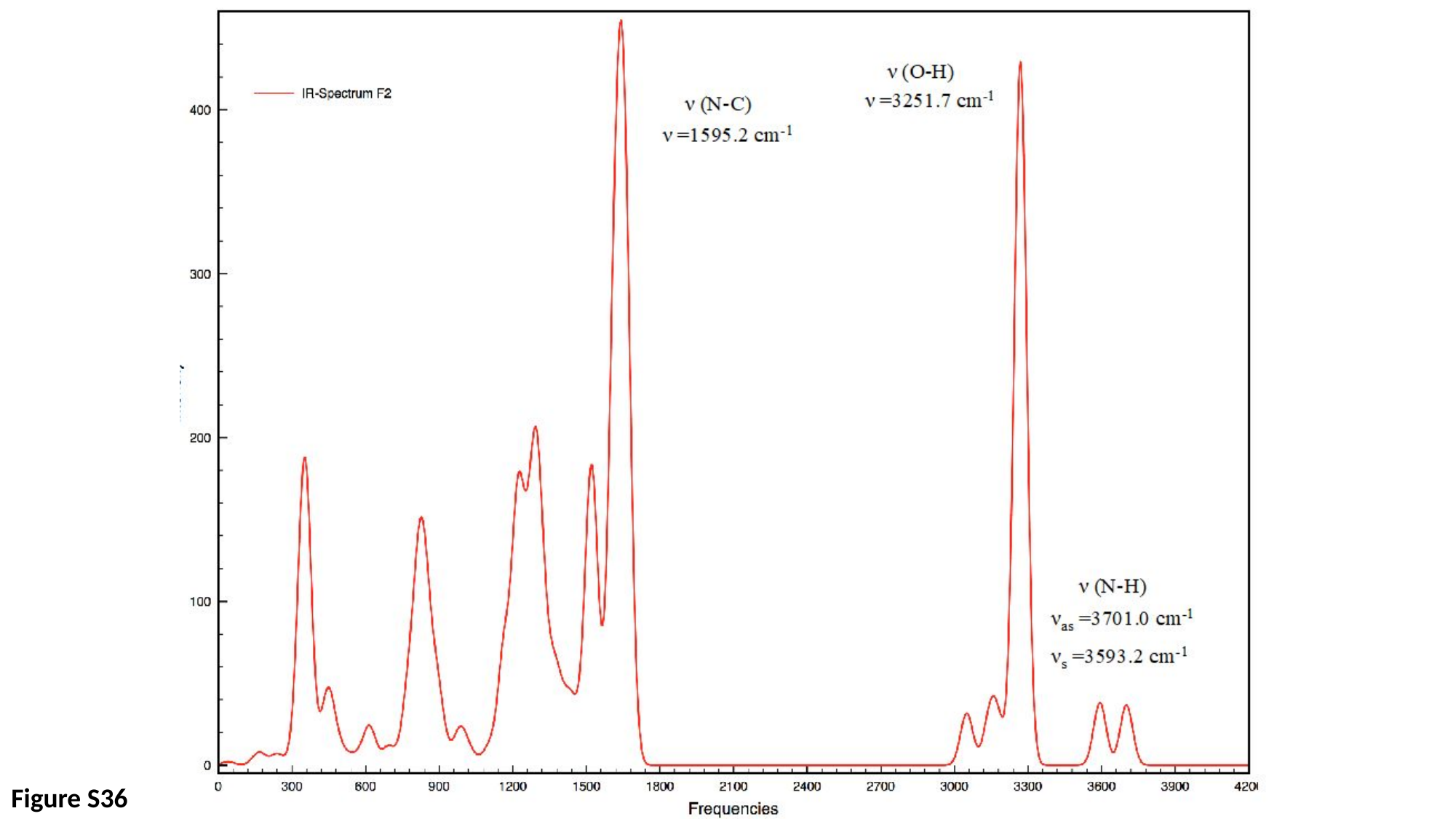

Figure S36

## Slide 37
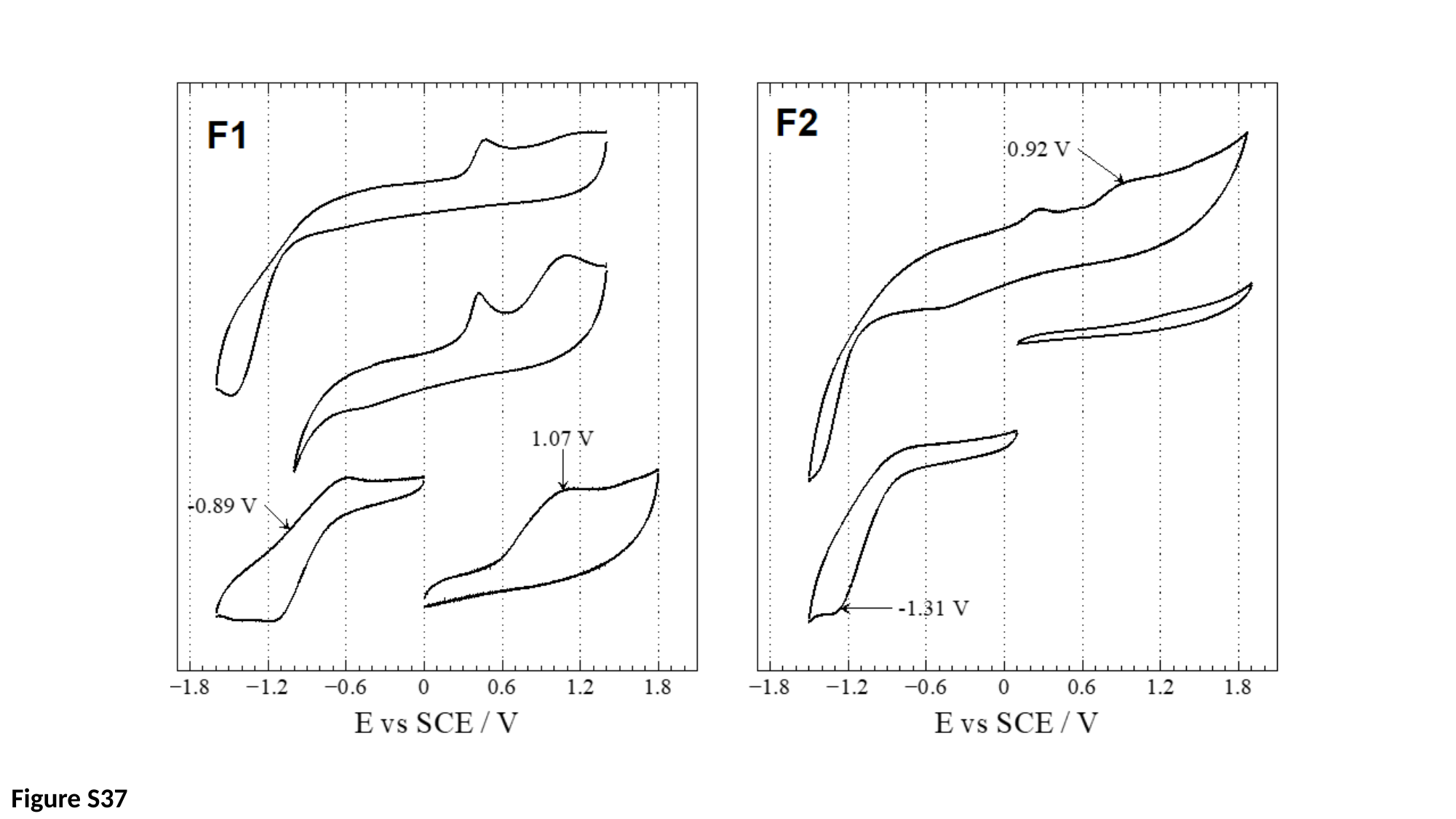

Figure S37

## Slide 38
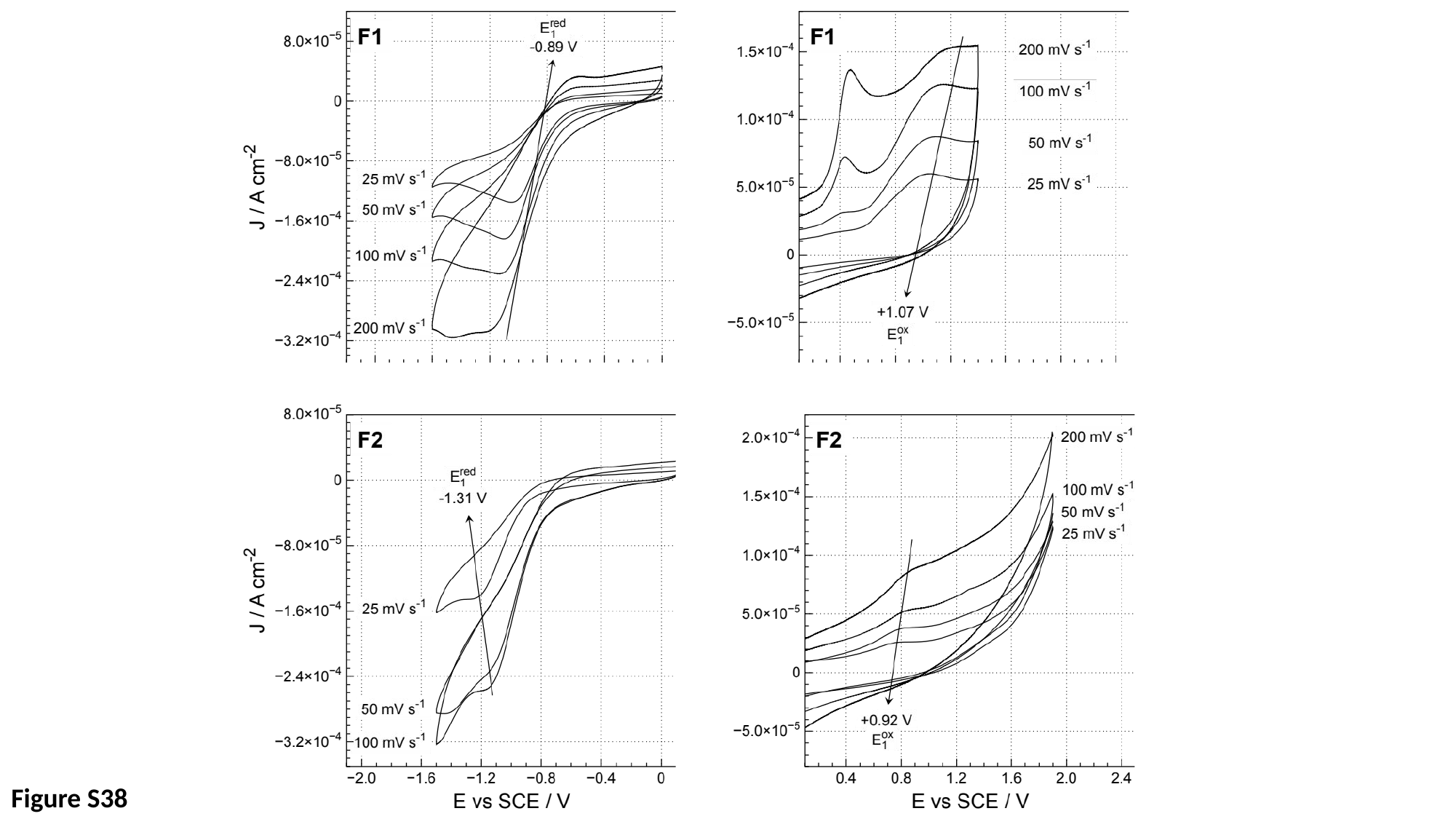

Figure S38

## Slide 39
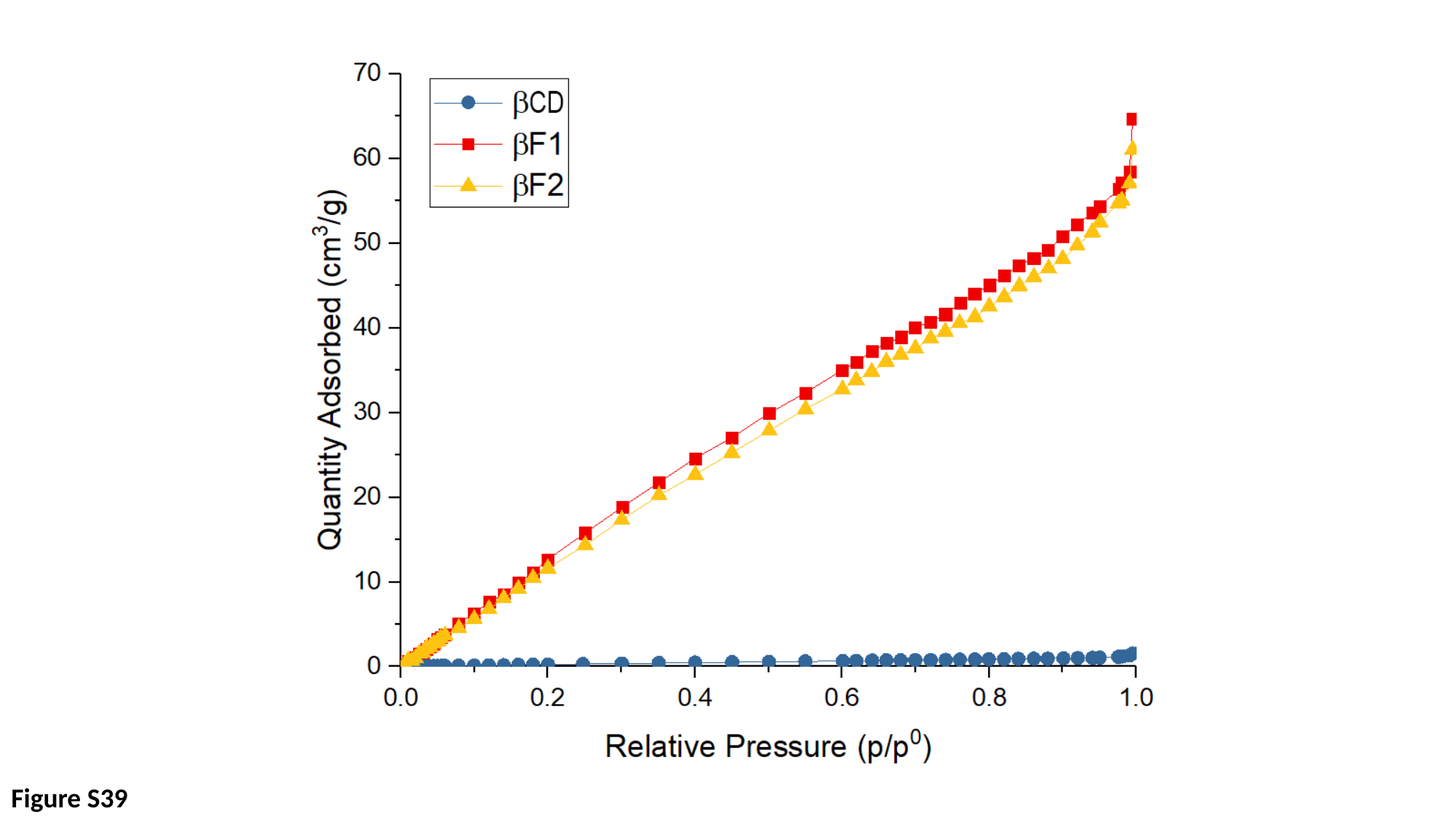

Figure S39
